# Supplementary material for: Outdoor roaming of owned cats elevates risk of zoonotic pathogen exposure: A global synthesis
Source: PLoS Pathog. 2026 Apr 20;22(4):e1014160. doi: 10.1371/journal.ppat.1014160 (PMC13128103; doi:10.1371/journal.ppat.1014160)
Supplement: S1 Table — Cells are left blank where no prevalence data were identified for a given pathogen–lifestyle combination. (DOCX) [file ppat.1014160.s001.docx]

**S1 Table.** Summary of pathogen prevalence (%) with 95% confidence intervals and total number of domestic cats sampled (n), stratified by lifestyle category (feral, outdoor-owned, indoor, shelter, and unknown). Cells are left blank where no prevalence data were identified for a given pathogen–lifestyle combination.

| **Pathogen** | **Feral** | **Indoor** | **Outdoor** | **Owned - Unknown** | **Shelter** |
| --- | --- | --- | --- | --- | --- |
| a) Bacteria | | | | | |
| Anaplasma | 3.5 (2.0–6.2;n=312) | 9.1 (5.8–14.1;n=187) | 7.9 (6.5–9.6;n=1126) | 1.4 (1.1–1.7;n=5121) |  |
| Bartonella | 34.8 (33.5–36.1;n=5451) | 12.4 (10.6–14.4;n=1180) | 22.5 (20.8–24.2;n=2271) | 17.5 (16.7–18.4;n=7042) | 8.7 (6.6–11.2;n=578) |
| Borrelia | 12.9 (6.9–22.7;n=70) | 0.0 (0.0–0.0;n=124) | 3.3 (2.2–4.9;n=725) | 3.3 (0.9–11.4;n=60) | 4.8 (1.3–15.8;n=42) |
| Borrellia |  |  | 100.0 (34.2–100.0;n=2) |  |  |
| Brucella | 4.5 (1.9–10.0;n=112) |  | 12.9 (6.9–22.7;n=70) | 0.0 (0.0–0.0;n=98) |  |
| Campylobacter | 17.4 (12.3–24.2;n=155) | 15.9 (9.5–25.3;n=82) | 18.9 (14.2–24.6;n=217) | 25.8 (23.9–27.8;n=1937) | 16.1 (12.1–21.2;n=248) |
| Chlamydia | 9.8 (8.2–11.8;n=1028) | 0.0 (0.0–0.0;n=33) | 19.2 (12.0–29.3;n=78) | 3.2 (2.8–3.6;n=7023) |  |
| Coxiella | 26.2 (22.4–30.4;n=454) |  | 9.8 (6.4–14.7;n=204) | 0.0 (0.0–0.0;n=73) |  |
| Ehrlichia | 2.7 (1.8–4.0;n=888) | 17.5 (10.0–28.6;n=63) | 15.8 (11.2–21.9;n=177) | 4.1 (2.9–5.8;n=725) |  |
| Francisella |  |  | 100.0 (20.7–100.0;n=1) |  |  |
| Hemoplasma | 34.2 (30.1–38.6;n=482) | 7.8 (5.6–10.7;n=435) | 15.9 (13.6–18.5;n=868) | 21.1 (18.5–24.0;n=861) | 8.4 (5.2–13.4;n=178) |
| Lawsonia |  |  | 12.5 (4.3–31.0;n=24) |  |  |
| Leptospira | 15.2 (13.9–16.7;n=2683) | 5.8 (3.8–8.8;n=346) | 13.1 (11.8–14.4;n=2561) | 14.1 (12.1–16.4;n=1015) | 19.9 (15.9–24.7;n=316) |
| Mycobacterium |  |  | 97.6 (94.0–99.1;n=168) |  |  |
| Mycoplasma | 16.5 (14.8–18.4;n=1673) | 4.8 (2.8–8.2;n=251) | 12.2 (6.8–21.0;n=82) | 7.9 (6.4–9.7;n=1035) |  |
| Orientia |  |  |  | 28.3 (22.4–35.2;n=187) |  |
| Rickettsia | 20.0 (17.3–22.8;n=812) | 34.9 (24.3–47.2;n=63) | 25.9 (21.7–30.5;n=379) | 12.3 (10.7–14.2;n=1306) | 15.1 (12.1–18.7;n=456) |
| Salmonella |  |  | 46.9 (33.7–60.6;n=49) | 1.0 (0.3–3.0;n=293) | 0.7 (0.1–3.7;n=149) |
| Yersinia |  |  | 100 (94.5–100.0;n=66) | 4.6 (1.6–12.7;n=65) |  |
| b) Fungal | | | | | |
| Blastocystis | 8.2 (6.6–10.0;n=969) |  |  | 16.2 (13.8–19.1;n=739) | 29.4 (22.8–37.1;n=153) |
| Enterocytozoon | 5.6 (1.9–15.1;n=54) |  |  | 4.9 (2.9–8.3;n=263) |  |
| Rhinosporidium |  |  |  | 50.0 (36.4–63.6;n=48) |  |
| Sporothrix |  |  | 70.1 (62.2–77.0;n=144) |  |  |
| c) Helminth | | | | | |
| Acanthocephalan | 5.8 (3.8–8.9;n=327) |  | 1.9 (0.5–6.8;n=103) |  |  |
| Acanthotrema | 2.7 (1.1–6.1;n=188) |  |  |  |  |
| Aelurostrongylus | 7.2 (6.4–8.2;n=3175) | 3.6 (2.9–4.5;n=1940) | 8.3 (7.6–9.1;n=5061) | 3.1 (2.7–3.4;n=9283) | 3.0 (2.2–4.2;n=1149) |
| Alaria | 11.2 (9.7–12.8;n=1531) |  |  | 2.8 (2.3–3.4;n=3832) | 0.6 (0.2–2.2;n=331) |
| Amoeba | 5.7 (2.9–10.9;n=140) |  |  | 32.2 (26.5–38.5;n=230) |  |
| Amphimerus |  |  |  | 71.4 (45.4–88.3;n=14) |  |
| Ancylostoma | 19.2 (18.4–20.0;n=9634) | 1.6 (1.1–2.4;n=1380) | 7.4 (6.4–8.7;n=1948) | 5.0 (4.7–5.4;n=12337) | 4.6 (3.8–5.5;n=2422) |
| Aonchotheca | 7.1 (3.5–13.9;n=99) |  |  | 0.2 (0.1–0.7;n=1000) | 1.0 (0.3–3.4;n=208) |
| Ascarids | 5.4 (1.5–17.7;n=37) |  |  | 16.4 (14.9–18.1;n=2015) |  |
| Ascocotyle | 0.4 (0.1–2.3;n=240) |  |  |  |  |
| Brugia |  |  | 4.1 (2.1–7.8;n=196) |  |  |
| Capillaria | 7.3 (6.5–8.2;n=3703) | 0.1 (0.0–0.8;n=699) | 2.7 (2.2–3.3;n=2995) | 1.3 (1.1–1.6;n=11017) | 2.3 (1.7–3.2;n=1534) |
| Centrocestus | 2.1 (0.8–5.3;n=188) |  |  |  |  |
| Centrorhynchus | 3.4 (1.5–7.8;n=146) |  |  |  |  |
| Cestodes | 18.7 (15.4–22.6;n=459) | 2.1 (0.4–10.9;n=48) | 8.8 (5.1–14.7;n=137) |  |  |
| Cryptocotyle | 2.4 (1.2–4.9;n=287) |  |  |  |  |
| Cylicospirura | 8.5 (6.0–11.8;n=366) |  |  |  |  |
| Dioctophyme |  |  |  | 100.0 (34.2–100.0;n=2) |  |
| Diphylobothrium | 15.9 (11.7–21.3;n=226) |  |  | 1.5 (0.8–2.7;n=678) | 0.0 (0.0–0.0;n=197) |
| Dipylidium | 18.3 (17.4–19.1;n=8001) | 0.5 (0.2–1.2;n=996) | 3.4 (2.7–4.2;n=2160) | 1.4 (1.2–1.6;n=13236) | 6.0 (4.8–7.4;n=1253) |
| Dirofilaria | 9.1 (7.4–11.2;n=886) |  |  | 3.8 (1.6–8.6;n=132) |  |
| Echinochasmus | 5.4 (3.6–7.9;n=428) |  |  |  |  |
| Echinococcus |  |  | 4.6 (2.9–7.2;n=370) | 0.3 (0.2–0.4;n=10781) |  |
| Echinoparyphium | 0.5 (0.1–3.0;n=188) |  |  |  |  |
| Echinostoma | 0.6 (0.2–1.8;n=488) |  |  |  |  |
| Endoparasites | 29.8 (23.5–36.9;n=178) | 73.5 (67.4–78.8;n=230) | 41.1 (38.0–44.3;n=946) |  | 22.2 (13.2–34.9;n=54) |
| Galactosomum | 0.4 (0.1–2.3;n=240) |  |  |  |  |
| Gnathostoma | 1.1 (0.4–2.9;n=354) |  |  |  |  |
| Gurltia |  | 42.9 (26.5–60.9;n=28) | 51.6 (39.6–63.4;n=64) |  |  |
| Haplorchis | 2.1 (1.1–3.8;n=480) |  |  |  |  |
| Helminth |  | 6.9 (4.2–11.3;n=202) | 31.3 (27.4–35.6;n=485) |  |  |
| Hepaticola | 4.8 (1.9–11.7;n=83) |  |  |  |  |
| Heterophyes | 10.8 (9.0–12.9;n=965) |  |  |  |  |
| Heterophyids | 8.1 (5.5–11.9;n=283) |  |  |  |  |
| Heterophyopsis | 2.7 (1.1–6.1;n=188) |  |  |  |  |
| Hydatigera | 30.8 (24.5–37.8;n=182) |  |  |  |  |
| Hymenolepis | 0.0 (0.0–0.0;n=300) |  |  | 0.0 (0.0–0.0;n=25) |  |
| Joyeuxiella | 46.2 (43.0–49.5;n=900) | 1.3 (0.5–3.9;n=224) | 1.2 (0.7–2.1;n=926) |  |  |
| Lagochilascaris | 50.0 (18.8–81.2;n=6) |  | 100.0 (34.2–100.0;n=2) |  |  |
| Mesocestoides | 8.3 (6.5–10.5;n=745) |  |  | 0.2 (0.1–0.4;n=2904) | 1.0 (0.3–2.9;n=295) |
| Mesostephanus | 0.9 (0.4–2.0;n=650) |  |  |  |  |
| Metagonimus | 32.4 (26.2–39.4;n=188) |  |  |  |  |
| Microsporidia | 44.7 (35.4–54.3;n=103) |  |  |  |  |
| Moniliformis | 0.3 (0.1–1.7;n=339) |  | 0.0 (0.0–0.0;n=85) |  |  |
| Ollulanus | 9.8 (7.9–12.1;n=756) |  |  | 0.1 (0.0–0.3;n=2586) |  |
| Oncicola | 28.0 (23.0–33.5;n=279) |  |  |  |  |
| Opisthorchis | 11.1 (8.5–14.5;n=431) |  |  |  |  |
| Oslerus | 24.1 (15.0–36.5;n=58) |  |  |  |  |
| Pearsonema | 5.8 (2.7–12.1;n=103) |  |  |  |  |
| Physaloptera | 9.1 (8.2–10.0;n=4077) |  | 4.7 (1.8–11.5;n=85) | 0.6 (0.4–1.0;n=3023) | 0.0 (0.0–0.0;n=36) |
| Platynosomum | 10.6 (8.9–12.6;n=1092) |  |  | 1.9 (1.5–2.4;n=4096) |  |
| Pseudamphistomum | 0.6 (0.2–2.1;n=351) |  |  |  |  |
| Pterygodermatites | 3.6 (0.6–17.7;n=28) |  |  |  |  |
| Pygidiopsis | 7.9 (5.7–10.9;n=428) |  |  |  |  |
| Rictularia | 18.0 (14.5–22.1;n=389) |  |  |  |  |
| Schistosoma |  |  |  | 12.1 (7.7–18.5;n=141) |  |
| Spirocerca | 6.7 (3.3–13.1;n=105) |  |  | 0.8 (0.1–4.2;n=131) |  |
| Spirometra | 11.2 (9.7–12.8;n=1595) | 1.0 (0.5–2.0;n=804) | 4.8 (2.4–9.2;n=167) | 1.3 (1.0–1.7;n=5095) | 3.3 (2.3–4.7;n=858) |
| Spiruroid | 12.5 (5.0–28.1;n=32) |  |  |  |  |
| Stellantchasmus | 2.3 (1.3–4.2;n=428) |  |  |  |  |
| Stictodora | 1.4 (0.6–3.0;n=428) |  |  |  |  |
| Strongyle | 6.3 (5.0–7.8;n=1211) | 1.1 (0.3–3.8;n=188) | 17.8 (14.7–21.4;n=499) | 1.5 (0.4–5.4;n=131) | 0.4 (0.1–2.1;n=260) |
| Strongyloides | 4.7 (3.2–6.9;n=514) |  |  |  |  |
| Syphacia | 2.0 (0.4–10.5;n=50) |  |  |  |  |
| Taenia | 13.2 (12.5–13.9;n=7983) | 0.6 (0.3–1.3;n=1107) | 2.4 (1.6–3.6;n=875) | 1.4 (1.2–1.7;n=13053) | 7.5 (5.8–9.6;n=774) |
| Thelazia |  |  | 0.8 (0.5–1.3;n=2172) | 23.5 (9.6–47.3;n=17) |  |
| Toxascaris | 6.6 (5.8–7.6;n=2763) | 0.3 (0.1–0.8;n=1131) | 1.6 (1.1–2.5;n=1226) | 0.7 (0.5–0.8;n=9497) | 0.8 (0.3–1.8;n=649) |
| Toxocara | 26.0 (25.2–26.8;n=11875) | 5.9 (5.2–6.6;n=4446) | 27.1 (25.8–28.5;n=4195) | 10.4 (9.9–10.9;n=16944) | 18.1 (16.6–19.7;n=2492) |
| Trematode | 6.4 (2.8–14.1;n=78) |  |  |  |  |
| Trichinella | 3.5 (1.0–11.9;n=57) |  |  |  |  |
| Trichuris | 4.3 (3.2–5.8;n=927) | 26.1 (12.5–46.5;n=23) | 5.0 (2.6–9.6;n=160) | 0.5 (0.3–0.9;n=3051) | 0.0 (0.0–0.0;n=2) |
| Troglostrongylus | 6.0 (3.2–11.0;n=150) | 1.5 (0.7–3.5;n=326) | 3.0 (2.4–3.9;n=1877) | 3.2 (2.3–4.5;n=1000) |  |
| Uncinaria | 1.5 (0.7–3.2;n=409) |  |  | 0.1 (0.0–0.3;n=2472) |  |
| c) Protozoa | | | | | |
| Babesia | 4.5 (2.5–7.9;n=245) | 6.4 (4.4–9.3;n=390) | 12.6 (10.6–14.8;n=956) | 1.5 (0.8–2.8;n=598) |  |
| Balantidium |  |  |  | 0.0 (0.0–0.0;n=25) |  |
| Besnoitia | 3.6 (0.6–17.7;n=28) |  |  |  |  |
| Chilomastix |  |  |  | 9.7 (7.8–12.1;n=732) |  |
| Coccidia | 8.8 (5.5–13.6;n=194) |  |  | 2.9 (1.7–4.9;n=449) |  |
| Cryptosporidium | 3.8 (3.1–4.8;n=2059) | 4.7 (4.1–5.5;n=3371) | 11.3 (9.3–13.6;n=835) | 3.6 (3.1–4.1;n=6244) | 5.2 (4.2–6.2;n=1901) |
| Cystoisospora | 12.1 (11.3–12.9;n=6423) | 3.8 (3.0–4.8;n=1801) | 6.6 (5.5–8.0;n=1536) | 6.8 (6.4–7.2;n=18529) | 8.3 (7.4–9.4;n=2961) |
| Cytauxzoon |  | 0.0 (0.0–0.0;n=67) |  | 24.4 (20.9–28.3;n=512) |  |
| Eimeria | 5.1 (1.4–16.9;n=39) |  |  | 0.0 (0.0–0.0;n=25) |  |
| Endolimax |  |  |  | 13.3 (3.7–37.9;n=15) |  |
| Entamoeba |  | 12.0 (4.2–30.0;n=25) | 2.9 (1.0–8.2;n=103) | 12.0 (4.2–30.0;n=25) |  |
| Giardia | 5.5 (4.9–6.1;n=5614) | 9.0 (8.1–10.0;n=3462) | 8.4 (7.4–9.7;n=2262) | 7.0 (6.6–7.4;n=17636) | 10.6 (9.4–12.0;n=2285) |
| Hepatozoon | 26.8 (21.2–33.2;n=209) | 10.7 (7.7–14.8;n=298) | 9.9 (8.4–11.6;n=1334) | 5.7 (2.8–11.4;n=122) |  |
| Leishmania | 8.7 (7.6–10.0;n=2065) | 10.1 (7.7–13.1;n=476) | 14.0 (12.4–15.7;n=1661) | 7.3 (6.7–8.0;n=6216) |  |
| Piroplasma | 0.0 (0.0–0.0;n=50) | 4.1 (1.9–8.7;n=146) | 0.8 (0.2–2.8;n=259) | 6.7 (3.8–11.6;n=164) |  |
| Plagiorchis | 4.3 (2.2–8.2;n=188) |  |  |  |  |
| Protozoa | 57.8 (45.6–69.1;n=64) |  | 31.3 (26.0–37.1;n=262) | 38.6 (27.1–51.6;n=57) |  |
| Sarcocystis | 7.1 (5.5–9.1;n=799) | 0.0 (0.0–0.0;n=152) | 1.5 (0.6–3.9;n=262) | 0.8 (0.6–1.1;n=6550) | 0.5 (0.1–2.6;n=217) |
| Thelazia | 3.1 (0.8–10.5;n=65) |  |  | 8.1 (4.0–15.9;n=86) |  |
| Toxoplasma | 30.2 (29.6–30.7;n=24481) | 16.8 (15.9–17.8;n=5878) | 37.3 (36.4–38.2;n=10674) | 18.8 (18.4–19.2;n=33513) | 5.7 (4.8–6.8;n=2020) |
| Tritrichomonas | 1.8 (0.5–6.2;n=113) |  | 2.1 (0.7–5.9;n=146) | 2.1 (1.7–2.7;n=3162) | 0.8 (0.1–4.2;n=132) |
| Trypanosoma | 9.5 (5.8–15.1;n=158) | 25.8 (16.6–37.9;n=62) | 34.5 (27.8–41.9;n=171) | 57.6 (44.9–69.4;n=59) |  |
| d) Viral | | | | | |
| HPAI |  |  | 100.0 (51.0–100.0;n=4) |  |  |
| CDV |  |  | 0.9 (0.2–4.9;n=112) |  |  |
| Cowpox |  |  | 8.2 (5.2–12.8;n=207) |  |  |
| FIV | 7.7 (6.5–9.0;n=1696) | 4.2 (2.9–6.0;n=671) | 9.5 (7.7–11.6;n=872) | 20.0 (17.6–22.6;n=990) |  |
| FeCOC | 18.3 (15.3–21.7;n=553) |  |  |  |  |
| FeLV | 4.9 (3.9–6.0;n=1646) | 2.0 (1.1–3.6;n=505) | 3.7 (2.4–5.6;n=544) | 16.7 (14.5–19.1;n=990) |  |
| Hantavirus |  |  | 4.0 (2.0–7.7;n=200) | 16.7 (11.2–24.1;n=126) |  |
| Rabies | 6.7 (4.3–10.2;n=283) |  |  |  |  |
| Raccoonpox |  |  | 100.0 (20.7–100.0;n=1) |  |  |
| Rotaviruses |  | 12.5 (5.0–28.1;n=32) | 12.5 (6.7–22.1;n=72) |  |  |
| SARS-CoV-2 |  |  |  | 0.7 (0.3–1.4;n=920) |  |
