# Supplementary material for: Outdoor roaming of owned cats elevates risk of zoonotic pathogen exposure: A global synthesis
Source: PLoS Pathog. 2026 Apr 20;22(4):e1014160. doi: 10.1371/journal.ppat.1014160 (PMC13128103; doi:10.1371/journal.ppat.1014160)
Supplement: S2 Table — For each parameter, Gelman–Rubin statistics are reported as the point estimate of R^ (R^) and its upper confidence limit (R^ upper), along with effective sample size (ESS) and Geweke diagnostics. (DOCX) [file ppat.1014160.s002.docx]

**S2 Table.** Convergence diagnostics for fixed-effect parameters from all Bayesian models. All models used indoor cats (Life.Indoor) and molecular methods as the reference level for lifestyle and detection, respectively. For each fixed-effect parameter, Gelman–Rubin diagnostics are reported as the point estimate of R̂ and its upper confidence limit (R̂ upper), along with effective sample size (ESS) and Geweke abs max diagnostics (Z).

| Model | Parameter | R̂ | R̂ upper | ESS | Geweke |
| --- | --- | --- | --- | --- | --- |
| Ancyclostoma ~ Life.Cat + (1\| Ref) + (1\|Country) | (Intercept) | 1.000 | 1.001 | 8118.23 | 1.072 |
|  | Life.Outdoor | 1.000 | 1.000 | 9178.56 | 1.883 |
|  | Life.Feral | 1.000 | 1.001 | 8005.46 | 0.597 |
| Bartonella ~ Life.Cat + method + (1\| Ref) + (1\|Country) | (Intercept) | 1.000 | 1.000 | 8212.57 | 1.327 |
|  | Life.Outdoor | 1.000 | 1.000 | 6955.63 | 0.761 |
|  | Life.Feral | 1.000 | 1.001 | 90009 | 0.850 |
|  | methodCulture | 1.000 | 1.000 | 12465.99 | 1.541 |
|  | methodSerology | 1.000 | 1.001 | 12540.42 | 0.941 |
| Cryptosporidium ~ Life.Cat + (1\| Ref) + (1\|Country) + (1\|method) | (Intercept) | 1.002 | 1.003 | 9249.12 | 2.247 |
|  | Life.Outdoor | 1.000 | 1.000 | 7727.97 | 1.832 |
|  | Life.Feral | 1.000 | 1.001 | 8794.87 | 0.938 |
| Giardia ~ Life.Cat + (1\| Ref) + (1\|Country) + (1\|method) | (Intercept) | 1.024 | 1.024 | 8786.8 | 2.153 |
|  | Life.Outdoor | 1.000 | 1.000 | 8469.12 | 1.194 |
|  | Life.Feral | 1.000 | 1.000 | 7448.62 | 1.052 |
| Leptospirosis ~ Life.Cat + method + (1\| Ref) + (1\|Country) | (Intercept) | 1.000 | 1.000 | 8829.21 | 0.791 |
|  | Life.Outdoor | 1.000 | 1.001 | 9000 | 0.974 |
|  | Life.Feral | 1.000 | 1.000 | 9000 | 2.259 |
|  | methodSerology | 1.001 | 1.002 | 11761.15 | 1.043 |
| T. cati ~ Life.Cat + (1\| Ref) + (1\|Country) + (1\|method) | (Intercept) | 1.001 | 1.001 | 10025 | 0.695 |
|  | Life.Outdoor | 1.000 | 1.000 | 9000 | 0.561 |
|  | Life.Feral | 1.000 | 1.000 | 8761.84 | 1.380 |
| T. gondii ~ Life.Cat + method + (1\| Ref) + (1\|Country) | (Intercept) | 1.000 | 1.000 | 8777.59 | 1.592 |
|  | Life.Outdoor | 1.001 | 1.001 | 9159.37 | 1.854 |
|  | Life.Feral | 1.000 | 1.000 | 9168.47 | 0.597 |
|  | methodMicroscopy | 1.000 | 1.000 | 12181.43 | 1.519 |
|  | methodSerology | 1.000 | 1.000 | 12000.00 | 1.299 |
| All_Zoo ~ Life.Cat + method + (1\| Ref) + (1\|Country) + (1\|Pathogen) | (Intercept) | 1.000 | 1.000 | 9000 | 1.176 |
|  | Life.Outdoor | 1.000 | 1.000 | 9205.28 | 2.331 |
|  | Life.Feral | 1.000 | 1.000 | 8845.37 | 2.327 |
|  | methodCulture | 1.000 | 1.000 | 9000.00 | 0.585 |
|  | methodFecal Ag | 1.000 | 1.000 | 10062.00 | 2.080 |
|  | methodMicroscopy | 1.000 | 1.000 | 9000.00 | 1.780 |
|  | methodSerology | 1.000 | 1.000 | 9000.00 | 0.725 |
