# Supplementary material for: Outdoor roaming of owned cats elevates risk of zoonotic pathogen exposure: A global synthesis
Source: PLoS Pathog. 2026 Apr 20;22(4):e1014160. doi: 10.1371/journal.ppat.1014160 (PMC13128103; doi:10.1371/journal.ppat.1014160)
Supplement: S3 Table — All data were extracted by a single reviewer (AW) between June and December 2024. All studies listed met the predefined eligibility criteria for inclusion in the systematic review, and all extracted variables required to reproduce the analyses are provided in S1 Data. (DOCX) [file ppat.1014160.s003.docx]

**S3 Table.** Complete reference list of all studies included in Dataset S1. All data were extracted by a single reviewer (AW) between June and December 2024. All studies listed met the predefined eligibility criteria for inclusion in the systematic review, and all extracted variables required to reproduce the analyses are provided in Dataset S1.

1. Abbas I, Al-Araby M, Elmishmishy B, El-Alfy E-S. Gastrointestinal parasites of cats in Egypt: high prevalence high zoonotic risk. BMC Vet Res. 2022;18: 420. doi:10.1186/s12917-022-03520-0

2. Abdelbaset AE, Alhasan H, Salman D, Karram MH, Ellah Rushdi MA, Xuenan X, et al. Evaluation of recombinant antigens in combination and single formula for diagnosis of feline toxoplasmosis. Experimental Parasitology. 2017;172: 1–4. doi:10.1016/j.exppara.2016.11.003

3. Abu-Madi MA, Pal P, Al-Thani A, Lewis JW. Descriptive epidemiology of intestinal helminth parasites from stray cat populations in Qatar. Journal of Helminthology. 2008;82: 59–68. doi:10.1017/S0022149X07870830

4. Acke E, Whyte P, Jones BR, McGill K, Collins JD, Fanning S. Prevalence of thermophilic Campylobacter species in cats and dogs in two animal shelters in Ireland. Veterinary Record. 2006;158: 51–54. doi:10.1136/vr.158.2.51

5. Adams P, Elliot A, Algar D, Brazell R. Gastrointestinal parasites of feral cats from Christmas Island. Australian Veterinary Journal. 2008;86: 60–63. doi:10.1111/j.1751-0813.2007.00246.x

6. Adhikari RB, Dhakal MA, Ale PB, Regmi GR, Ghimire TR. Survey on the prevalence of intestinal parasites in domestic cats (Felis catus Linnaeus, 1758) in central Nepal. Vet Med Sci. 2022;9: 559–571. doi:10.1002/vms3.999

7. Adriaanse K, Firestone SM, Lynch M, Rendall AR, Sutherland DR, Hufschmid J, et al. Comparison of the modified agglutination test and real-time PCR for detection of Toxoplasma gondii exposure in feral cats from Phillip Island, Australia, and risk factors associated with infection. Int J Parasitol Parasites Wildl. 2020;12: 126–133. doi:10.1016/j.ijppaw.2020.05.006

8. Advincula JK dela C, Iewida SYP, Cabanacan-Salibay C. Serologic detection of *Toxoplasma gondii* infection in stray and household cats and its hematologic evaluation. Scientia Medica. 2010;20: 76–82.

9. Afonso E, Thulliez P, Gilot-Fromont E. Local meteorological conditions, dynamics of seroconversion to Toxoplasma gondii in cats (Felis catus) and oocyst burden in a rural environment. Epidemiol Infect. 2010;138: 1105–1113. doi:10.1017/S0950268809991270

10. Afonso E, Germain E, Poulle M-L, Ruette S, Devillard S, Say L, et al. Environmental determinants of spatial and temporal variations in the transmission of *Toxoplasma gondii* in its definitive hosts. International Journal for Parasitology: Parasites and Wildlife. 2013;2: 278–285. doi:10.1016/j.ijppaw.2013.09.006

11. Agunloye CA, Nash AS. Investigation of possible leptospiral infection in cats in Scotland. Journal of Small Animal Practice. 1996;37: 126–129. doi:10.1111/j.1748-5827.1996.tb02360.x

12. Ahmad N, Ahmed H, Irum S, Qayyum. Seroprevalence of IgG and IgM antibodies and associated risk factors for toxoplasmosis in cats and dogs from subtropical arid parts of Pakistan. Tropical Biomedicine. 2014;31: 777–784.

13. Ahmadi A, Oryan A, Alidadi S. Parasites of Stray Cats in Iran: A Parasitological and Histopathological Study. Acta Parasit. 2024;69: 664–674. doi:10.1007/s11686-024-00800-7

14. Ahn K-S, Ahn A-J, Park S, Sohn W-M, Shim J, Shin S-S. Excretion of Toxoplasma gondii oocysts from Feral Cats in Korea. Korean J Parasitol. 2019;57: 665–670. doi:10.3347/kjp.2019.57.6.665

15. Akhtardanesh B, Ziaali N, Sharifi H, Rezaei S. Feline immunodeficiency virus, feline leukemia virus and Toxoplasma gondii in stray and household cats in Kerman-Iran: seroprevalence and correlation with clinical and laboratory findings. Res Vet Sci. 2010;89: 306–310. doi:10.1016/j.rvsc.2010.03.015

16. Al-Kappany YM, Lappin MR, Kwok OCH, Abu-Elwafa SA, Hilali M, Dubey JP. Seroprevalence of Toxoplasma gondii and concurrent Bartonella spp., feline immunodeficiency virus, feline leukemia virus, and Dirofilaria immitis infections in Egyptian cats. J Parasitol. 2011;97: 256–258. doi:10.1645/GE-2654.1

17. Al-Kappany YM, Rajendran C, Ferreira LR, Kwok OCH, Abu-Elwafa SA, Hilali M, et al. High Prevalence of Toxoplasmosis in Cats from Egypt: Isolation of Viable Toxoplasma gondii, Tissue Distribution, and Isolate Designation. Journal of Parasitology. 2010;96: 1115–1118. doi:10.1645/GE-2554.1

18. Al-Majali AM. Seroprevalence of and risk factors for Bartonella henselae and Bartonella quintana infections among pet cats in Jordan. Preventive Veterinary Medicine. 2004;64: 63–71. doi:10.1016/j.prevetmed.2004.03.008

19. Al-Mohammed H. Seroprevalence of Toxoplasma gondii Infection in Cats, Dogs and Ruminant Animals in Al-Ahsa Area in Saudi Arabia. Research Journal of Medical Sciences. 2011;5. doi:10.3923/rjmsci.2011.190.192

20. Al-Ramahi H M, Hamza R, Abdulla M. Seroprevalence study of Toxoplasmosis in domestic animals in Mid-Euphrates region-Iraq. J Babylon Uni. 2010;18: 1382–1387.

21. Alani ZK, Kawan MH. Prevalence and molecular analysis of Toxocara cati in Baghdad Province. J Adv Vet Anim Res. 2024;11: 392–397. doi:10.5455/javar.2024.k788

22. Alashraf AR, Lau SF, Khairani-Bejo S, Khor KH, Ajat M, Radzi R, et al. First report of pathogenic Leptospira spp. isolated from urine and kidneys of naturally infected cats. PLOS ONE. 2020;15: e0230048. doi:10.1371/journal.pone.0230048

23. Alashraf AR, Lau SF, Khor KH, Khairani-Bejo S, Bahaman AR, Roslan MA, et al. Serological Detection of Anti-*Leptospira* Antibodies in Shelter Cats in Malaysia. Topics in Companion Animal Medicine. 2019;34: 10–13. doi:10.1053/j.tcam.2018.12.002

24. Alves AS, Milhano N, Santos-Silva M, Santos AS, Vilhena M, Sousa R de. Evidence of Bartonella spp., Rickettsia spp. and Anaplasma phagocytophilum in domestic, shelter and stray cat blood and fleas, Portugal. Clinical Microbiology and Infection. 2009;15: 1–3. doi:10.1111/j.1469-0691.2008.02636.x

25. Alves MEM, Martins FDC, Bräunig P, Pivoto FL, Sangioni LA, Vogel FSF. Molecular detection of Cryptosporidium spp. and the occurrence of intestinal parasites in fecal samples of naturally infected dogs and cats. Parasitol Res. 2018;117: 3033–3038. doi:10.1007/s00436-018-5986-4

26. Amoroso MG, Serra F, Miletti G, Cardillo L, de Martinis C, Marati L, et al. A Retrospective Study of Viral Molecular Prevalences in Cats in Southern Italy (Campania Region). Viruses. 2022;14: 2583. doi:10.3390/v14112583

27. Anderson TC, Foster GW, Forrester DJ. Hookworms of feral cats in Florida. Veterinary Parasitology. 2003;115: 19–24. doi:10.1016/S0304-4017(03)00162-6

28. André MR, Calchi AC, Furquim MEC, de Andrade I, Arantes PVC, de Melo Lopes LC, et al. Molecular Detection of Tick-Borne Agents in Cats from Southeastern and Northern Brazil. Pathogens. 2022;11: 106. doi:10.3390/pathogens11010106

29. André MR, Herrera HM, Fernandes S de J, de Sousa KCM, Gonçalves LR, Domingos IH, et al. Tick-borne agents in domesticated and stray cats from the city of Campo Grande, state of Mato Grosso do Sul, midwestern Brazil. Ticks Tick Borne Dis. 2015;6: 779–786. doi:10.1016/j.ttbdis.2015.07.004

30. Aramini JJ, Stephen C, Dubey JP, Engelstoft C, Schwantje H, Ribble CS. Potential contamination of drinking water with Toxoplasma gondii oocysts. Epidemiology & Infection. 1999;122: 305–315. doi:10.1017/S0950268899002113

31. Arbabi M, Hossein H. Gastrointestinal parasites of stray cats in Kashan, Iran. Trop Biomed. 2009;26: 16–22.

32. Arraes-Santos AI, Araújo AC, Guimarães MF, Santos JR, Pena HFJ, Gennari SM, et al. Seroprevalence of anti-Toxoplasma gondii and anti-Neospora caninum antibodies in domestic mammals from two distinct regions in the semi-arid region of Northeastern Brazil. Vet Parasitol Reg Stud Reports. 2016;5: 14–18. doi:10.1016/j.vprsr.2016.08.007

33. Arruda IF, Ramos RCF, Barbosa A da S, Abboud LC de S, dos Reis IC, Millar PR, et al. Intestinal parasites and risk factors in dogs and cats from Rio de Janeiro, Brazil. Veterinary Parasitology: Regional Studies and Reports. 2021;24: 100552. doi:10.1016/j.vprsr.2021.100552

34. Arunvipas P, Jittapalapong S, Inpankaew T, Pinyopanuwat N, Chimnoi W, Maruyama S. Seroprevalence and risk factors influenced transmission of Toxoplasma gondii in dogs and cats in dairy farms in Western Thailand. African Journal of Agricultural Research. 2013;8: 591–595.

35. Asgari Q, Mohammadpour I, Pirzad R, Kalantari M, Motazedian MH, Naderi S. Molecular and Serological Detection of Toxoplasma gondii in Stray Cats in Shiraz, South-central, Iran. Iranian Journal of Parasitology. 2018;13: 430.

36. Assarasakorn S, Veir JK, Hawley JR, Brewer MM, Morris AK, Hill AE, et al. Prevalence of Bartonella species, hemoplasmas, and Rickettsia felis DNA in blood and fleas of cats in Bangkok, Thailand. Res Vet Sci. 2012;93: 1213–1216. doi:10.1016/j.rvsc.2012.03.015

37. Asthana SP, Macpherson CNL, Weiss SH, Stephens R, Denny TN, Sharma RN, et al. Seroprevalence of Toxoplasma gondii in Pregnant Women and Cats in Grenada, West Indies. para. 2006;92: 644–645. doi:10.1645/GE-762R.1

38. Attipa C, Papasouliotis K, Solano-Gallego L, Baneth G, Nachum-Biala Y, Sarvani E, et al. Prevalence study and risk factor analysis of selected bacterial, protozoal and viral, including vector-borne, pathogens in cats from Cyprus. Parasit Vectors. 2017;10: 130. doi:10.1186/s13071-017-2063-2

39. Attipa C, Yiapanis C, Tasker S, Diakou A. Seroprevalence of Toxoplasma gondii in Cats from Cyprus. Pathogens. 2021;10: 882. doi:10.3390/pathogens10070882

40. Ayinmode AB, Oluwayelu DO, Babalola ET, Lawani MA. Serologic survey of Toxoplasma gondii antibodies in cats (Felis catus) sold at live animal markets in southwestern Nigeria. Bulg J Vet Med. 2017;20: 58–64.

41. Ayllón T, Diniz PPVP, Breitschwerdt EB, Villaescusa A, Rodríguez-Franco F, Sainz A. Vector-borne diseases in client-owned and stray cats from Madrid, Spain. Vector Borne Zoonotic Dis. 2012;12: 143–150. doi:10.1089/vbz.2011.0729

42. Azimian H, Shokrani H, Fallahi S. Molecular evaluation of Toxocara species in stray cats using loop-mediated isothermal amplification (lamp) technique as a rapid, sensitive and simple screening assay. Vet Med Sci. 2021;7: 647–653. doi:10.1002/vms3.431

43. Azizi H, Hataminejad M, Taghipour A, Norouzi M, Mirzapour A. Molecular detection and genotyping of *Toxoplasma gondii* in stray cat feces from Khorramabad, West Iran. Veterinary and Animal Science. 2024;25: 100389. doi:10.1016/j.vas.2024.100389

44. Azócar-Aedo L, Monti G, Jara R. Leptospira spp. in Domestic Cats from Different Environments: Prevalence of Antibodies and Risk Factors Associated with the Seropositivity. Animals (Basel). 2014;4: 612–626. doi:10.3390/ani4040612

45. Baker J, Barton M, Lanser J. Campylobacter species in cats and dogs in South Australia. Australian Veterinary Journal. 1999;77: 662–666. doi:10.1111/j.1751-0813.1999.tb13159.x

46. Ballash GA, Dubey JP, Kwok OCH, Shoben AB, Robison TL, Kraft TJ, et al. Seroprevalence of Toxoplasma gondii in White-Tailed Deer (Odocoileus virginianus) and Free-Roaming Cats (Felis catus) Across a Suburban to Urban Gradient in Northeastern Ohio. EcoHealth. 2015;12: 359–367. doi:10.1007/s10393-014-0975-2

47. Ballweber LR, Panuska C, Huston CL, Vasilopulos R, Pharr GT, Mackin A. Prevalence of and risk factors associated with shedding of *Cryptosporidium felis* in domestic cats of Mississippi and Alabama. Veterinary Parasitology. 2009;160: 306–310. doi:10.1016/j.vetpar.2008.11.018

48. Barradas PF, de Sousa R, Vilhena H, Oliveira AC, Luz MF, Granada S, et al. Serological and molecular evidence of Bartonella henselae in cats from Luanda city, Angola. Acta Tropica. 2019;195: 142–144. doi:10.1016/j.actatropica.2019.04.012

49. Barrios N, Gómez M, Zanelli M, Rojas-Barón L, Sepúlveda-García P, Alabí A, et al. A Molecular Survey on Neglected Gurltia paralysans and Aelurostrongylus abstrusus Infections in Domestic Cats (Felis catus) from Southern Chile. Pathogens. 2021;10: 1195. doi:10.3390/pathogens10091195

50. Barros M, Cabezón O, Dubey JP, Almería S, Ribas MP, Escobar LE, et al. Toxoplasma gondii infection in wild mustelids and cats across an urban-rural gradient. PLOS ONE. 2018;13: e0199085. doi:10.1371/journal.pone.0199085

51. Barros RS, Menezes RC, Pereira SA, Figueiredo FB, Oliveira R de VC de, Nicolau JL, et al. Feline Sporotrichosis: Coinfection with Toxoplasma gondii, Feline Immunodeficiency Virus and Feline Leukemia Virus in Cats From an Endemic Area in Brazil. 2015 [cited 4 Mar 2024]. Available: https://www.arca.fiocruz.br/handle/icict/13376

52. Barrs V, Beatty J, Wilson B, Evans N, Gowan R, Baral R, et al. Prevalence of Bartonella species, Rickettsia felis, haemoplasmas and the Ehrlichia group in the blood of cats and fleas in eastern Australia. Australian Veterinary Journal. 2010;88: 160–165. doi:10.1111/j.1751-0813.2010.00569.x

53. Barutzki D, Schaper R. Endoparasites in dogs and cats in Germany 1999 – 2002. Parasitol Res. 2003;90: S148–S150. doi:10.1007/s00436-003-0922-6

54. Bastos BF, Brener B, Gershony L, Willi L, Labarthe N, Pereira C, et al. Seroprevalence of Toxoplasma gondii and retroviral status of client-owned pet cats in Rio de Janeiro, Brazil. Rev Inst Med trop S Paulo. 2014;56: 201–203. doi:10.1590/S0036-46652014000300004

55. Bayliss DB, Steiner JM, Sucholdolski JS, Radecki SV, Brewer MM, Morris AK, et al. Serum feline pancreatic lipase immunoreactivity concentration and seroprevalences of antibodies against Toxoplasma gondii and Bartonella species in client-owned cats. J Feline Med Surg. 2009;11: 663–667. doi:10.1016/j.jfms.2009.01.006

56. Bayou K, Terefe G, Kumsa B. Gastrointestinal parasites of owned cats in three districts of Central Ethiopia: Prevalence and risk factors. Veterinary Parasitology: Regional Studies and Reports. 2024;52: 101053. doi:10.1016/j.vprsr.2024.101053

57. Becker A-C, Rohen M, Epe C, Schnieder T. Prevalence of endoparasites in stray and fostered dogs and cats in Northern Germany. Parasitol Res. 2012;111: 849–857. doi:10.1007/s00436-012-2909-7

58. Bender JB, Shulman SA, Averbeck GA, Pantlin GC, Stromberg BE. Epidemiologic features of Campylobacter infection among cats in the upper midwestern United States. Journal of the American Veterinary Medical Association. 2005;226: 544–547. doi:10.2460/javma.2005.226.544

59. Bennett AD, Gunn-Moore DA, Brewer M, Lappin MR. Prevalence of Bartonella species, haemoplasmas and Toxoplasma gondii in cats in Scotland. Journal of Feline Medicine and Surgery. 2011;13: 553–557. doi:10.1016/j.jfms.2011.03.006

60. Bergmann M, Englert T, Stuetzer B, Hawley JR, Lappin MR, Hartmann K. Risk factors of different hemoplasma species infections in cats. BMC Veterinary Research. 2017;13: 52. doi:10.1186/s12917-017-0953-3

61. Bergmann M, Hartmann K. Anaplasmose beim Hund – Infektion häufig, Krankheit selten. kleintier konkret. 2015;18: 3–7. doi:10.1055/s-0035-1558509

62. Bergmans AM, de Jong CM, van Amerongen G, Schot CS, Schouls LM. Prevalence of Bartonella species in domestic cats in The Netherlands. Journal of Clinical Microbiology. 1997;35: 2256–2261. doi:10.1128/jcm.35.9.2256-2261.1997

63. Betance L, Peda A, Conan A, Riberio J. Seroprevalence of Leptospirosis in the Feral Cat Population of St. Kitts. Journal of Animal Research and Technology. 2017; 38–47. doi:10.5147/jart.2017.0161

64. Beugnet F, Bourdeau P, Chalvet-Monfray K, Cozma V, Farkas R, Guillot J, et al. Parasites of domestic owned cats in Europe: co-infestations and risk factors. Parasit Vectors. 2014;7: 291. doi:10.1186/1756-3305-7-291

65. Bezerra JAB, Haisi A, Rocha G dos S, Lima SG, Brasil AW de L, Tomaz KLR, et al. Coinfection with Leishmania infantum and Toxoplasma gondii in Domestic Cats from a Region with a High Prevalence of Feline Immunodeficiency Virus. Microorganisms. 2024;12: 71. doi:10.3390/microorganisms12010071

66. Bjöersdorff A, Svendenius L, Owens JH, Massung RF. Feline granulocytic ehrlichiosis -a report of a new clinical entity and characterisation of the infectious agent. Journal of Small Animal Practice. 1999;40: 20–24. doi:10.1111/j.1748-5827.1999.tb03249.x

67. Bojanić K, Midwinter AC, Marshall JC, Rogers LE, Biggs PJ, Acke E. Isolation of Campylobacter spp. from Client-Owned Dogs and Cats, and Retail Raw Meat Pet Food in the Manawatu, New Zealand. Zoonoses Public Health. 2017;64: 438–449. doi:10.1111/zph.12323

68. Bolais PF, Vignoles P, Pereira PF, Keim R, Aroussi A, Ismail K, et al. Toxoplasma gondii survey in cats from two environments of the city of Rio de Janeiro, Brazil by Modified Agglutination Test on sera and filter-paper. Parasites Vectors. 2017;10: 88. doi:10.1186/s13071-017-2017-8

69. Bonelli P, Masu G, Dei Giudici S, Pintus D, Peruzzu A, Piseddu T, et al. Cystic echinococcosis in a domestic cat (Felis catus) in Italy. Parasite. 2018;25: 25. doi:10.1051/parasite/2018027

70. Borthakur SK, Mukharjee SN. Gastrointestinal Helminthes in Stray Cats (felis Catus) from Aizawl, Mizoram, India. Southeast Asian Journal of Tropical Medicine and Public Health. 2011;42: 255–8.

71. Boughattas S, Behnke J, Sharma A, Abu-Madi M. Seroprevalence of Toxoplasma gondii infection in feral cats in Qatar. BMC Vet Res. 2016;13: 26. doi:10.1186/s12917-017-0952-4

72. Bourassi E, Savidge C, Foley P, Hartwig S. Serologic and urinary survey of exposure to *Leptospira* species in a feral cat population of Prince Edward Island, Canada. J Feline Med Surg. 2021;23: 1155–1161. doi:10.1177/1098612X211001042

73. Bourgoin G, Callait-Cardinal M-P, Bouhsira E, Polack B, Bourdeau P, Roussel Ariza C, et al. Prevalence of major digestive and respiratory helminths in dogs and cats in France: results of a multicenter study. Parasites & Vectors. 2022;15: 314. doi:10.1186/s13071-022-05368-7

74. Braga ARC, Corrêa APFL, Camossi LG, Silva RC da, Langoni H, Lucheis SB. Coinfection by *Toxoplasma gondii* and *Leishmania* spp. in domestic cats (*Felis catus*) in State of Mato Grosso do Sul. Rev Soc Bras Med Trop. 2014;47: 796–797. doi:10.1590/0037-8682-0041-2014

75. Braga M do SC de O, André MR, Jusi MMG, Freschi CR, Teixeira MCA, Machado RZ. Occurrence of anti-Toxoplasma gondii and anti-Neospora caninum antibodies in cats with outdoor access in São Luís, Maranhão, Brazil. Rev Bras Parasitol Vet. 2012;21: 107–111. doi:10.1590/S1984-29612012000200007

76. Braga M do SC de O, Diniz PPV de P, André MR, de Bortoli CP, Machado RZ. Molecular characterisation of Bartonella species in cats from São Luís, state of Maranhão, north-eastern Brazil. Mem Inst Oswaldo Cruz. 2012;107: 772–777. doi:https://doi.org/10.1590/S0074-02762012000600011

77. Brasil AW de L, Parantoni RN, Feitosa TF, Vilela VLR, Alves CJ, Vasconcellos SA, et al. Anticorpos anti-Leptospira spp. em gatos do semiárido do Estado da Paraíba. Semina ciÃ^a^nc agrar. 2014;35: 3215–3220.

78. Breitschwerdt EB, Maggi RG, Sigmon B, Nicholson WL. Isolation of Bartonella quintana from a Woman and a Cat following Putative Bite Transmission. Journal of Clinical Microbiology. 2007;45: 270–272. doi:10.1128/jcm.01451-06

79. Brennan A, Hawley J, Dhand N, Boland L, Beatty JA, Lappin MR, et al. Seroprevalence and Risk Factors for Toxoplasma gondii Infection in Owned Domestic Cats in Australia. Vector-Borne and Zoonotic Diseases. 2020 [cited 4 Mar 2024]. doi:10.1089/vbz.2019.2520

80. Bresciani KDS, Gennari SM, Serrano ACM, Rodrigues AAR, Ueno T, Franco LG, et al. Antibodies to Neospora caninum and Toxoplasma gondii in domestic cats from Brazil. Parasitol Res. 2007;100: 281–285. doi:10.1007/s00436-006-0262-4

81. Briand F-X, Souchaud F, Pierre I, Beven V, Hirchaud E, Hérault F, et al. Highly Pathogenic Avian Influenza A(H5N1) Clade 2.3.4.4b Virus in Domestic Cat, France, 2022. Emerg Infect Dis. 2023;29: 1696–1698. doi:10.3201/eid2908.230188

82. Bruno F, Vitale F, La Russa F, Reale S, Späth GF, Oliveri E, et al. Retrospective Analysis of Leishmaniasis in Sicily (Italy) from 2013 to 2021: One-Health Impact and Future Control Strategies. Microorganisms. 2022;10: 1704. doi:10.3390/microorganisms10091704

83. Buddhirongawatr R, Chaichoun K, Tungsudjai S, Udonsom R, Thompson A, Mahittikorn O, et al. Seroprevalence and Phylogenetic Analysis of Toxoplasma gondii from Domestic Cats, Captive Wild Felids, Free-range Wild Felids and Rats in Certain Regions of Thailand. The Thai Journal of Veterinary Medicine. 2016;46: 209–218. doi:10.56808/2985-1130.2728

84. Burnens AP, Nicolet J. Detection of Campylobacter upsaliensis in diarrheic dogs and cats, using a selective medium with cefoperazone. American Journal of Veterinary Research. 1992;53: 48–51. doi:10.2460/ajvr.1992.53.01.48

85. Butti MJ, Gamboa MI, Terminiello JD, Franchini GR, Giorello AN, Maldonado LL, et al. *Dioctophyme renale* in a domestic cat (*Felis catus*): Renal location and nephrectomy. Veterinary Parasitology: Regional Studies and Reports. 2019;18: 100339. doi:10.1016/j.vprsr.2019.100339

86. Calvete C, Lucientes J, Castillo JA, Estrada R, Gracia MJ, Peribáñez MA, et al. Gastrointestinal helminth parasites in stray cats from the mid-Ebro Valley, Spain. Vet Parasitol. 1998;75: 235–240. doi:10.1016/s0304-4017(97)00182-9

87. Calvopiña M, Cevallos W, Atherton R, Saunders M, Small A, Kumazawa H, et al. High Prevalence of the Liver Fluke Amphimerus sp. in Domestic Cats and Dogs in an Area for Human Amphimeriasis in Ecuador. PLOS Neglected Tropical Diseases. 2015;9: e0003526. doi:10.1371/journal.pntd.0003526

88. Camprigher VM, Matos AMRN, Ferreira FP, Batina PN, Costa SC, Navarro IT, et al. Ocorrência de anticorpos anti-*Leishmania* spp. em felinos em área endêmica do estado de São Paulo. Arq Bras Med Vet Zootec. 2019;71: 439–446. doi:https://doi.org/10.1590/1678-4162-10291

89. Can H, Köseoğlu AE, Erkunt Alak S, Güvendi M, Ün C, Karakavuk M, et al. Molecular prevalence and subtyping of Blastocystis sp. isolates in stray cats of İzmir, Turkey: First report of “ST4 allele 42” in cats. Pol J Vet Sci. 2021;24: 217–223. doi:10.24425/pjvs.2021.137656

90. Can H, Döşkaya M, Ajzenberg D, Özdemir HG, Caner A, İz SG, et al. Genetic Characterization of Toxoplasma gondii Isolates and Toxoplasmosis Seroprevalence in Stray Cats of İzmir, Turkey. PLOS ONE. 2014;9: e104930. doi:10.1371/journal.pone.0104930

91. Candela MG, Fanelli A, Carvalho J, Serrano E, Domenech G, Alonso F, et al. Urban landscape and infection risk in free-roaming cats. Zoonoses and Public Health. 2022;69: 295–311. doi:10.1111/zph.12919

92. Cantó GJ, Guerrero RI, Olvera-Ramírez AM, Milián F, Mosqueda J, Aguilar-Tipacamú G. Prevalence of Fleas and Gastrointestinal Parasites in Free-Roaming Cats in Central Mexico. PLOS ONE. 2013;8: e60744. doi:10.1371/journal.pone.0060744

93. Carabin H, Balolong E, Joseph L, McGarvey ST, Johansen MV, Fernandez T, et al. Estimating sensitivity and specificity of a faecal examination method for *Schistosoma japonicum* infection in cats, dogs, water buffaloes, pigs, and rats in Western Samar and Sorsogon Provinces, The Philippines. International Journal for Parasitology. 2005;35: 1517–1524. doi:10.1016/j.ijpara.2005.06.010

94. Cardia DFF, Camossi LG, Neto L da S, Langoni H, Bresciani KDS. Prevalence of *Toxoplasma gondii* and *Leishmania* spp. infection in cats from Brazil. Veterinary Parasitology. 2013;197: 634–637. doi:10.1016/j.vetpar.2013.07.017

95. Cardillo N, Rosa A, Sommerfelt I. Estudio preliminar sobre los distintos estadios de Toxocara cati en gatos. Parasitología latinoamericana. 2008;63: 72–75. doi:10.4067/S0717-77122008000100013

96. Cardoso CB, das Neves JH, Amarante AFT. *Lagochilascaris minor* (Nematoda, Ascarididae) in a domestic cat in a coastal city of the state of São Paulo. Veterinary Parasitology: Regional Studies and Reports. 2020;19: 100372. doi:10.1016/j.vprsr.2020.100372

97. Case JB, Chomel B, Nicholson W, Foley JE. Serological survey of vector-borne zoonotic pathogens in pet cats and cats from animal shelters and feral colonies. Journal of Feline Medicine and Surgery. 2006;8: 111–117. doi:10.1016/j.jfms.2005.10.004

98. Castro O, Valledor S, Crampet A, Casáa G. Contribution to the knowledge of metazoan parasites of domestic cat in the department of Montevideo, Uruguay. Veterinaria (Montevideo). 2013;49: 28–37.

99. Castro O, Venzal JM, Félix ML. Two new records of helminth parasites of domestic cat from Uruguay: *Alaria alata* (Goeze, 1782) (Digenea, Diplostomidae) and *Lagochilascaris major* Leiper, 1910 (Nematoda, Ascarididae). Veterinary Parasitology. 2009;160: 344–347. doi:10.1016/j.vetpar.2008.11.019

100. Cavalcante GT, Aguiar DM, Chiebao D, Dubey JP, Ruiz VLA, Dias RA, et al. Seroprevalence of Toxoplasma gondii Antibodies in Cats and Pigs From Rural Western Amazon, Brazil. para. 2006;92: 863–864. doi:10.1645/GE-830R.1

101. Cavalera MA, Schnyder M, Gueldner EK, Furlanello T, Iatta R, Brianti E, et al. Serological survey and risk factors of Aelurostrongylus abstrusus infection among owned cats in Italy. Parasitol Res. 2019;118: 2377–2382. doi:10.1007/s00436-019-06373-z

102. Cerro L, Rubio A, Pinedo R, Mendes-de-Almeida F, Brener B, Labarthe N. Seroprevalence of Toxoplasma gondii in cats (Felis catus, Linnaeus 1758) living in Lima, Peru. Rev Bras Parasitol Vet. 2014;23: 90–93. doi:10.1590/S1984-29612014013

103. Chan K-W, Hsu Y-H, Hu W-L, Pan M-J, Lai J-M, Huang K-C, et al. Serological and PCR detection of feline leptospira in southern Taiwan. Vector Borne Zoonotic Dis. 2014;14: 118–123. doi:10.1089/vbz.2013.1324

104. Chandra S, Forsyth M, Lawrence AL, Emery D, Šlapeta J. Cat fleas (*Ctenocephalides felis*) from cats and dogs in New Zealand: Molecular characterisation, presence of *Rickettsia felis* and *Bartonella clarridgeiae* and comparison with Australia. Veterinary Parasitology. 2017;234: 25–30. doi:10.1016/j.vetpar.2016.12.017

105. Chang C-C, Lee C-C, Maruyama S, Lin J-W, Pan M-J. Cat-scratch disease in veterinary-associated populations and in its cat reservoir in Taiwan. Veterinary Research. 2006;37: 565–577. doi:10.1051/vetres:2006019

106. Chomel BB, Abbott RC, Kasten RW, Floyd-Hawkins KA, Kass PH, Glaser CA, et al. Bartonella henselae prevalence in domestic cats in California: risk factors and association between bacteremia and antibody titers. J Clin Microbiol. 1995;33: 2445–2450. doi:10.1128/jcm.33.9.2445-2450.1995

107. Chomel BB, Carlos ET, Kasten RW, Yamamoto K, Chang CC, Carlos RS, et al. Bartonella henselae and Bartonella clarridgeiae infection in domestic cats from The Philippines. Am J Trop Med Hyg. 1999;60: 593–597. doi:10.4269/ajtmh.1999.60.593

108. Chomel BB, Jay MT, Smith CR, Kass PH, Pyan CP, Barrett LR. Serological surveillance of plague in dogs and cats, California, 1979–1991. Comparative Immunology, Microbiology and Infectious Diseases. 1994;17: 111–123. doi:10.1016/0147-9571(94)90036-1

109. Chong C-K, Jeong W, Kim H-Y, An D-J, Jeoung H-Y, Ryu J-E, et al. Development and Clinical Evaluation of a Rapid Serodiagnostic Test for Toxoplasmosis of Cats Using Recombinant SAG1 Antigen. Korean J Parasitol. 2011;49: 207–212. doi:10.3347/kjp.2011.49.3.207

110. Clifford DL, Mazet JAK, Dubovi EJ, Garcelon DK, Coonan TJ, Conrad PA, et al. Pathogen exposure in endangered island fox (Urocyon littoralis) populations: Implications for conservation management. Biol Conserv. 2006;131: 230–243. doi:10.1016/j.biocon.2006.04.029

111. Coelho WMD, Amarante AFT do, Apolinário J de C, Coelho NMD, Bresciani KDS. Occurrence of Ancylostoma in dogs, cats and public places from Andradina city, São Paulo state, Brazil. Rev Inst Med trop S Paulo. 2011;53: 181–184. doi:10.1590/S0036-46652011000400001

112. Coelho WMD, do Amarante AFT, Apolinário J de C, Coelho NMD, de Lima VMF, Perri SHV, et al. Seroepidemiology of Toxoplasma gondii, Neospora caninum, and Leishmania spp. infections and risk factors for cats from Brazil. Parasitol Res. 2011;109: 1009–1013. doi:10.1007/s00436-011-2461-x

113. Coelho WMD, do Amarante AFT, de Soutello RVG, Meireles MV, Bresciani KDS. [Occurrence of gastrointestinal parasites in fecal samples of cats in Andradina City, São Paulo]. Rev Bras Parasitol Vet. 2009;18: 46–49. doi:10.4322/rbpv.01802010

114. Colella V, Nguyen VL, Tan DY, Lu N, Fang F, Zhijuan Y, et al. Zoonotic Vectorborne Pathogens and Ectoparasites of Dogs and Cats in Eastern and Southeast Asia. Emerg Infect Dis. 2020;26: 1221–1233. doi:10.3201/eid2606.191832

115. Coman BJ, Jones EH, Driesen MA. Helminth parasites and arthropods of feral cats. Aust Vet J. 1981;57: 324–327. doi:10.1111/j.1751-0813.1981.tb05837.x

116. Cong W, Elsheikha HM, Zhou N, Peng P, Qin S-Y, Meng Q-F, et al. Prevalence of antibodies against Toxoplasma gondii in pets and their owners in Shandong province, Eastern China. BMC Infect Dis. 2018;18: 430. doi:10.1186/s12879-018-3307-2

117. Cong W, Meng Q-F, Blaga R, Villena I, Zhu X-Q, Qian A-D. Toxoplasma gondii, Dirofilaria immitis, feline immunodeficiency virus (FIV), and feline leukemia virus (FeLV) infections in stray and pet cats (Felis catus) in northwest China: co-infections and risk factors. Parasitol Res. 2016;115: 217–223. doi:10.1007/s00436-015-4738-y

118. Cooper A, Goullet M, Mitchell J, Ketheesan N, Govan B. Serological evidence of Coxiella burnetii exposure in native marsupials and introduced animals in Queensland, Australia. Epidemiology & Infection. 2012;140: 1304–1308. doi:10.1017/S0950268811001828

119. Côrtes V de A, Paim GV, Alencar Filho RA de. Infestação por ancilostomídeos e toxocarídeos em cães e gatos apreendidos em vias públicas, São Paulo (Brasil). Rev Saúde Pública. 1988;22: 341–343. doi:https://doi.org/10.1590/S0034-89101988000400010

120. Costa DGC, Marvulo MFV, Silva JSA, Santana SC, Magalhães FJR, Filho CDFL, et al. Seroprevalence of Toxoplasma gondii in Domestic and Wild Animals From the Fernando de Noronha, Brazil. Journal of Parasitology. 2012;98: 679–680. doi:10.1645/GE-2910.1

121. Crissiuma A, Favacho A, Gershony L, Mendes-de-Almeida F, Gomes R, Mares-Guia A, et al. Prevalence of Bartonella species DNA and antibodies in cats (Felis catus) submitted to a spay/neuter program in Rio de Janeiro, Brazil. J Feline Med Surg. 2011;13: 149–151. doi:10.1016/j.jfms.2010.08.010

122. Cruz MDA, Ullmann LS, Montaño PY, Hoffmann JL, Langoni H, Biondo AW. Seroprevalence of Toxoplasma gondii infection in cats from Curitiba, Paraná, Brazil. Rev Bras Parasitol Vet. 2011;20: 256–258. doi:10.1590/S1984-29612011000300016

123. Cyr J, Turcotte M-È, Desrosiers A, Bélanger D, Harel J, Tremblay D, et al. Prevalence of Coxiella burnetii seropositivity and shedding in farm, pet and feral cats and associated risk factors in farm cats in Quebec, Canada. Epidemiology & Infection. 2021;149: e57. doi:10.1017/S0950268821000364

124. Dabritz HA, Gardner IA, Miller MA, Lappin MR, Atwill ER, Packham AE, et al. Evaluation of two Toxoplasma gondii serologic tests used in a serosurvey of domestic cats in California. J Parasitol. 2007;93: 806–816. doi:10.1645/GE-996R.1

125. Darabi E, Kia EB, Mohebali M, Mobedi I, Zahabiun F, Zarei Z, et al. Gastrointestinal Helminthic Parasites of Stray Cats (Felis catus) in Northwest Iran. Iran J Parasitol. 2021;16: 418–425. doi:10.18502/ijpa.v16i3.7095

126. Darabus G, Hotea I, Oprescu I, Morariu S, Brudiu I, Olariu RT. Toxoplasmosis seroprevalence in cats and sheep from Western Romania. Rev Med Vet. 2011;162: 316–320.

127. Dashti A, Santín M, Cano L, de Lucio A, Bailo B, de Mingo MH, et al. Occurrence and genetic diversity of Enterocytozoon bieneusi (Microsporidia) in owned and sheltered dogs and cats in Northern Spain. Parasitol Res. 2019;118: 2979–2987. doi:10.1007/s00436-019-06428-1

128. Davis AA, Lepczyk CA, Haman KH, Morden CW, Crow SE, Jensen N, et al. Toxoplasma gondii Detection in Fecal Samples from Domestic Cats (Felis catus) in Hawai‘i1. pasc. 2018;72: 501–511. doi:10.2984/72.4.9

129. De Craeye S, Francart A, Chabauty J, De Vriendt V, Van Gucht S, Leroux I, et al. Prevalence of Toxoplasma gondii infection in Belgian house cats. Vet Parasitol. 2008;157: 128–132. doi:10.1016/j.vetpar.2008.07.001

130. de Waal T, Aungier S, Lawlor A, Goddu T, Jones M, Szlosek D. Retrospective Survey of Dog and Cat Endoparasites in Ireland: Antigen Detection. Animals. 2023;13: 137. doi:10.3390/ani13010137

131. DeFeo ML, Dubey JP, Mather TN, Iii RCR. Epidemiologic investigation of seroprevalence of antibodies to Toxoplasma gondii in cats and rodents. American Journal of Veterinary Research. 2002;63: 1714–1717. doi:10.2460/ajvr.2002.63.1714

132. Deksne G, Petrusēviča A, Kirjušina M. Seroprevalence and Factors Associated with Toxoplasma gondii Infection in Domestic Cats from Urban Areas in Latvia. para. 2013;99: 48–50. doi:10.1645/GE-3254.1

133. Deplazes P, Eckert J. Diagnosis of the Echinococcus multilocularis infection in final hosts. Appl Parasitol. 1996;37: 245–252.

134. Derakhshan M, Mousavi M. Serological survey of antibodies to Toxoplasma gondii in cats, goats, and sheep in Kerman, Iran. Comp Clin Pathol. 2014;23: 267–268. doi:10.1007/s00580-012-1605-4

135. Desvars A, Naze F, Benneveau A, Cardinale E, Michault A. Endemicity of leptospirosis in domestic and wild animal species from Reunion Island (Indian Ocean). Epidemiol Infect. 2013;141: 1154–1165. doi:10.1017/S0950268812002075

136. Di Cesare A, Veronesi F, Grillotti E, Manzocchi S, Perrucci S, Beraldo P, et al. Respiratory nematodes in cat populations of Italy. Parasitol Res. 2015;114: 4463–4469. doi:10.1007/s00436-015-4687-5

137. Diakou A, Di Cesare A, Accettura PM, Barros L, Iorio R, Paoletti B, et al. Intestinal parasites and vector-borne pathogens in stray and free-roaming cats living in continental and insular Greece. PLoS Negl Trop Dis. 2017;11: e0005335. doi:10.1371/journal.pntd.0005335

138. Diakou A, Sofroniou D, Di Cesare A, Kokkinos P, Traversa D. Occurrence and zoonotic potential of endoparasites in cats of Cyprus and a new distribution area for Troglostrongylus brevior. Parasitol Res. 2017;116: 3429–3435. doi:10.1007/s00436-017-5651-3

139. Díaz-Regañón D, Villaescusa A, Ayllón T, Rodríguez-Franco F, García-Sancho M, Agulla B, et al. Epidemiological study of hemotropic mycoplasmas (hemoplasmas) in cats from central Spain. Parasites & Vectors. 2018;11: 140. doi:10.1186/s13071-018-2740-9

140. Dickeson D, Love D. A serological survey of dogs, cats and horses in south-eastern Australia for leptospiral antibodies. Australian Veterinary Journal. 1993;70: 389–390. doi:10.1111/j.1751-0813.1993.tb00823.x

141. do Prado CM, Razzolini E, Santacruz G, Ojeda L, Geraldo MR, Segovia N, et al. First Cases of Feline Sporotrichosis Caused by Sporothrix brasiliensis in Paraguay. J Fungi (Basel). 2023;9: 972. doi:10.3390/jof9100972

142. Dobly A, Cochez C, Goossens E, De Bosschere H, Hansen P, Roels S, et al. Sero-epidemiological study of the presence of hantaviruses in domestic dogs and cats from Belgium. Research in Veterinary Science. 2012;92: 221–224. doi:10.1016/j.rvsc.2011.02.003

143. Dorny P, Speybroeck N, Verstraete S, Baeke M, De Becker A, Berkvens D, et al. Serological survey Toxoplasma gondii of a on feline immunodeficiency virus and feine leukaemia virus in urban stray cats in Belgium. Veterinary Record. 2002;151: 626–629. doi:10.1136/vr.151.21.626

144. Dorsch R, Ojeda J, Salgado M, Monti G, Collado B, Tomckowiack C, et al. Cats shedding pathogenic Leptospira spp.—An underestimated zoonotic risk? PLOS ONE. 2020;15: e0239991. doi:10.1371/journal.pone.0239991

145. Dos Santos LF, Guimarães MF, de Souza GO, da Silva IWG, Santos JR, Azevedo SS, et al. Seroepidemiological survey on Leptospira spp. infection in wild and domestic mammals in two distinct areas of the semi-arid region of northeastern Brazil. Trop Anim Health Prod. 2017;49: 1715–1722. doi:10.1007/s11250-017-1382-9

146. Duarte A, Castro I, Pereira da Fonseca IM, Almeida V, Madeira de Carvalho LM, Meireles J, et al. Survey of infectious and parasitic diseases in stray cats at the Lisbon Metropolitan Area, Portugal. J Feline Med Surg. 2010;12: 441–446. doi:10.1016/j.jfms.2009.11.003

147. Dubey JP, Bhatia CR, Lappin MR, Ferreira LR, Thorn A, Kwok OCH. Seroprevalence of Toxoplasma gondii and Bartonella spp. Antibodies in Cats from Pennsylvania. para. 2009;95: 578–580. doi:10.1645/GE-1933.1

148. Dubey JP, Darrington C, Tiao N, Ferreira LR, Choudhary S, Molla B, et al. Isolation of Viable Toxoplasma gondii from Tissues and Feces of Cats from Addis Ababa, Ethiopia. Journal of Parasitology. 2013;99: 56–58. doi:10.1645/GE-3229.1

149. Dubey JP, Lappin MR, Kwok OCH, Mofya S, Chikweto A, Baffa A, et al. Seroprevalence of Toxoplasma gondii and Concurrent Bartonella Spp., Feline Immunodeficiency Virus, and Feline Leukemia Virus Infections in Cats from Grenada, West Indies. para. 2009;95: 1129–1133. doi:10.1645/GE-2114.1

150. Dubey JP, López-Torres HY, Sundar N, Velmurugan GV, Ajzenberg D, Kwok OCH, et al. Mouse-virulent Toxoplasma gondii isolated from feral cats on Mona Island, Puerto Rico. J Parasitol. 2007;93: 1365–1369. doi:10.1645/GE-1409.1

151. Dubey JP, Moura L, Majumdar D, Sundar N, Velmurugan GV, Kwok OCH, et al. Isolation and characterization of viable Toxoplasma gondii isolates revealed possible high frequency of mixed infection in feral cats (Felis domesticus) from St Kitts, West Indies. Parasitology. 2009;136: 589–594. doi:10.1017/S0031182009006015

152. Dubey JP, Navarro IT, Sreekumar C, Dahl E, Freire RL, Kawabata HH, et al. ​​​​Toxoplasma gondii infections in cats from Paraná, Brazil: Seroprevalence, tissue distribution, and biologic and genetic characterization of isolates. para. 2004;90: 721–726. doi:10.1645/GE-382R

153. Dubey JP, Pas A, Rajendran C, Kwok OCH, Ferreira LR, Martins J, et al. Toxoplasmosis in Sand cats (*Felis margarita*) and other animals in the Breeding Centre for Endangered Arabian Wildlife in the United Arab Emirates and Al Wabra Wildlife Preservation, the State of Qatar. Veterinary Parasitology. 2010;172: 195–203. doi:10.1016/j.vetpar.2010.05.013

154. Dubey JP, Saville WJA, Stanek JF, Reed SM. Prevalence of Toxoplasma gondii Antibodies in Domestic Cats from Rural Ohio. The Journal of Parasitology. 2002;88: 802–803. doi:10.2307/3285366

155. Dubey JP, Su C, Cortés JA, Sundar N, Gomez-Marin JE, Polo LJ, et al. Prevalence of *Toxoplasma gondii* in cats from Colombia, South America and genetic characterization of *T. gondii* isolates. Veterinary Parasitology. 2006;141: 42–47. doi:10.1016/j.vetpar.2006.04.037

156. Dubey JP, Weigel RM, Siegel AM, Thulliez P, Kitron UD, Mitchell MA, et al. Sources and reservoirs of Toxoplasma gondii infection on 47 swine farms in Illinois. J Parasitol. 1995;81: 723–729.

157. Duda A, Stenzel DJ, Boreham PFL. Detection of *Blastocystis* sp. in domestic dogs and cats. Veterinary Parasitology. 1998;76: 9–17. doi:10.1016/S0304-4017(97)00224-0

158. Dumitrache MO, Györke A, Mircean M, Benea M, Mircean V. Ocular thelaziosis due Thelazia callipaeda (Spirurida: Thelaziidae) in Romania: first report in domestic cat and new geographical records of canine cases. Parasitol Res. 2018;117: 4037–4042. doi:10.1007/s00436-018-6122-1

159. Dybing NA, Jacobson C, Irwin P, Algar D, Adams PJ. Challenging the dogma of the ‘Island Syndrome’: a study of helminth parasites of feral cats and black rats on Christmas Island. Australasian Journal of Environmental Management. 2018;25: 99–118. doi:10.1080/14486563.2017.1417165

160. Dybing NA, Jacobson C, Irwin P, Algar D, Adams PJ. Leptospira Species in Feral Cats and Black Rats from Western Australia and Christmas Island. Vector Borne Zoonotic Dis. 2017;17: 319–324. doi:10.1089/vbz.2016.1992

161. Eberhardt JM, Neal K, Shackelford T, Lappin MR. Prevalence of selected infectious disease agents in cats from Arizona. Journal of Feline Medicine and Surgery. 2006;8: 164–168. doi:10.1016/j.jfms.2005.12.002

162. Echeverry DM, Giraldo MI, Castaño JC. Prevalence of intestinal helminths in cats in Quindío, Colombia. Biomédica. 2012;32: 430–6. doi:10.7705/biomedica.v32i3.439

163. Eidson M, Tierney LA, Rollag OJ, Becker T, Brown T, Hull HF. Feline plague in New Mexico: risk factors and transmission to humans. Am J Public Health. 1988;78: 1333–1335.

164. El-Azazy OME, Abdou N-EMI, Khalil AI, Al-Batel MK, Majeed QAH, Henedi AA-R, et al. Potential Zoonotic Trematodes Recovered in Stray Cats from Kuwait Municipality, Kuwait. Korean J Parasito. 2015;53: 279–287. doi:10.3347/kjp.2015.53.3.279

165. El-Seify MA, Aggour MG, Sultan K, Marey NM. Gastrointestinal helminths of stray cats in Alexandria, Egypt: A fecal examination survey study. Vet Parasitol Reg Stud Reports. 2017;8: 104–106. doi:10.1016/j.vprsr.2017.03.003

166. Elmahallawy EK, Gareh A, Abu-Okail A, Köster PC, Dashti A, Asseri J, et al. Molecular characteristics and zoonotic potential of enteric protists in domestic dogs and cats in Egypt. Front Vet Sci. 2023;10. doi:10.3389/fvets.2023.1229151

167. Enemark HL, Starostka TP, Larsen B, Takeuchi-Storm N, Thamsborg SM. Giardia and Cryptosporidium infections in Danish cats: risk factors and zoonotic potential. Parasitol Res. 2020;119: 2275–2286. doi:10.1007/s00436-020-06715-2

168. Enes JE, Wages AJ, Malone JB, Tesana S. Prevalence Of Opisthorchis Viverrini Infection In The Canine And Feline Hosts In Three Villages, Khon Kaen Province, Northeastern Thailand. Southeast Asian J Trop Med Public Health. 2010;41: 36–42.

169. Erkılıç E, Mor N, Kırmızıgül A, Beyhan YE, Babür C. The seroprevalence of Toxoplasma gondii in cats from the Kars region, Turkey. Israel Journal of Veterinary Medicine. 2016;71: 31–35.

170. Esmaeilzadeh M, Shamsfard M, Kazemi M, Khalafi S, Altome S. Prevalence of Protozoa and Gastrointestinal Helminthes in Stray Cats in Zanjan Province, North-West of Iran. Iranian Journal of Parasitology. 2009;4: 71–75.

171. Esteves F, Aguiar D, Rosado J, Costa ML, de Sousa B, Antunes F, et al. Toxoplasma gondii prevalence in cats from Lisbon and in pigs from centre and south of Portugal. Vet Parasitol. 2014;200: 8–12. doi:10.1016/j.vetpar.2013.12.017

172. Etter E, Neves L, Tagwireyi WM. Seroprevalence and associated risk factors of Toxoplasma gondii infection in domestic animals in southeastern South Africa. Onderstepoort Journal of Veterinary Research. 2019;86: 1–6. doi:10.4102/ojvr.v86i1.1688

173. Fa-ngoen C, Kaewmongkol G, Inthong N, Tanganuchitcharnchai A, Abdad MY, Siengsanan-Lamont J, et al. Serological detection of Rickettsia spp. and evaluation of blood parameters in pet dogs and cats from Bangkok and neighboring provinces. PLOS ONE. 2024;19: e0297373. doi:10.1371/journal.pone.0297373

174. Fabbi M, Vicari N, Tranquillo M, Pozzi C, Prati P, De Meneghi D, et al. [Prevalence of Bartonella henselae in stray and domestic cats in different Italian areas: evaluation of the potential risk of transmission of Bartonella to humans]. Parassitologia. 2004;46: 127–129.

175. Fabbi M, De Giuli L, Tranquillo M, Bragoni R, Casiraghi M, Genchi C. Prevalence of Bartonella henselae in Italian Stray Cats: Evaluation of Serology To Assess the Risk of Transmission of Bartonella to Humans. J Clin Microbiol. 2004;42: 264–268. doi:10.1128/JCM.42.1.264-268.2004

176. Fagundes-Moreira R, Silveira E, Baggio-Souza V, Marques SMT, Vidor SB, Castro SM de J, et al. Comparative analysis of diagnostic methods and risk factors for Aelurostrongylus abstrusus infection in brazilian cats. Journal of Helminthology. 2023;97: e91. doi:10.1017/S0022149X23000755

177. Faraguna S, Vlahek I, Miočić KT, Andreanszky T, Pećin M. Prevalence of Intestinal Parasites in Dogs and Cats from the Kvarner Region in Croatia. Acta Veterinaria. 2023;73: 41–54. doi:10.2478/acve-2023-0004

178. Farantika R, Susanti R. The Prevalence of Alimentary Tract Worms in Domestic Cats and Stray Cats at Campus Area of Semarang State University, Central Java. Jurnal Veteriner. 2019;20: 316–323. doi:10.19087/jveteriner.2019.20.3.316

179. Feitosa TF, Vilela VLR, Dantas ES, Souto DVO, Pena HFJ, Athayde ACR, et al. Toxoplasma gondii and Neospora caninum in domestic cats from the Brazilian semi-arid: seroprevalence and risk factors. Arq Bras Med Vet Zootec. 2014;66: 1060–1066. doi:10.1590/1678-6696

180. Fernandes ALP, Alves M de M, Silva JO, Bison I, Silva A de CT, Parentoni RN, et al. Geoepidemiology, seroprevalence and factors associated with Toxoplasma gondii infection in domicilied cats from Paraíba (Brazil). Parasite. 2024;31: 25. doi:10.1051/parasite/2024017

181. Fernandes S, Brilhante-Simões P, Coutinho T, Cardoso L, Dubey JP, Lopes AP. Comparison of indirect and modified agglutination tests for detection of antibodies to Toxoplasma gondii in domestic cats. J VET Diagn Invest. 2019;31: 774–777. doi:10.1177/1040638719868753

182. Ferreira FS, Pereira-Baltasar P, Parreira R, Padre L, Vilhena M, Távora Tavira L, et al. Intestinal parasites in dogs and cats from the district of Évora, Portugal. Veterinary Parasitology. 2011;179: 242–245. doi:10.1016/j.vetpar.2011.02.003

183. Flecke LR, Dalegrave S, Mattei AS, Guterres KA, Giordani C, Silva BG, et al. Lagoquilascariasis in domestic cat (Felis catus domesticus) - case report. Arq Bras Med Vet Zootec. 2022;74: 345–350. doi:https://doi.org/10.1590/1678-4162-12418

184. Foley JE, Chomel B, Kikuchi Y, Yamamoto K, Pedersen NC. Seroprevalence of Bartonella henselae in cattery cats: association with cattery hygiene and flea infestation. Vet Q. 1998;20: 1–5. doi:10.1080/01652176.1998.9694824

185. Fournier GF da SR, Lopes MG, Marcili A, Ramirez DG, Acosta ICL, Ferreira JIG da S, et al. *Toxoplasma gondii* in domestic and wild animals from forest fragments of the municipality of Natal, northeastern Brazil. Rev Bras Parasitol Vet. 2014;23: 501–508. doi:10.1590/S1984-29612014092

186. Fredebaugh SL, Mateus-Pinilla NE, McAllister M, Warner RE, Weng H-Y. Prevalence of Antibody to <i>Toxoplasma gondii<i> in Terrestrial Wildlife in a Natural Area. Journal of Wildlife Diseases. 2011;47: 381–392. doi:10.7589/0090-3558-47.2.381

187. Frenkel JK, Hassanein KM, Hassanein RS, Brown E, Thulliez P, Quintero-Nunez R. Transmission of Toxoplasma gondii in Panama City, Panama: a five-year prospective cohort study of children, cats, rodents, birds, and soil. Am J Trop Med Hyg. 1995;53: 458–468. doi:10.4269/ajtmh.1995.53.458

188. Fu L-L, Yan C, Liu Z-Z, Kong D-L, Lv L, Shi N. [Isolation and identification of Toxoplasma gondii strains from cats in Xuzhou region]. Zhongguo Xue Xi Chong Bing Fang Zhi Za Zhi. 2014;26: 656–657, 668.

189. Funada M, Pena H, Soares R, Amaku M, Gennari S. Freqüência de parasitos gastrintestinais em cães e gatos atendidos em hospital-escola veterinário da cidade de São Paulo. Arquivo Brasileiro de Medicina Veterinária e Zootecnia. 2007;59: 1338–1340. doi:10.1590/S0102-09352007000500038

190. Furtado MM, Gennari SM, Ikuta CY, Jácomo AT de A, Morais ZM de, Pena HF de J, et al. Serosurvey of Smooth Brucella, Leptospira spp. and Toxoplasma gondii in Free-Ranging Jaguars (Panthera onca) and Domestic Animals from Brazil. PLOS ONE. 2015;10: e0143816. doi:10.1371/journal.pone.0143816

191. Garcia JL, Navarro IT, Ogawa L, Oliveira RC de. Seroprevalence of toxoplasma gondii in swine, bovine, ovine and equine, and their correlation with human, felines and canines, from farms in north region of Paraná state, Brazil. Cienc Rural. 1999;29: 91–97. doi:10.1590/S0103-84781999000100017

192. Garoussi MT, Mehrzad J, Baniassadi A, Khoshnegah J. Seroprevalence of brucellosis in different kinds of feline population in north-east of Iran. Comp Clin Pathol. 2018;27: 1155–1160. doi:10.1007/s00580-018-2714-5

193. Gates MC, Nolan TJ. Endoparasite prevalence and recurrence across different age groups of dogs and cats. Vet Parasitol. 2009;166: 153–158. doi:10.1016/j.vetpar.2009.07.041

194. Gauss CBL, Almería S, Ortuño A, Garcia F, Dubey JP. Seroprevalence of Toxoplasma gondii antibodies in domestic cats from Barcelona, Spain. J Parasitol. 2003;89: 1067–1068. doi:10.1645/GE-114

195. Genchi M, Vismarra A, Zanet S, Morelli S, Galuppi R, Cringoli G, et al. Prevalence and risk factors associated with cat parasites in Italy: a multicenter study. Parasites & Vectors. 2021;14: 475. doi:10.1186/s13071-021-04981-2

196. Gennari S, KASAI N, Pena H, Cortez A. Ocorrência de protozoários e helmintos em amostras de fezes de cães e gatos da cidade de São Paulo. Brazilian Journal of Veterinary Research and Animal Science. 1999;36. doi:10.1590/S1413-95961999000200006

197. Gennari SM, Ferreira JIG da S, Pena HF de J, Labruna MB, Azevedo S dos S. Frequency of gastrointestinal parasites in cats seen at the University of São Paulo Veterinary Hospital, Brazil. Rev Bras Parasitol Vet. 2016;25: 423–428. doi:10.1590/S1984-29612016082

198. Gennari SM, Pena HF de J, Blasques LS. Frequência de ocorrência de parasitos gastrintestinais em amostras de fezes de cães e gatos da cidade de São Paulo. Vet News. 2001;8: 10–12.

199. Giacomelli M, Follador N, Coppola LM, Martini M, Piccirillo A. Survey of Campylobacter spp. in owned and unowned dogs and cats in Northern Italy. The Veterinary Journal. 2015;204: 333–337. doi:10.1016/j.tvjl.2015.03.017

200. Giannelli A, Capelli G, Joachim A, Hinney B, Losson B, Kirkova Z, et al. Lungworms and gastrointestinal parasites of domestic cats: a European perspective. International Journal for Parasitology. 2017;47: 517–528. doi:10.1016/j.ijpara.2017.02.003

201. Gil H, Cano L, de Lucio A, Bailo B, de Mingo MH, Cardona GA, et al. Detection and molecular diversity of *Giardia duodenalis* and *Cryptosporidium* spp. in sheltered dogs and cats in Northern Spain. Infection, Genetics and Evolution. 2017;50: 62–69. doi:10.1016/j.meegid.2017.02.013

202. Glaus T, Hofmann-Lehmann R, Greene C, Glaus B, Wolfensberger C, Lutz H. Seroprevalence of Bartonella henselae infection and correlation with disease status in cats in Switzerland. J Clin Microbiol. 1997;35: 2883–2885.

203. Gomard Y, Lagadec E, Humeau L, Pinet P, Bureau S, Silva DD, et al. Feral cats do not play a major role in leptospirosis epidemiology on Reunion Island. Epidemiology & Infection. 2019;147: e97. doi:10.1017/S0950268819000190

204. Gonçalves-Oliveira J, Damasco PV, Assis MR da S, Freitas DE, Pessoa Junior AA, de Sousa LS, et al. Infectious endocarditis caused by Bartonella henselae associated with infected pets: two case reports. Journal of Medical Case Reports. 2023;17: 143. doi:10.1186/s13256-023-03839-8

205. Gonzales C, Vargas-Calla A, Gomez-Puerta LA, Robles K, Lopez-Urbina MT, Gonzalez AE. Seroprevalence of *Toxoplasma gondii* and associated risk factors in cats from Lima, Peru. Veterinary Parasitology: Regional Studies and Reports. 2022;31: 100733. doi:10.1016/j.vprsr.2022.100733

206. Gow A, Deborah J. Gow, Gow DJ, Edward J Hall, Hall EJ, D. A. Langton, et al. Prevalence of potentially pathogenic enteric organisms in clinically healthy kittens in the UK. Journal of Feline Medicine and Surgery. 2009;11: 655–662. doi:10.1016/j.jfms.2008.12.007

207. Gracia MJ, Marcén JM, Pinal R, Calvete C, Rodes D. Prevalence of Rickettsia and Bartonella species in Spanish cats and their fleas. Journal of Vector Ecology. 2015;40: 233–239. doi:10.1111/jvec.12159

208. Grandi G, Comin A, Ibrahim O, Schaper R, Forshell U, Lind EO. Prevalence of helminth and coccidian parasites in Swedish outdoor cats and the first report of Aelurostrongylus abstrusus in Sweden: a coprological investigation. Acta Veterinaria Scandinavica. 2017;59: 19. doi:10.1186/s13028-017-0287-y

209. Grillini M, Beraldo P, Frangipane di Regalbono A, Dotto G, Tessarin C, Franzo G, et al. Molecular survey of Cytauxzoon spp. and Hepatozoon spp. in felids using a novel real-time PCR approach. Frontiers in Veterinary Science. 2023;10. Available: https://www.frontiersin.org/articles/10.3389/fvets.2023.1113681

210. Gueldner EK, Gilli U, Strube C, Schnyder M. Seroprevalence, biogeographic distribution and risk factors for *Aelurostrongylus abstrusus* infections in Swiss cats. Veterinary Parasitology. 2019;266: 27–33. doi:10.1016/j.vetpar.2018.12.013

211. Gunn-Moore DA, McFarland SE, Brewer JI, Crawshaw TR, Clifton-Hadley RS, Kovalik M, et al. Mycobacterial disease in cats in Great Britain: I. Culture results, geographical distribution and clinical presentation of 339 cases. J Feline Med Surg. 2011;13: 934–944. doi:10.1016/j.jfms.2011.07.012

212. Gurfield AN, Boulouis HJ, Chomel BB, Heller R, Kasten RW, Yamamoto K, et al. Coinfection with Bartonella clarridgeiae and Bartonella henselae and with different Bartonella henselae strains in domestic cats. J Clin Microbiol. 1997;35: 2120–2123. doi:10.1128/jcm.35.8.2120-2123.1997

213. Gurfield AN, Boulouis HJ, Chomel BB, Kasten RW, Heller R, Bouillin C, et al. Epidemiology of Bartonella infection in domestic cats in France. Vet Microbiol. 2001;80: 185–198. doi:10.1016/s0378-1135(01)00304-2

214. Gürtler RE, Cecere MC, Lauricella MA, Cardinal MV, Kitron U, Cohen JE. Domestic dogs and cats as sources of Trypanosoma cruzi infection in rural northwestern Argentina. Parasitology. 2007;134: 69–82. doi:10.1017/S0031182006001259

215. Gutiérrez R, Morick D, Gross I, Winkler R, Abdeen Z, Harrus S. Bartonellae in domestic and stray cats from Israel: comparison of bacterial cultures and high-resolution melt real-time PCR as diagnostic methods. Vector Borne Zoonotic Dis. 2013;13: 857–864. doi:10.1089/vbz.2013.1308

216. Györke A, Opsteegh M, Mircean V, Iovu A, Cozma V. Toxoplasma gondii in Romanian household cats: Evaluation of serological tests, epidemiology and risk factors. Preventive Veterinary Medicine. 2011;102: 321–328. doi:10.1016/j.prevetmed.2011.07.015

217. Haddadzadeh HR, Khazraiinia P, Aslani M, Rezaeian M, Jamshidi S, Taheri M, et al. Seroprevalence of Toxoplasma gondii infection in stray and household cats in Tehran. Vet Parasitol. 2006;138: 211–216. doi:10.1016/j.vetpar.2006.02.010

218. Hajipour N, Imani Baran A, Yakhchali M, Banan Khojasteh SM, Sheikhzade Hesari F, Esmaeilnejad B, et al. A survey study on gastrointestinal parasites of stray cats in Azarshahr, (East Azerbaijan province, Iran). J Parasit Dis. 2016;40: 1255–1260. doi:10.1007/s12639-015-0663-3

219. Halánová M, Petrová L, Halán M, Trbolová A, Babinská I, Weissová T. Impact of way of life and environment on the prevalence of Chlamydia felis in cats as potentional sources of infection for humans. Ann Agric Environ Med. 2019;26: 222–226. doi:10.26444/aaem/100655

220. Hald B, Madsen M. Healthy puppies and kittens as carriers of Campylobacter spp., with special reference to Campylobacter upsaliensis. Journal of Clinical Microbiology. 1997;35: 3351–3352. doi:10.1128/jcm.35.12.3351-3352.1997

221. Haleche I, Guilane A, Boutellis A, Medrouh B, Saidi F, Kernif T, et al. Microscopic and molecular prevalence and associated risk factors with Toxocara and Blastocystis infection in dogs and cats in Mitidja, Algeria. Parasitol Res. 2024;123: 216. doi:10.1007/s00436-024-08240-y

222. Hamidinejat H, Mosalanejad B, Avizeh R, Jalali MHR. Neospora caninum and Toxoplasma gondii antibody prevalence in Ahvaz feral cats, Iran. Jundishapur Journal of Microbiology. 2011;4: 217–222.

223. Hammond-Aryee K, Esser M, van Helden L, van Helden P. A high seroprevalence of Toxoplasma gondii antibodies in a population of feral cats in the Western Cape province of South Africa. Southern African Journal of Infectious Diseases. 2015;30: 141–144. doi:10.1080/23120053.2015.1107295

224. Hartmann G, Roman IJ, Lorenzetti DM, Herbichi AP, Mazaro RD, dos Santos MY, et al. Anti-Leishmania spp. antibody detection in domestic cats from a visceral leishmaniasis transmission area. Parasitol Res. 2023;122: 2631–2639. doi:10.1007/s00436-023-07961-w

225. Hässle SN, Rodriguez-Campos S, Howard J, Speiser-Fontaine C, Schuller S. Serological detection of anti-leptospiral antibodies in outdoor cats in Switzerland. Veterinary and Animal Science. 2019;8: 100068. doi:10.1016/j.vas.2019.100068

226. Heikkilä HM, Bondarenko A, Mihalkov A, Pfister K, Spillmann T. Anaplasma phagocytophilum infection in a domestic cat in Finland: Case report. Acta Vet Scand. 2010;52: 62. doi:10.1186/1751-0147-52-62

227. Heller R, Artois M, Xemar V, De Briel D, Gehin H, Jaulhac B, et al. Prevalence of Bartonella henselae and Bartonella clarridgeiae in stray cats. J Clin Microbiol. 1997;35: 1327–1331. doi:10.1128/jcm.35.6.1327-1331.1997

228. Henry P, Huck-Gendre C, Franc M, Williams TL, Bouhsira E, Lienard E. Epidemiological Survey on Gastrointestinal and Pulmonary Parasites in Cats Around Toulouse (France). Helminthologia. 2022;59: 385–397. doi:10.2478/helm-2022-0036

229. Hill RE, Zimmerman JJ, Wills RW, Patton S, Clark WR. Seroprevalence of antibodies against Toxoplasma gondii in free-ranging mammals in Iowa. J Wildl Dis. 1998;34: 811–815. doi:10.7589/0090-3558-34.4.811

230. Hill SL, Cheney JM, Taton-Allen GF, Reif JS, Bruns C, Lappin MR. Prevalence of enteric zoonotic organisms in cats. Journal of the American Veterinary Medical Association. 2000;216: 687–692. doi:10.2460/javma.2000.216.687

231. Hoggard KR, Jarriel DM, Bevelock TJ, Verocai GG. Prevalence survey of gastrointestinal and respiratory parasites of shelter cats in northeastern Georgia, USA. Veterinary Parasitology: Regional Studies and Reports. 2019;16: 100270. doi:10.1016/j.vprsr.2019.100270

232. Holzapfel M, Taraveau F, Djelouadji Z. Serological and molecular detection of pathogenic *Leptospira* in domestic and stray cats on Reunion Island, French Indies. Epidemiol Infect. 2021;149: e229. doi:10.1017/S095026882100176X

233. Homayouni MM, Razavi SM, Shaddel M, Asadpour M. Prevalence and molecular characterization of Cryptosporidium spp. and Giardia intestinalisin household dogs and catsfrom Shiraz, Southwestern Iran. Veterinaria Italiana. 2020;55: 311–318. doi:10.12834/VetIt.1710.9049.3

234. Hong S, Choi JH, Oh S, Yi M, Kim SL, Kim M, et al. Gut microbiota differences induced by Toxoplasma gondii seropositivity in stray cats in South Korea. Parasitol Res. 2023;122: 2413–2421. doi:10.1007/s00436-023-07943-y

235. Hong S-H, Jeong Y-I, Kim J-Y, Cho S-H, Lee W-J, Lee S-E. Prevalence of Toxoplasma gondii Infection in Household Cats in Korea and Risk Factors. Korean J Parasitol. 2013;51: 357–361. doi:10.3347/kjp.2013.51.3.357

236. Hou Z, Su S, Liu D, Wang L, Jia C, Zhao Z, et al. Prevalence, risk factors and genetic characterization of *Toxoplasma gondii* in sick pigs and stray cats in Jiangsu Province, eastern China. Infection, Genetics and Evolution. 2018;60: 17–25. doi:10.1016/j.meegid.2018.02.007

237. Hsu V, Grant DC, Zajac AM, Witonsky SG, Lindsay DS. Prevalence of IgG antibodies to *Encephalitozoon cuniculi* and *Toxoplasma gondii* in cats with and without chronic kidney disease from Virginia. Veterinary Parasitology. 2011;176: 23–26. doi:10.1016/j.vetpar.2010.10.022

238. Huertas-López A, Sukhumavasi W, Álvarez-García G, Martínez-Subiela S, Cano-Terriza D, Almería S, et al. Seroprevalence of Toxoplasma gondii in outdoor dogs and cats in Bangkok, Thailand. Parasitology. 2021;148: 843–849. doi:10.1017/S0031182021000421

239. Hwang J, Gottdenker N, Min M-S, Lee H, Chun M-S. Evaluation of biochemical and haematological parameters and prevalence of selected pathogens in feral cats from urban and rural habitats in South Korea. Journal of Feline Medicine and Surgery. 2016;18: 443–451. doi:10.1177/1098612X15587572

240. Idan S, Al-Hasnawy M. Microscopic and molecular diagnoses of Giardia duodenalis in pet animals in Babylon Province, Iraq. Veterinary World. 1013;16: 2263–2270. doi:10.14202/vetworld.2023.2263-2270

241. Ilić T, Kulišić Z, Antić N, Radisavljević K, Dimitrijević S. Prevalence of zoonotic intestinal helminths in pet dogs and cats in the Belgrade area. Journal of Applied Animal Research. 2017;45: 204–208. doi:10.1080/09712119.2016.1141779

242. Inoue K, Maruyama S, Kabeya H, Kawanami K, Yanai K, Jitchum S, et al. Prevalence of Bartonella infection in cats and dogs in a metropolitan area, Thailand. Epidemiology & Infection. 2009;137: 1568–1573. doi:10.1017/S095026880900257X

243. Inpankaew T, Sattasathuchana P, Kengradomkij C, Thengchaisri N. Prevalence of toxoplasmosis in semi-domesticated and pet cats within and around Bangkok, Thailand. BMC Vet Res. 2021;17: 252. doi:10.1186/s12917-021-02965-z

244. Ito Y, Itoh N, Kimura Y, Kanai K. Molecular detection and characterization of Cryptosporidium spp. among breeding cattery cats in Japan. Parasitol Res. 2016;115: 2121–2123. doi:10.1007/s00436-016-4984-7

245. Itoh N, Ikegami H, Takagi M, Ito Y, Kanai K, Chikazawa S, et al. Prevalence of intestinal parasites in private-household cats in Japan. J Feline Med Surg. 2012;14: 436–439. doi:10.1177/1098612X12443633

246. Itoh N, Muraoka N, Kawamata J, Aoki M, Itagaki T. Prevalence of Giardia intestinalis infection in household cats of Tohoku district in Japan. J Vet Med Sci. 2006;68: 161–163. doi:10.1292/jvms.68.161

247. Iturbe Cossío TL, Montes Luna AD, Ruiz Mejia M, Flores Ortega A, Heredia Cárdenas R, Romero Núñez C. Risk factors associated with cat parasites in a feline medical center. JFMS Open Rep. 2021;7: 20551169211033183. doi:10.1177/20551169211033183

248. Jameson P, Greene C, Regnery R, Dryden M, Marks A, Brown J, et al. Prevalence of Bartonella henselae antibodies in pet cats throughout regions of North America. J Infect Dis. 1995;172: 1145–1149. doi:10.1093/infdis/172.4.1145

249. Jamshidi S, Akhavizadegan M, Bokaie S, Maazi N, Ghorban A. Serologic study of feline leptospirosis in Tehran, Iran. Iranian Journal of Microbiology. 2009;1: 32–36.

250. Jamshidi Sh, Tabrizi AS, Bahrami M, Momtaz H. Microsporidia in household dogs and cats in Iran; a zoonotic concern. Veterinary Parasitology. 2012;185: 121–123. doi:10.1016/j.vetpar.2011.10.002

251. Javadi S, Asri Rezaei S, Tajik H, Hadian M, Shokouhi F. Haematological changes of cats with Toxoplasma gondii-specific antibodies. Comp Clin Pathol. 2010;19: 307–310. doi:10.1007/s00580-009-0869-9

252. Jenkins KS, Dittmer KE, Marshall JC, Tasker S. Prevalence and risk factor analysis of feline haemoplasma infection in New Zealand domestic cats using a real-time PCR assay. Journal of Feline Medicine and Surgery. 2013;15: 1063–1069. doi:10.1177/1098612X13488384

253. Jiménez-Coello M, Acosta-Viana KY, Guzman-Marin E, Gomez-Rios A, Ortega-Pacheco A. Epidemiological survey of Trypanosoma cruzi infection in domestic owned cats from the tropical southeast of Mexico. Zoonoses Public Health. 2012;59 Suppl 2: 102–109. doi:10.1111/j.1863-2378.2012.01463.x

254. Jimenez-Coello M, Acosta-Viana KY, Guzman-MarÃ­n E, Gutierrez-ruiz EJ, Rodriguez-Vivas RI, Bolio-Gonzalez ME, et al. The Occurrence Of Toxoplasma Gondii Antibodies In Backyard Pigs And Cats From An Endemic Tropical Area Of Mexico. Tropical and Subtropical Agroecosystems. 2013;16. Available: https://www.revista.ccba.uady.mx/ojs/index.php/TSA/article/view/1294

255. Jitsamai W, Khrutkham N, Hunprasit V, Chandrashekar R, Bowman D, Sukhumavasi W. Prevalence of endoparasitic and viral infections in client-owned cats in metropolitan Bangkok, Thailand, and the risk factors associated with feline hookworm infections. Veterinary Parasitology: Regional Studies and Reports. 2021;25: 100584. doi:10.1016/j.vprsr.2021.100584

256. Jittapalapong S, Nimsupan B, Pinyopanuwat N, Chimnoi W, Kabeya H, Maruyama S. Seroprevalence of *Toxoplasma gondii* antibodies in stray cats and dogs in the Bangkok metropolitan area, Thailand. Veterinary Parasitology. 2007;145: 138–141. doi:10.1016/j.vetpar.2006.10.021

257. Joffe D, Van Niekerk D, Gagné F, Gilleard J, Kutz S, Lobingier R. The prevalence of intestinal parasites in dogs and cats in Calgary, Alberta. Can Vet J. 2011;52: 1323–1328.

258. Johnson EM, Nagamori Y, Duncan-Decocq RA, Whitley PN, Ramachandran A, Reichard MV. Prevalence of Alaria infection in companion animals in north central Oklahoma from 2006 through 2015 and detection in wildlife. 2017 [cited 29 Dec 2024]. doi:10.2460/javma.250.8.881

259. Jokelainen P, Simola O, Rantanen E, Näreaho A, Lohi H, Sukura A. Feline toxoplasmosis in Finland: cross-sectional epidemiological study and case series study. J VET Diagn Invest. 2012;24: 1115–1124. doi:10.1177/1040638712461787

260. Jung B-K, Lee S-E, Lim H, Cho J, Kim D-G, Song H, et al. Toxoplasma gondii B1 Gene Detection in Feces of Stray Cats around Seoul, Korea and Genotype Analysis of Two Laboratory-Passaged Isolates. Korean J Parasitol. 2015;53: 259–263. doi:10.3347/kjp.2015.53.3.259

261. Jungwirth N, Puff C, Köster K, Mischke R, Meyer H, Stark A, et al. Atypical Cowpox Virus Infection in a Series of Cats. Journal of Comparative Pathology. 2018;158: 71–76. doi:10.1016/j.jcpa.2017.12.003

262. Juvet F, Lappin MR, Brennan S, Mooney CT. Prevalence of selected infectious agents in cats in Ireland. Journal of Feline Medicine and Surgery. 2010;12: 476–482. doi:10.1016/j.jfms.2010.02.003

263. Kajero OT, Janoušková E, Bakare EA, Belizario V, Divina B, Alonte AJ, et al. Co-infection of intestinal helminths in humans and animals in the Philippines. Trans R Soc Trop Med Hyg. 2022;116: 727–735. doi:10.1093/trstmh/trac002

264. Kakita T, Kuba Y, Kyan H, Okano S, Morita M, Koizumi N. Molecular and serological epidemiology of Leptospira infection in cats in Okinawa Island, Japan. Sci Rep. 2021;11: 10365. doi:10.1038/s41598-021-89872-3

265. Kalef DA, Al-Khayat FAA-M. A Comparative Study of Some Intestinal Parasites in Fecal Samples of Domestic and Stray Cats in Baghdad, Iraq. copa. 2022;89: 30–34. doi:10.1654/21-00015

266. Kamani J, Mani A, Kumshe H, Yidawi J, Egwu G. Prevalence of Toxoplasma gondii antibodies in cats in maiduguri, northeastern nigeria. Acta Parasitologica. 2010;55: 94–95. doi:10.2478/s11686-010-0015-5

267. Kamrani A, Parreira VR, Greenwood J, Prescott JF. The prevalence of Bartonella, hemoplasma, and Rickettsia felis infections in domestic cats and in cat fleas in Ontario. Can J Vet Res. 2008;72: 411–419.

268. Kang Y-H, Cong W, Qin S-Y, Shan X-F, Gao Y-H, Wang C-F, et al. First Report of Toxoplasma gondii, Dirofilaria immitis, and Chlamydia felis Infection in Stray and Companion Cats in Northeastern and Eastern China. Vector Borne Zoonotic Dis. 2016;16: 654–658. doi:10.1089/vbz.2016.1993

269. Karimi P, Shafaghi-Sisi S, Meamar AR, Nasiri G, Razmjou E. Prevalence and Molecular Characterization of Toxoplasma gondii and Toxocara cati Among Stray and Household Cats and Cat Owners in Tehran, Iran. Front Vet Sci. 2022;9: 927185. doi:10.3389/fvets.2022.927185

270. Karimi P, Shafaghi-Sisi S, Meamar AR, Razmjou E. Molecular identification of Cryptosporidium, Giardia, and Blastocystis from stray and household cats and cat owners in Tehran, Iran. Sci Rep. 2023;13: 1554. doi:10.1038/s41598-023-28768-w

271. Kausar K, Muhammad I. Assessment of Toxoplasma gondii Contamination in Cat Feces. IJLAI Transactions on Science and Engineering. 2024;2: 1–10.

272. Kelly PJ, Matthewman LA, Hayter D, Downey S, Wray K, Bryson NR, et al. Bartonella (Rochalimaea) henselae in southern Africa--evidence for infections in domestic cats and implications for veterinarians. J S Afr Vet Assoc. 1996;67: 182–187.

273. Ketzis JK, Shell L, Chinault S, Pemberton C, Pereira MM. The prevalence of Trichuris spp. infection in indoor and outdoor cats on St. Kitts. J Infect Dev Ctries. 2015;9: 111–113. doi:10.3855/jidc.5778

274. Khademvatan S, Abdizadeh R, Rahim F, Hashemitabar M, Ghasemi M, Tavalla M. Stray Cats Gastrointestinal Parasites and its Association With Public Health in Ahvaz City, South Western of Iran. Jundishapur J Microbiol. 2014;7: e11079. doi:10.5812/jjm.11079

275. Khalafalla RE. A Survey Study on Gastrointestinal Parasites of Stray Cats in Northern Region of Nile Delta, Egypt. PLOS ONE. 2011;6: e20283. doi:10.1371/journal.pone.0020283

276. Khodaverdi M, Razmi G. A serological and parasitological study of Toxoplasma gondii infection in stray cats of Mashhad, Khorasan Razavi province, Iran. Vet Res Forum. 2019;10: 119–123. doi:10.30466/vrf.2019.71293.1975

277. Khomayezi RKAS, Abdollahpoor G. Sero-prevalence of leptospira spp. in household and stray cats by microscopic agglutination test. INTERNATIONAL JOURNAL OF CURRENT RESEARCH. 2015;7: 11534–11537.

278. Kim H-Y, Kim Y-A, Kang S, Lee HS, Rhie HG, Ahn H-J, et al. Prevalence of Toxoplasma gondii in Stray Cats of Gyeonggi-do, Korea. Korean J Parasitol. 2008;46: 199–201. doi:10.3347/kjp.2008.46.3.199

279. Kim S-E, Choi R, Kang S-W, Hyun C. Prevalence of Toxoplasma gondii infection in household and feral cats in Korea. J Parasit Dis. 2017;41: 823–825. doi:10.1007/s12639-017-0896-4

280. Kiszely S, Gyurkovszky M, Solymosi N, Farkas R. Survey of lungworm infection of domestic cats in Hungary. Acta Veterinaria Hungarica. 2019;67: 407–417. doi:10.1556/004.2019.041

281. Kittl S, Francey T, Brodard I, Origgi FC, Borel S, Ryser-Degiorgis M-P, et al. First European report of Francisella tularensis subsp. holarctica isolation from a domestic cat. Vet Res. 2020;51: 109. doi:10.1186/s13567-020-00834-5

282. Knapp J, Combes B, Umhang G, Aknouche S, Millon L. Could the domestic cat play a significant role in the transmission of Echinococcus multilocularis? A study based on qPCR analysis of cat feces in a rural area in France. Parasite. 2016;23: 42. doi:10.1051/parasite/2016052

283. Knaus M, Rapti D, Shukullari E, Kusi I, Postoli R, Xhaxhiu D, et al. Characterisation of ecto- and endoparasites in domestic cats from Tirana, Albania. Parasitol Res. 2014;113: 3361–3371. doi:10.1007/s00436-014-3999-1

284. Koehler JE, Glaser CA, Tappero JW. Rochalimaea henselae infection. A new zoonosis with the domestic cat as reservoir. JAMA. 1994;271: 531–535. doi:10.1001/jama.271.7.531

285. Kokkinaki KCG, Saridomichelakis MN, Mylonakis ME, Leontides L, Xenoulis PG. Seroprevalence of and Risk Factors for Toxoplasma gondii Infection in Cats from Greece. Animals (Basel). 2023;13: 1173. doi:10.3390/ani13071173

286. Koompapong K, Mori H, Thammasonthijarern N, Prasertbun R, Pintong A, Popruk S, et al. Molecular identification of Cryptosporidium spp. in seagulls, pigeons, dogs, and cats in Thailand. Parasite. 2014;21: 52. doi:10.1051/parasite/2014053

287. Köseoğlu AE, Can H, Karakavuk M, Güvendi M, Değirmenci Döşkaya A, Manyatsi PB, et al. Molecular prevalence and subtyping of Cryptosporidium spp. in fecal samples collected from stray cats in İzmir, Turkey. BMC Veterinary Research. 2022;18: 89. doi:10.1186/s12917-022-03190-y

288. Kostopoulou D, Claerebout E, Arvanitis D, Ligda P, Voutzourakis N, Casaert S, et al. Abundance, zoonotic potential and risk factors of intestinal parasitism amongst dog and cat populations: The scenario of Crete, Greece. Parasit Vectors. 2017;10: 43. doi:10.1186/s13071-017-1989-8

289. Kulasena VA, Rajapakse RPVJ, Dubey JP, Dayawansa PN, Premawansa S. Seroprevalence of Toxoplasma gondii in Cats from Colombo, Sri Lanka. para. 2011;97: 152–152. doi:10.1645/GE-2640.1

290. Kurnosova OP, Panova OA, Arisov MV. The prevalence of potentially zoonotic intestinal parasites in dogs and cats in Moscow, Russia. Helminthologia. 2023;60: 44–51. doi:10.2478/helm-2023-0009

291. Kyan H, Takara T, Taira K, Obi T. Toxoplasma gondii antibody prevalence and isolation in free-ranging cats in Okinawa, Japan. J Vet Med Sci. 2021;83: 1303–1305. doi:10.1292/jvms.21-0038

292. Labarthe N, Serrão ML, Ferreira AMR, Almeida NKO, Guerrero J. A survey of gastrointestinal helminths in cats of the metropolitan region of Rio de Janeiro, Brazil. Vet Parasitol. 2004;123: 133–139. doi:10.1016/j.vetpar.2004.06.002

293. Laberke S, Just F, Pfister K, Hartmann K. Prevalence of feline haemoplasma infection in cats in Southern Bavaria, Germany, and infection risk factor analysis. Berl Munch Tierarztl Wochenschr. 2010;123: 42–48.

294. Labruna MB, Ogrzewalska M, Moraes-Filho J, Lepe P, Gallegos JL, López J. Rickettsia felis in Chile. Emerg Infect Dis. 2007;13: 1794–1795. doi:10.3201/eid1311.070782

295. Lamas C, Favacho A, Ramos RG, Santos MS, Ferravoli GI, Weksler C, et al. Bartonella native valve endocarditis: the first brazilian case alive and well. Braz J Infect Dis. 2007;11: 591–594. doi:10.1590/S1413-86702007000600012

296. Lapointe C, Plamondon I, Dunn M. Feline leptospirosis serosurvey from a Quebec referral hospital. The Canadian Veterinary Journal. 2013;54: 497.

297. Lappin MR, Breitschwerdt EB, Jensen WA, Dunnigan B, Rha J-Y, Williams CR, et al. Molecular and serologic evidence of Anaplasma phagocytophilum infection in cats in North America. Journal of the American Veterinary Medical Association. 2004;225: 893–896. doi:10.2460/javma.2004.225.893

298. Lappin MR, Griffin B, Brunt J, Riley A, Burney D, Hawley J, et al. Prevalence of Bartonella species, haemoplasma species, Ehrlichia species, Anaplasma phagocytophilum, and Neorickettsia risticii DNA in the blood of cats and their fleas in the United States. J Feline Med Surg. 2006;8: 85–90. doi:10.1016/j.jfms.2005.08.003

299. Lappin MR, Marks A, Greene CE, Collins JK, Carman J, Reif JS, et al. Serologic prevalence of selected infectious diseases in cats with uveitis. Journal of the American Veterinary Medical Association. 1992;201: 1005–1009. doi:10.2460/javma.1992.201.07.1005

300. Lecca LO, Paiva MT, de Oliveira CSF, Morais MHF, de Azevedo MI, Bastos C de VE, et al. Associated factors and spatial patterns of the epidemic sporotrichosis in a high density human populated area: A cross-sectional study from 2016 to 2018. Prev Vet Med. 2020;176: 104939. doi:10.1016/j.prevetmed.2020.104939

301. Lecová L, Hammerbauerová I, Tůmová P, Nohýnková E. Companion animals as a potential source of *Giardia intestinalis* infection in humans in the Czech Republic – A pilot study. Veterinary Parasitology: Regional Studies and Reports. 2020;21: 100431. doi:10.1016/j.vprsr.2020.100431

302. Lee S-E, Kim J-Y, Kim Y-A, Cho S-H, Ahn H-J, Woo H-M, et al. Prevalence of Toxoplasma gondii Infection in Stray and Household Cats in Regions of Seoul, Korea. Korean J Parasitol. 2010;48: 267–270. doi:10.3347/kjp.2010.48.3.267

303. Lee S-E, Kim N-H, Chae H-S, Cho S-H, Nam H-W, Lee W-J, et al. Prevalence of Toxoplasma gondii Infection in Feral Cats in Seoul, Korea. Journal of Parasitology. 2011;97: 153–155. doi:10.1645/GE-2455.1

304. Lee S-H, Ock Y, Choi D, Kwak D. Gastrointestinal Parasite Infection in Cats in Daegu, Republic of Korea, and Efficacy of Treatment Using Topical Emodepside/Praziquantel Formulation. Korean J Parasito. 2019;57: 243–248. doi:10.3347/kjp.2019.57.3.243

305. Lefkaditis MA, Pastiu AI, Rodi-Buriel A, Sossidou AV, Panorias AH, Eleftheriadis TG, et al. Helminth burden in stray cats from Thessaloniki, Greece. Helminthologia. 2014;51: 73–76. doi:10.2478/s11687-014-0211-1

306. Lehtla A, Must K, Lassen B, Orro T, Jokelainen P, Viltrop A. Leptospira spp. in Cats in Estonia: Seroprevalence and Risk Factors for Seropositivity. Vector-Borne and Zoonotic Diseases. 2020;20: 524–528. doi:10.1089/vbz.2019.2555

307. Leiby PD, Kritsky DC. Echinococcus multilocularis: A Possible Domestic Life Cycle in Central North America and Its Public Health Implications. The Journal of Parasitology. 1972;58: 1213–1215. doi:10.2307/3278173

308. Li J, Dan X, Zhu K, Li N, Guo Y, Zheng Z, et al. Genetic characterization of Cryptosporidium spp. and Giardia duodenalis in dogs and cats in Guangdong, China. Parasites Vectors. 2019;12: 571. doi:10.1186/s13071-019-3822-z

309. Li L, Sui Y, Li X, Song P, Chen G, Liu H, et al. Molecular characterization of *Cryptosporidium* spp. and *Giardia duodenalis* in pet cats in Henan Province, central China. Acta Tropica. 2024;254: 107188. doi:10.1016/j.actatropica.2024.107188

310. Li W-C, Qin J, Wang K, Gu Y-F. Genotypes of Enterocytozoon bieneusi in Dogs and Cats in Eastern China. Iran J Parasitol. 2018;13: 457–465.

311. Li W, Liu X, Gu Y, Liu J, Luo J. Prevalence of Cryptosporidium, Giardia, Blastocystis, and trichomonads in domestic cats in East China. Journal of Veterinary Medical Science. 2019;81: 890–896. doi:10.1292/jvms.19-0111

312. Li X-T, Wang L, Ding Y, Sun W-W. Toxoplasma gondii infection in pet cats and their owners in northeastern China:an important public health concern. BMC Vet Res. 2022;18: 9. doi:10.1186/s12917-021-03110-6

313. Li Y-N, Nie X, Peng Q-Y, Mu X-Q, Zhang M, Tian M-Y, et al. Seroprevalence and genotype of Toxoplasma gondii in pigs, dogs and cats from Guizhou province, Southwest China. Parasites Vectors. 2015;8: 214. doi:10.1186/s13071-015-0809-2

314. Lima MLF, Soares PT, Ramos CAN, Araújo FR, Ramos RAN, Souza IIF, et al. Molecular detection of Anaplasma platys in a naturally-infected cat in Brazil. Braz J Microbiol. 2010;41: 381–385. doi:10.1590/S1517-838220100002000019

315. Lima VFS, Ramos RAN, Lepold R, Borges JCG, Ferreira CD, Rinaldi L, et al. Gastrointestinal parasites in feral cats and rodents from the Fernando de Noronha Archipelago, Brazil. Rev Bras Parasitol Vet. 2017;26: 521–524. doi:https://doi.org/10.1590/S1984-29612017066

316. Lin D-S, Lai S-S, Bowman DD, Jacobson RH, Barr MC, Giovengo SL. Feline immunodeficiency virus, feline leukaemia virus, *Toxoplasma gondii*, and intestinal parasitic infections in Taiwanese cats. British Veterinary Journal. 1990;146: 468–475. doi:10.1016/0007-1935(90)90037-4

317. Little S, Adolph C, Downie K, Snider T, Reichard M. High Prevalence of Covert Infection With Gastrointestinal Helminths in Cats. J Am Anim Hosp Assoc. 2015;51: 359–364. doi:10.5326/JAAHA-MS-6221

318. Liu Q-X, Wang S, Wang L-Q, Xing J, Gao W-J, Liu G-F, et al. Seroprevalence of *Toxoplasma gondii* infection in dogs and cats in Zhenjiang City, Eastern China. Asian Pacific Journal of Tropical Biomedicine. 2014;4: 725–728. doi:10.12980/APJTB.4.2014APJTD-2014-0109

319. Liyanage KLDTD, Amery-Gale J, Uboldi AD, Adriaanse K, Firestone SM, Tonkin CJ, et al. Seroprevalence and risk factors for *Toxoplasma gondii* exposure in Australian feral and stray cats using an in-house modified agglutination test. Veterinary Parasitology. 2024;332: 110306. doi:10.1016/j.vetpar.2024.110306

320. Lobetti R, Lappin MR. Prevalence of Toxoplasma gondii, Bartonella species and haemoplasma infection in cats in South Africa. Journal of Feline Medicine and Surgery. 2012;14: 857–862. doi:10.1177/1098612X12452495

321. Lopes AP, Cardoso L, Rodrigues M. Serological survey of Toxoplasma gondii infection in domestic cats from northeastern Portugal. Vet Parasitol. 2008;155: 184–189. doi:10.1016/j.vetpar.2008.05.007

322. Lopes AP, Oliveira AC, Granada S, Rodrigues FT, Papadopoulos E, Schallig H, et al. Antibodies to Toxoplasma gondii and Leishmania spp. in domestic cats from Luanda, Angola. Vet Parasitol. 2017;239: 15–18. doi:10.1016/j.vetpar.2017.04.009

323. López C, Daprato B, Zampolini, Cardillo N, Sommerfelt I. Risk factors and prevalence of IgG antibodies to Toxoplasma gondii in domestic cats. La Matanza, Buenos Aires, Argentina. Rev Ibero-Latinoam Parasitol. 2011;70: 29–34.

324. López J, Abarca V K, Paredes M P, Inzunza T E. Intestinal parasites in dogs and cats with gastrointestinal symptoms in Santiago, Chile. Revista médica de Chile. 2006;134: 193–200. doi:10.4067/S0034-98872006000200009

325. Lucas SR, Hagiwara MK, Loureiro V d, Ikesaki JY, Birgel EH. Toxoplasma gondii infection in Brazilian domestic outpatient cats. Rev Inst Med Trop Sao Paulo. 1999;41: 221–224. doi:10.1590/S0036-46651999000400003

326. Luria BJ, Levy JK, Lappin MR, Breitschwerdt EB, Legendre AM, Hernandez JA, et al. Prevalence of infectious diseases in feral cats in Northern Florida. J Feline Med Surg. 2004;6: 287–296. doi:10.1016/j.jfms.2003.11.005

327. Ma GC, Norris JM, Mathews KO, Chandra S, Šlapeta J, Bosward KL, et al. New insights on the epidemiology of *Coxiella burnetii* in pet dogs and cats from New South Wales, Australia. Acta Tropica. 2020;205: 105416. doi:10.1016/j.actatropica.2020.105416

328. Magalhães FJR, Ribeiro-Andrade M, Souza FM, Lima Filho CDF, Biondo AW, Vidotto O, et al. Seroprevalence and spatial distribution of Toxoplasma gondii infection in cats, dogs, pigs and equines of the Fernando de Noronha Island, Brazil. Parasitol Int. 2017;66: 43–46. doi:10.1016/j.parint.2016.11.014

329. Mai LTP, Dung LP, Than PD, Dinh TV, Quyet NT, Hai H, et al. Leptospira infection among human-close-contact animals in different geographical areas in Vietnam. Sci Prog. 2021;104: 368504211031747. doi:10.1177/00368504211031747

330. Maia C, Catarino AL, Almeida B, Ramos C, Campino L, Cardoso L. Emergence of Thelazia callipaeda Infection in Dogs and Cats from East-Central Portugal. Transboundary and Emerging Diseases. 2016;63: 416–421. doi:10.1111/tbed.12284

331. Maia C, Gomes J, Cristóvão J, Nunes M, Martins A, Rebêlo E, et al. Feline Leishmania infection in a canine leishmaniasis endemic region, Portugal. Veterinary Parasitology. 2010;174: 336–340. doi:10.1016/j.vetpar.2010.08.030

332. Maia C, Ramos C, Coimbra M, Bastos F, Martins A, Pinto P, et al. Bacterial and protozoal agents of feline vector-borne diseases in domestic and stray cats from southern Portugal. Parasit Vectors. 2014;7: 115. doi:10.1186/1756-3305-7-115

333. Maia C, Ramos C, Coimbra M, Cardoso L, Campino L. Prevalence of Dirofilaria immitis antigen and antibodies to Leishmania infantum in cats from southern Portugal. Parasitol Int. 2015;64: 154–156. doi:10.1016/j.parint.2014.11.006

334. Majid A, Ahmad N, Haleem S, Akbar N ul, Zareen S, Taib M, et al. Detection of toxoplasmosis in pets and stray cats through molecular and serological techniques in Khyber Pakhtunkhwa, Pakistan. BMC Veterinary Research. 2021;17: 357. doi:10.1186/s12917-021-03064-9

335. Malloy WF, Embil JA. Prevalence of Toxocara spp. and other parasites in dogs and cats in Halifax, Nova Scotia. Can J Comp Med. 1978;42: 29–31.

336. Mancianti F, Nardoni S, Ariti G, Parlanti D, Giuliani G, Papini RA. Cross-sectional survey of Toxoplasma gondii infection in colony cats from urban Florence (Italy). Journal of Feline Medicine and Surgery. 2010;12: 351–354. doi:10.1016/j.jfms.2009.09.001

337. Mancianti F, Nardoni S, Mugnaini L, Zambernardi L, Guerrini A, Gazzola V, et al. A retrospective molecular study of select intestinal protozoa in healthy pet cats from Italy. Journal of Feline Medicine and Surgery. 2015;17: 163–167. doi:10.1177/1098612X14533549

338. Marbella D, Santana-Hernández KM, Rodríguez-Ponce E. Small islands as potential model ecosystems for parasitology: climatic influence on parasites of feral cats. Journal of Helminthology. 2022;96: e51. doi:10.1017/S0022149X22000451

339. Marder G, Ulon SN, Bottinelli OR, Fleitas ZM, Lotero DA, Ruiz R, et al. Infestación parasitaria en suelos y materia fecal de perros y gatos de la ciudad de Corrientes. Revista Veterinaria. 2004;15: 70–72. doi:10.30972/vet.1521999

340. Markovich J e., Ross L, McCobb E. The Prevalence of Leptospiral Antibodies in Free Roaming Cats in Worcester County, Massachusetts. Journal of Veterinary Internal Medicine. 2012;26: 688–689. doi:10.1111/j.1939-1676.2012.00900.x

341. Marston EL, Finkel B, Regnery RL, Winoto IL, Graham RR, Wignal S, et al. Prevalence of Bartonella henselae and Bartonella clarridgeiae in an Urban Indonesian Cat Population. Clin Diagn Lab Immunol. 1999;6: 41–44.

342. Martínez-Barbabosa I, Vázquez Tsuji O, Cabello RR, Cárdenas EMG, Chasin OA. The prevalence of Toxocara cati in domestic cats in Mexico City. Vet Parasitol. 2003;114: 43–49. doi:10.1016/s0304-4017(03)00038-4

343. Maruyama S, Kabeya H, Nakao R, Tanaka S, Sakai T, Xuan X, et al. Seroprevalence of Bartonella henselae, Toxoplasma gondii, FIV and FeLV infections in domestic cats in Japan. Microbiol Immunol. 2003;47: 147–153. doi:10.1111/j.1348-0421.2003.tb02798.x

344. Masucci M, Donato G, Persichetti MF, Priolo V, Castelli G, Bruno F, et al. Hemogram Findings in Cats from an Area Endemic for Leishmania infantum and Feline Immunodeficiency Virus Infections. Vet Sci. 2022;9: 508. doi:10.3390/vetsci9090508

345. Mateo M, Montoya A, Bailo B, Köster PC, Dashti A, Hernández-Castro C, et al. Prevalence and public health relevance of enteric parasites in domestic dogs and cats in the region of Madrid (Spain) with an emphasis on Giardia duodenalis and Cryptosporidium sp. Veterinary Medicine and Science. 2023;9: 2542–2558. doi:10.1002/vms3.1270

346. Matsuu A, Yokota S-I, Ito K, Masatani T. Seroprevalence of Toxoplasma gondii in free-ranging and feral cats on Amami Oshima Island, Japan. J Vet Med Sci. 2017;79: 1853–1856. doi:10.1292/jvms.17-0359

347. Matthewman L, Kelly P, Hayter D, Downie S, Wray K, Bryson N, et al. Domestic Cats as Indicators of the Presence of Spotted Fever and Typhus Group Rickettsiae. European Journal of Epidemiology. 1997;13: 109–111.

348. Mazzotta E, De Zan G, Cocchi M, Boniotti MB, Bertasio C, Furlanello T, et al. Feline Susceptibility to Leptospirosis and Presence of Immunosuppressive Co-Morbidities: First European Report of L. interrogans Serogroup Australis Sequence Type 24 in a Cat and Survey of Leptospira Exposure in Outdoor Cats. Trop Med Infect Dis. 2023;8: 54. doi:10.3390/tropicalmed8010054

349. Mccown ME, Grzeszak B. Zoonotic and infectious disease surveillance in Central America: Honduran feral cats positive for toxoplasma, trypanosoma, leishmania, rickettsia, and Lyme disease. J Spec Oper Med. 2010;10: 41–43. doi:10.55460/13sq-ok4v

350. McGlade TR, Robertson ID, Elliot AD, Read C, Thompson RCA. Gastrointestinal parasites of domestic cats in Perth, Western Australia. Veterinary Parasitology. 2003;117: 251–262. doi:10.1016/j.vetpar.2003.08.010

351. McReynolds CA, Lappin MR, Ungar B, McReynolds LM, Bruns C, Spilker MM, et al. Regional seroprevalence of *Cryptosporidium parvum*-specific IgG of cats in the United States. Veterinary Parasitology. 1999;80: 187–195. doi:10.1016/S0304-4017(98)00219-2

352. Meireles LR, Galisteo AJ, Pompeu E, Andrade HF. Toxoplasma gondii spreading in an urban area evaluated by seroprevalence in free-living cats and dogs. Trop Med Int Health. 2004;9: 876–881. doi:10.1111/j.1365-3156.2004.01280.x

353. Melo RPB, Almeida JC, Lima DCV, Pedrosa CM, Magalhães FJR, Alcântara AM, et al. Atypical Toxoplasma gondii genotype in feral cats from the Fernando de Noronha Island, northeastern Brazil. Vet Parasitol. 2016;224: 92–95. doi:10.1016/j.vetpar.2016.05.023

354. Meloni BP, Thompson RCA, Hopkins RM, Reynoldson JA, Gracey M. The prevalence of Giardia and other intestinal parasites in children, dogs and cats from Aboriginal communities in the Kimberley. Medical Journal of Australia. 1993;158: 157–159. doi:10.5694/j.1326-5377.1993.tb121692.x

355. Miceli NG, Gavioli FA, Gonçalves LR, André MR, Sousa VRF, Sousa KCM de, et al. Molecular detection of feline arthropod-borne pathogens in cats in Cuiabá, state of Mato Grosso, central-western region of Brazil. Rev Bras Parasitol Vet. 2013;22: 385–390. doi:10.1590/S1984-29612013000300011

356. Michaelian T, Harriott L, Gentle M, Proboste T, Ho IK, Cobbold R. Prevalence of pathogens important to human and companion animal health in an urban unowned cat population. wilr. 2024;51. doi:10.1071/WR22112

357. Michalski MM, Platt-Samoraj A, Mikulska-Skupien E. Toxoplasma gondii antibodies in domestic cats in Olsztyn urban area, Poland. Wiadomości Parazytologiczne. 2010;56. Available: http://agro.icm.edu.pl/agro/element/bwmeta1.element.agro-article-e67e3dc9-7e2b-4d94-95df-3d57bf122cd9

358. Michelitsch A, Hoffmann D, Wernike K, Beer M. Occurrence of Antibodies against SARS-CoV-2 in the Domestic Cat Population of Germany. Vaccines. 2020;8: 772. doi:10.3390/vaccines8040772

359. Millán J, Cabezón O, Pabón M, Dubey JP, Almería S. Seroprevalence of *Toxoplasma gondii* and *Neospora caninum* in feral cats (*Felis silvestris catus*) in Majorca, Balearic Islands, Spain. Veterinary Parasitology. 2009;165: 323–326. doi:10.1016/j.vetpar.2009.07.014

360. Millán J, Candela MG, Vicente Lopez-Bao J, Pereira M, Angeles Jimenez M, Leon-Vizcaino L. Leptospirosis in Wild and Domestic Carnivores in Natural Areas in Andalusia, Spain. Vector-Borne Zoonotic Dis. 2009;9: 549–554. doi:10.1089/vbz.2008.0081

361. Millán J, Casanova JC. Helminth parasites of the endangered Iberian lynx (Lynx pardinus) and sympatric carnivores. Journal of Helminthology. 2007;81: 377–380. doi:10.1017/S0022149X07869203

362. Millán J, Casanova JC. High prevalence of helminth parasites in feral cats in Majorca Island (Spain). Parasitol Res. 2009;106: 183–188. doi:10.1007/s00436-009-1647-y

363. Milstein TC, Goldsmid JM. Parasites of feral cats from southern Tasmania and their potential significance. Aust Vet J. 1997;75: 218–219. doi:10.1111/j.1751-0813.1997.tb10072.x

364. Mircean V, Györke A, Jarca A, Cozma V. Prevalence of Giardia species in stool samples by ELISA in household cats from Romania and risk factors. Journal of Feline Medicine and Surgery. 2011;13: 479–482. doi:10.1016/j.jfms.2011.01.003

365. Mircean V, Titilincu A, Vasile C. Prevalence of endoparasites in household cat (Felis catus) populations from Transylvania (Romania) and association with risk factors. Vet Parasitol. 2010;171: 163–166. doi:10.1016/j.vetpar.2010.03.005

366. Miró G, Montoya A, Jiménez S, Frisuelos C, Mateo M, Fuentes I. Prevalence of antibodies to Toxoplasma gondii and intestinal parasites in stray, farm and household cats in Spain. Veterinary Parasitology. 2004;126: 249–255. doi:10.1016/j.vetpar.2004.08.015

367. Miró G, Rupérez C, Checa R, Gálvez R, Hernández L, García M, et al. Current status of L. infantum infection in stray cats in the Madrid region (Spain): implications for the recent outbreak of human leishmaniosis? Parasites Vectors. 2014;7: 112. doi:10.1186/1756-3305-7-112

368. Modric Z, Bambir S. Leptospirosis in the domestic cat felis domestica briss. in slavonia. Veterinarski Arhiv. 1991;61: 283–288.

369. Mohammadpour I, Bozorg-Ghalati F, Gazzonis AL, Manfredi MT, Motazedian MH, Mohammadpour N. First molecular subtyping and phylogeny of Blastocystis sp. isolated from domestic and synanthropic animals (dogs, cats and brown rats) in southern Iran. Parasites & Vectors. 2020;13: 365. doi:10.1186/s13071-020-04225-9

370. Mohammed OB, Omar OI, Elamin EA, Bushara HO, Omer SA, Alagaili AN. Seroprevalence of Toxoplasma gondii in household and stray cats of Riyadh, Saudi Arabia. Vet Ital. 2019;55: 241–245. doi:10.12834/VetIt.221.695.4

371. Mohd Zain SN, Sahimin N, Pal P, Lewis JW. Macroparasite communities in stray cat populations from urban cities in Peninsular Malaysia. Vet Parasitol. 2013;196: 469–477. doi:10.1016/j.vetpar.2013.03.030

372. Mohebali M, Zarei Z, Khanaliha K, Kia EB, Motavalli-Haghi A, Davoodi J, et al. Intestinal Protozoa in Domestic Cats (Carnivora: Felidae, Felis catus) in Northwestern Iran: A Cross-Sectional Study with Prevalent of Microsporidian and Coccidian Parasites. Iran J Parasitol. 2019;14: 136–142.

373. Monteiro MFM, Ramos RAN, Calado AMC, Lima VFS, Ramos IC do N, Tenório RFL, et al. Gastrointestinal parasites of cats in Brazil: frequency and zoonotic risk. Rev Bras Parasitol Vet. 2016;25: 254–257. doi:10.1590/S1984-29612016019

374. Montoya A, García M, Gálvez R, Checa R, Marino V, Sarquis J, et al. Implications of zoonotic and vector-borne parasites to free-roaming cats in central Spain. Vet Parasitol. 2018;251: 125–130. doi:10.1016/j.vetpar.2018.01.009

375. Morandi B, Greenwood SJ, Conboy GA, Galuppi R, Poglayen G, VanLeeuwen JA. Endoparasites in dogs and cats diagnosed at the Veterinary Teaching Hospital (VTH) of the University of Prince Edward Island between 2000 and 2017. A large-scale retrospective study. Prev Vet Med. 2020;175: 104878. doi:10.1016/j.prevetmed.2019.104878

376. Morelli S, Crisi PE, Di Cesare A, De Santis F, Barlaam A, Santoprete G, et al. Exposure of client-owned cats to zoonotic vector-borne pathogens: Clinic-pathological alterations and infection risk analysis. Comp Immunol Microbiol Infect Dis. 2019;66: 101344. doi:10.1016/j.cimid.2019.101344

377. Moreno G s., Griffiths P l., Connerton I f., Park R w. a. Occurrence of campylobacters in small domestic and laboratory animals. Journal of Applied Bacteriology. 1993;75: 49–54. doi:10.1111/j.1365-2672.1993.tb03406.x

378. Morsy TA, Mohamed S. S, Magda Y. AH. Intestinal parasites of stray cats in Cairo, Egypt. 1981; 331–45.

379. Mosallanejad B, Avizeh R, Jalali MR, Alborzi A. Prevalence of Giardia duodenalis Infection in Household Cats of Ahvaz District, South-West of Iran. Iran J Parasitol. 2010;5: 27–34.

380. Mosallanejad B, Ghorbanpoor M, Avizeh R. A serological survey of Leptospiral infection of cats in Ahvaz, south- western of Iran. International Journal of Veterinary Research. 2011;5: 49–71.

381. Mosallanejad B, Avizeh R, Razi Jalali MH, Pourmehdi M. A study on seroprevalence and coproantigen detection of Toxoplasma gondii in companion cats in Ahvaz area, southwestern Iran. Iranian Journal of Veterinary Research. 2011;12: 139–144. doi:10.22099/ijvr.2011.55

382. Mosallanejad B, Hamidinejat H, Seifiabad Shapouri MR, Rezaei Ghaleh F. A comparison between serological and molecular tests in diagnosis of Toxoplasma gondii infection among stray cats in Ahvaz, southwestern Iran. Archives of Razi Institute. 2017;72: 107–114. doi:10.22092/ari.2017.109841

383. Moser I, Rieksneuwöhner B, Lentzsch P, Schwerk P, Wieler LH. Genomic Heterogeneity and O-Antigenic Diversity ofCampylobacter upsaliensis and Campylobacter helveticus Strains Isolated from Dogs and Cats in Germany. Journal of Clinical Microbiology. 2001;39: 2548–2557. doi:10.1128/jcm.39.7.2548-2557.2001

384. Motta B, Nägeli F, Nägeli C, Solari-Basano F, Schiessl B, Deplazes P, et al. Epidemiology of the eye worm *Thelazia callipaeda* in cats from southern Switzerland. Veterinary Parasitology. 2014;203: 287–293. doi:10.1016/j.vetpar.2014.04.009

385. Moura L, Kelly P, Krecek RC, Dubey JP. Seroprevalence of Toxoplasma gondii in Cats From St. Kitts, West Indies. para. 2007;93: 952–953. doi:10.1645/GE-1195R.1

386. Mugnaini L, Papini R, Gorini G, Passantino A, MERILDI V, Mancianti F. Pattern and predictive factors of endopara- sitism in cats in Central Italy. Revue de médecine vétérinaire. 2012;163: 89–94.

387. Müller A, Walker R, Bittencourt P, Machado RZ, Benevenute JL, Amaral RBD, et al. Prevalence, hematological findings and genetic diversity of Bartonella spp. in domestic cats from Valdivia, Southern Chile. Parasitology. 2017;144: 773–782. doi:10.1017/S003118201600247X

388. Munhoz AD, Hage SB, Cruz RDS, Calazans APF, Silva FL, Albuquerque GR, et al. Toxoplasmosis in cats in northeastern Brazil: Frequency, associated factors and coinfection with *Neospora caninum*, feline immunodeficiency virus and feline leukemia virus. Veterinary Parasitology: Regional Studies and Reports. 2017;8: 35–38. doi:10.1016/j.vprsr.2017.01.007

389. Murillo A, Cuenca R, Serrano E, Marga G, Ahmed A, Cervantes S, et al. Leptospira Detection in Cats in Spain by Serology and Molecular Techniques. International Journal of Environmental Research and Public Health. 2020;17: 1600. doi:10.3390/ijerph17051600

390. Must K, Hytönen MK, Orro T, Lohi H, Jokelainen P. Toxoplasma gondii seroprevalence varies by cat breed. PLOS ONE. 2017;12: e0184659. doi:10.1371/journal.pone.0184659

391. Must K, Lassen B, Jokelainen P. Seroprevalence of and Risk Factors for Toxoplasma gondii Infection in Cats in Estonia. Vector-Borne and Zoonotic Diseases. 2015;15: 597–601. doi:10.1089/vbz.2015.1809

392. Mutinelli F. Rabies and feral cat colonies in Italy. Vet Rec. 2010;166: 537–538. doi:10.1136/vr.c2162

393. Mylonakis ME, Bourtzi-Hatzopoulou E, Koutinas AF, Petridou E, Saridomichelakis MN, Leontides L, et al. Leptospiral seroepidemiology in a feline hospital population in Greece. Vet Rec. 2005;156: 615–616. doi:10.1136/vr.156.19.615

394. Nagamori Y, Payton ME, Duncan-Decocq R, Johnson EM. Fecal survey of parasites in free-roaming cats in northcentral Oklahoma, United States. Veterinary Parasitology: Regional Studies and Reports. 2018;14: 50–53. doi:10.1016/j.vprsr.2018.08.008

395. Nagamori Y, Payton ME, Looper E, Apple H, Johnson EM. Retrospective survey of parasitism identified in feces of client-owned cats in North America from 2007 through 2018. Veterinary Parasitology. 2020;277: 109008. doi:10.1016/j.vetpar.2019.109008

396. Näreaho A, Puomio J, Saarinen K, Jokelainen P, Juselius T, Sukura A. Feline intestinal parasites in Finland: prevalence, risk factors and anthelmintic treatment practices. Journal of Feline Medicine and Surgery. 2012;14: 378–383. doi:10.1177/1098612X12439257

397. Nath TC, Eom KS, Choe S, Islam S, Sabuj SS, Saha E, et al. Insights to helminth infections in food and companion animals in Bangladesh: Occurrence and risk profiling. Parasite Epidemiology and Control. 2022;17: e00245. doi:10.1016/j.parepi.2022.e00245

398. Neto JM, Ferreira FP, Miura AC, Almeida JC de, Martins FDC, Souza M de, et al. An outbreak of caprine toxoplasmosis - investigation and case report. Cienc Rural. 2018;48: e20170790. doi:10.1590/0103-8478cr20170790

399. Neves M, Lopes AP, Martins C, Fino R, Paixão C, Damil L, et al. Survey of Dirofilaria immitis antigen and antibodies to Leishmania infantum and Toxoplasma gondii in cats from Madeira Island, Portugal. Parasit Vectors. 2020;13: 117. doi:10.1186/s13071-020-3988-4

400. Ngui R, Lee S, Yap N, Tan T, Aidil R, Chua K, et al. Gastrointestinal parasites in rural dogs and cats in Selangor and Pahang states in Peninsular Malaysia. Acta Parasitologica. 2014;59: 737–744. doi:10.2478/s11686-014-0306-3

401. Nichol S, Ball SJ, Snow KR. Prevalence of intestinal parasites in feral cats in some urban areas of England. Vet Parasitol. 1981;9: 107–110. doi:10.1016/0304-4017(81)90028-5

402. Nijsse R, Ploeger HW, Wagenaar JA, Mughini-Gras L. Prevalence and risk factors for patent Toxocara infections in cats and cat owners’ attitude towards deworming. Parasitol Res. 2016;115: 4519–4525. doi:10.1007/s00436-016-5242-8

403. Nourollahi Fard SR, Akhtardanesh B, Sadr S, Khedri J, Radfar MH, Shadmehr M. Gastrointestinal helminths infection of free-roaming cats (Felis catus) in Southeast Iran. Veterinary Medicine and Science. 2024;10: e1422. doi:10.1002/vms3.1422

404. Nowotny N. The domestic cat: a possible transmitter of viruses from rodents to man. Lancet. 1994;343: 921. doi:10.1016/s0140-6736(94)90043-4

405. Nutter FB, Dubey JP, Levine JF, Breitschwerdt EB, Ford RB, Stoskopf MK. Seroprevalences of antibodies against Bartonella henselae and Toxoplasma gondii and fecal shedding of Cryptosporidium spp, Giardia spp, and Toxocara cati in feral and pet domestic cats. J Am Vet Med Assoc. 2004;225: 1394–1398. doi:10.2460/javma.2004.225.1394

406. Nyambura Njuguna A, Kagira JM, Muturi Karanja S, Ngotho M, Mutharia L, Wangari Maina N. Prevalence of Toxoplasma gondii and Other Gastrointestinal Parasites in Domestic Cats from Households in Thika Region, Kenya. Biomed Res Int. 2017;2017: 7615810. doi:10.1155/2017/7615810

407. Obrenović S, Radojičič S, Stević N. Seroprevalence of cat Leptospirosis in Belgrade (Serbia). Acta Veterinaria-Beograd. 2014;64: 510–518. doi:12.2478/acve-2014-0047

408. Okaeme AN. Intestinal helminths of cats in the Kainji Lake area Nigeria. Vet Res Commun. 1986;10: 237–240. doi:10.1007/BF02213986

409. Oliveira GMS de, Simões JM, Schaer RE, Freire SM, Nascimento RJM, Pinheiro AMC de M, et al. Frequency and factors associated with *Toxoplasma gondii* infection in pregnant women and their pets in Ilhéus, Bahia, Brazil. Rev Soc Bras Med Trop. 2019;52: e20190250. doi:10.1590/0037-8682-0250-2019

410. Opsteegh M, Haveman R, Swart AN, Mensink-Beerepoot ME, Hofhuis A, Langelaar MFM, et al. Seroprevalence and risk factors for *Toxoplasma gondii* infection in domestic cats in The Netherlands. Preventive Veterinary Medicine. 2012;104: 317–326. doi:10.1016/j.prevetmed.2012.01.003

411. Ortega-Pacheco A, Gutierrez-Blanco E, Cauich-Mendez W, Caacute;rdenas-Marrufo M, Jimenez-Coello M. Leptospira spp. in cats from tropical Mexico. JZD. 2020;4. doi:10.22034/jzd.2020.10583

412. Ortega-Pacheco A, Guzmán-Marín E, Acosta-Viana KY, Vado-Solís I, Jiménez-Delgadillo B, Cárdenas-Marrufo M, et al. Serological survey of Leptospira interrogans, Toxoplasma gondii and Trypanosoma cruzi in free roaming domestic dogs and cats from a marginated rural area of Yucatan Mexico. Veterinary Medicine and Science. 2017;3: 40–47. doi:10.1002/vms3.55

413. Ortuño M, Bernal A, Nachum-Biala Y, Muñoz C, Risueño J, Ortiz J, et al. Clinical, diagnostic and epidemiological implications of Hepatozoon spp., Babesia spp. and Leishmania infantum infection in cats and dogs in a Mediterranean periurban setting. Parasitol Res. 2023;122: 35–47. doi:10.1007/s00436-022-07705-2

414. Otranto D, Napoli E, Latrofa MS, Annoscia G, Tarallo VD, Greco G, et al. Feline and canine leishmaniosis and other vector-borne diseases in the Aeolian Islands: Pathogen and vector circulation in a confined environment. Veterinary Parasitology. 2017;236: 144–151. doi:10.1016/j.vetpar.2017.01.019

415. Overgaauw PAM, van Zutphen L, Hoek D, Yaya FO, Roelfsema J, Pinelli E, et al. Zoonotic parasites in fecal samples and fur from dogs and cats in The Netherlands. Veterinary Parasitology. 2009;163: 115–122. doi:10.1016/j.vetpar.2009.03.044

416. Palerme J-S, Lamperelli E, Gagne J, Cazlan C, Zhang M, Olds JE. Seroprevalence of Leptospira spp., Toxoplasma gondii, and Dirofilaria immitis in Free-Roaming Cats in Iowa. Vector Borne Zoonotic Dis. 2019;19: 193–198. doi:10.1089/vbz.2017.2255

417. Palmer CS, Thompson RCA, Traub RJ, Rees R, Robertson ID. National study of the gastrointestinal parasites of dogs and cats in Australia. Veterinary Parasitology. 2008;151: 181–190. doi:10.1016/j.vetpar.2007.10.015

418. Palmer JP, Gazêta G, André M, Coelho A, Corrêa L, Damasceno J, et al. Piroplasm Infection in Domestic Cats in the Mountainous Region of Rio de Janeiro, Brazil. Pathogens. 2022;11: 900. doi:10.3390/pathogens11080900

419. Pan W, Wang M, Abdullahi AY, Fu Y, Yan X, Yang F, et al. Prevalence and genotypes of *Giardia lamblia* from stray dogs and cats in Guangdong, China. Veterinary Parasitology: Regional Studies and Reports. 2018;13: 30–34. doi:10.1016/j.vprsr.2018.03.012

420. Panait LC, Ionică AM, Cazan CD, Coroian M, Diacu AM, Boncea AM, et al. Apicomplexan haemoparasites in domestic cats in Romania. Parasites & Vectors. 2023;16: 56. doi:10.1186/s13071-023-05683-7

421. Paoletti B, Otranto D, Weigl S, Giangaspero A, Cesare AD, Traversa D. Prevalence and genetic characterization of *Giardia* and *Cryptosporidium* in cats from Italy. Research in Veterinary Science. 2011;91: 397–399. doi:10.1016/j.rvsc.2010.09.011

422. Park H-J, Lee S-E, Hong S-H, Lee W-J, Seo K-W, Song K-H. Seroprevalence of *Toxoplasma gondii* and Bartonella henselase infection in stray cats of the Daejeon City, Korea. Korean Journal of Veterinary Research. 2014;54: 87–89. doi:10.14405/kjvr.2014.54.2.87

423. Parreira I, Jayme B, Buzin E, Tomaz L. Epidemiological features of infection through Leptospira spp. in domestic cats (felis catus) apparently healthy within the metropolitan area of Goiania, Brazil. Enciclopedia Biosfera. 2010;60. Available: https://conhecer.org.br/ojs/index.php/biosfera/article/view/4748

424. Pavlova EV, Kirilyuk EV, Naidenko SV. Occurrence Pattern of Influenza A Virus, Coxiella burnetii, Toxoplasma gondii, and Trichinella sp. in the Pallas Cat and Domestic Cat and Their Potential Prey Under Arid Climate Conditions. Arid Ecosyst. 2016;6: 277–283. doi:10.1134/S2079096116040089

425. Pena HFJ, Soares RM, Amaku M, Dubey JP, Gennari SM. *Toxoplasma gondii* infection in cats from São Paulo state, Brazil: Seroprevalence, oocyst shedding, isolation in mice, and biologic and molecular characterization. Research in Veterinary Science. 2006;81: 58–67. doi:10.1016/j.rvsc.2005.09.007

426. Peña-Quistial MG, Benavides-Montaño JA, Duque NJR, Benavides-Montaño GA. Prevalence and associated risk factors of Intestinal parasites in rural high-mountain communities of the Valle del Cauca—Colombia. PLOS Neglected Tropical Diseases. 2020;14: e0008734. doi:10.1371/journal.pntd.0008734

427. Pereira PF, Barbosa A da S, Santos ALC, Bolais PF, Dardé M-L, Amendoeira MRR. *Toxoplasma gondii*: infection among shelter and stray cats in Rio de Janeiro, Brazil. Rev Bras Parasitol Vet. 2018;27: 401–408. doi:10.1590/S1984-296120180061

428. Perera SC, Capella G de A, Pinto NB, Rappeti JC da S, Müller G, Azambuja RHM, et al. First isolation of Dioctophyme renale eggs from an urban environment and identification of those from animal urine. Rev Bras Parasitol Vet. 2017;26: 89–91. doi:10.1590/S1984-29612016064

429. Persichetti M-F, Solano-Gallego L, Serrano L, Altet L, Reale S, Masucci M, et al. Detection of vector-borne pathogens in cats and their ectoparasites in southern Italy. Parasites Vectors. 2016;9: 247. doi:10.1186/s13071-016-1534-1

430. Petavy AF, Tenora F, Deblock S, Sergent V. Echinococcus multilocularis in domestic cats in France. A potential risk factor for alveolar hydatid disease contamination in humans. Vet Parasitol. 2000;87: 151–156. doi:10.1016/s0304-4017(99)00181-8

431. Piekara-Stępińska A, Piekarska J, Gorczykowski M, Bania J. Genotypes of Giardia duodenalis in Household Dogs and Cats from Poland. Acta Parasit. 2021;66: 428–435. doi:10.1007/s11686-020-00292-1

432. Pinto LD, Araujo FAP de, Stobb NS, Marques SMT. Soroepidemiologia de Toxoplasma gondii em gatos domiciliados atendidos em clínicas particulares de Porto Alegre, RS, Brasil. Cienc Rural. 2009;39: 2464–2469. doi:10.1590/S0103-84782009005000185

433. Pinto Luís Brucinski G, Alvares Santarém V, Ribeiro J, Teixeira de Souza Filho R, Alves de França D, Nunes de Moraes G, et al. Toxoplasmosis Behind Bars: One Health Approach on Serosurvey Dynamics and Associated Risk Factors for Women Inmates, Correctional Officers, and In-Prison Feral Cats. Transboundary and Emerging Diseases. 2024;2024: e9390381. doi:10.1155/2024/9390381

434. Poglayen G, Traldi G, Capelli G, Genchi C. [Gastrointestinal parasitic fauna of cats in the cities of Bologna, Florence and Milan]. Parassitologia. 1985;27: 297–302.

435. Pomroy WE. A survey of helminth parasites of cats from Saskatoon. Can Vet J. 1999;40: 339–340.

436. Potes-Morales C, Crespo-Ortiz M del P. Molecular diagnosis of intestinal protozoa in young adults and their pets in Colombia, South America. PLOS ONE. 2023;18: e0283824. doi:10.1371/journal.pone.0283824

437. Pratt N, Conan A, Rajeev S. Leptospira Seroprevalence in Domestic Dogs and Cats on the Caribbean Island of Saint Kitts. Vet Med Int. 2017;2017: 5904757. doi:10.1155/2017/5904757

438. Qian W, Wang H, Su C, Shan D, Cui X, Yang N, et al. Isolation and characterization of *Toxoplasma gondii* strains from stray cats revealed a single genotype in Beijing, China. Veterinary Parasitology. 2012;187: 408–413. doi:10.1016/j.vetpar.2012.01.026

439. Qiu H-Y, Zhang X-X, Jiang J, Cai Y, Xu P, Zhao Q, et al. Toxoplasma gondii Seropositivity and Associated Risk Factors in Cats (Felis catus) in Three Provinces in Northeastern China from 2013 to 2019. Vector Borne Zoonotic Dis. 2020;20: 723–727. doi:10.1089/vbz.2019.2583

440. Queen E v., Marks S l., Farver T b. Prevalence of Selected Bacterial and Parasitic Agents in Feces from Diarrheic and Healthy Control Cats from Northern California. Journal of Veterinary Internal Medicine. 2012;26: 54–60. doi:10.1111/j.1939-1676.2011.00843.x

441. Qurollo BA, Balakrishnan N, Cannon CZ, Maggi RG, Breitschwerdt EB. Co-infection with Anaplasma platys, Bartonella henselae, Bartonella koehlerae and “Candidatus Mycoplasma haemominutum” in a cat diagnosed with splenic plasmacytosis and multiple myeloma. J Feline Med Surg. 2014;16: 713–720. doi:10.1177/1098612X13519632

442. Raab O, Greenwood S, Vanderstichel R, Gelens H. A cross-sectional study of Tritrichomonas foetus infection in feral and shelter cats in Prince Edward Island, Canada. Can Vet J. 2016;57: 265–270.

443. Rabbani IA-R, Mareta FJ, Kusnoto, Hastutiek P, Lastuti NDR, Mufasirin, et al. Zoonotic and other gastrointestinal parasites in cats in Lumajang, East Java, Indonesia. Infect Dis Rep. 2020;12: 8747. doi:10.4081/idr.2020.8747

444. Rambozzi L, Menzano A, Mannelli A, Romano S, Isaia MC. Prevalence of cryptosporidian infection in cats in Turin and analysis of risk factors. Journal of Feline Medicine and Surgery. 2007;9: 392–396. doi:10.1016/j.jfms.2007.03.005

445. Ramírez ML, Sánchez Vargas G, Vielma Sandoval M, Soto Mancilla JL. Presence of anti-Toxoplasma antibodies in humans and their cats in the urban zone of Guadalajara. Rev Soc Bras Med Trop. 1999;32: 483–488. doi:10.1590/s0037-86821999000500003

446. Ramos DG de S, Scheremeta RGA da C, Oliveira ACS de, Sinkoc AL, Pacheco R de C. Survey of helminth parasites of cats from the metropolitan area of Cuiabá, Mato Grosso, Brazil. Rev Bras Parasitol Vet. 2013;22: 201–206. doi:10.1590/S1984-29612013000200040

447. Ramos N de V, Silva MLE, Barreto MS, Barros LA, Mendes-de-Almeida F. Endoparasites of household and shelter cats in the city of Rio de Janeiro, Brazil. Rev Bras Parasitol Vet. 2020;29: e012819. doi:10.1590/S1984-29612019110

448. Ravicini S, Pastor J, Hawley J, Brewer M, Castro-López J, Beall M, et al. Prevalence of selected infectious disease agents in stray cats in Catalonia, Spain. JFMS open reports. 2016;2. doi:10.1177/2055116916634109

449. Rawangchue T, Sripirom N, Sungpradit S. Surveillance of zoonotic Brugia pahangi in monastery cats, Samphran district, Nakhon Pathom, Thailand. The Thai Journal of Veterinary Medicine. 2022;52: 117–125. doi:10.56808/2985-1130.3196

450. Rembiesa C, Richardson DJ. Helminth Parasites of the House Cat, Felis catus, in Connecticut, U.S.A. copa. 2003;70: 115–119. doi:10.1654/1525-2647(2003)070%5B0115:HPOTHC%5D2.0.CO;2

451. Remesar S, Arnal JL, Gómez A, Prieto A, García-Dios D, Benito A, et al. A case report of fatal feline babesiosis caused by Babesia canis in north western Spain. BMC Veterinary Research. 2022;18: 177. doi:10.1186/s12917-022-03287-4

452. Rengifo-Herrera C, Pile E, García A, Pérez A, Pérez D, Nguyen FK, et al. Seroprevalence of Toxoplasma gondii in domestic pets from metropolitan regions of Panama. Parasite. 2017;24: 9. doi:10.1051/parasite/2017009

453. Reyes MF, Guevara VG, Roque DGDS, Flores MLS, Lastica EA. Seroprevalence of Toxoplasma gondii antibodies in domestic short-haired cats (Felis catus) in a wildlife facility in Manila. Philippine Journal of Veterinary and Animal Sciences. 2013;39. Available: https://www.pjvas.org/index.php/pjvas/article/view/32

454. Ribeiro TMP, Reis TS, Nogueira AFS, Sousa SAP, Gomes FA, Paludo GR, et al. Antibody prevalence for Leptospira spp. and Brucella abortus in domestic cats from Araguaína, Tocantins, North Region of Brazil. Revista Brasileira de Ciência Veterinária. 2021;28. Available: https://periodicos.uff.br/rbcv/article/view/50353

455. Rico-Torres CP, Del Viento-Camacho A, Caballero-Ortega H, Besné-Mérida A, Luna-Pastén H, Correa D, et al. First isolation of *Toxoplasma gondii* from cats of Colima, Mexico: Tissue distribution and genetic characterization. Veterinary Parasitology. 2015;209: 125–128. doi:10.1016/j.vetpar.2015.02.004

456. Ridwan Y, Sudarnika E, Dewi TIT, Budiono NG. Gastrointestinal helminth parasites of pets: Retrospective study at the veterinary teaching hospital, IPB University, Bogor, Indonesia. Vet World. 2023;16: 1043–1051. doi:10.14202/vetworld.2023.1043-1051

457. Riggio F, Mannella R, Ariti G, Perrucci S. Intestinal and lung parasites in owned dogs and cats from central Italy. Veterinary Parasitology. 2013;193: 78–84. doi:10.1016/j.vetpar.2012.11.026

458. Rocha AVVO, Moreno BFS, Cabral AD, Louzeiro NM, Miranda LM, Santos VMB dos, et al. Diagnosis and epidemiology of Leishmania infantum in domestic cats in an endemic area of the Amazon region, Brazil. Veterinary Parasitology. 2019;273: 80–85. doi:10.1016/j.vetpar.2019.08.007

459. Rodriguez J, Blais M-C, Lapointe C, Arsenault J, Carioto L, Harel J. Serologic and Urinary PCR Survey of Leptospirosis in Healthy Cats and in Cats with Kidney Disease. Journal of Veterinary Internal Medicine. 2014;28: 284–293. doi:10.1111/jvim.12287

460. Rodríguez-Ponce E, González JF, Conde de Felipe M, Hernández JN, Raduan Jaber J. Epidemiological survey of zoonotic helminths in feral cats in Gran Canaria island (Macaronesian archipelago-Spain). Acta Parasitol. 2016;61: 443–450. doi:10.1515/ap-2016-0059

461. Rojekittikhun W, Chaisiri K, Mahittikorn A, Pubampen S, Sa-Nguankiat S, Kusolsuk T, et al. Gastrointestinal parasites of dogs and cats in a refuge in Nakhon Nayok, Thailand. Southeast Asian J Trop Med Public Health. 2014;45: 31–39.

462. Rollag OJ, Skeels MR, Nims LJ, Thilsted JP, Mann JM. Feline plague in New Mexico: report of five cases. J Am Vet Med Assoc. 1981;179: 1381–1383.

463. Roqueplo C, Cabre O, Davoust B, Kodjo A. Epidemiological Study of Animal Leptospirosis in New Caledonia. Veterinary Medicine International. 2013;2013: e826834. doi:10.1155/2013/826834

464. Rosa LD, Moura AB de, Trevisani N, Medeiros AP, Sartor AA, Souza AP de, et al. Toxoplasma gondii antibodies on domiciled cats from Lages municipality, Santa Catarina State, Brazil. Rev Bras Parasitol Vet. 2010;19: 268–269. doi:10.1590/S1984-29612010000400017

465. Rose L. Prevalence of antibodies and clinical suspected cases of leptospirosis in cats in the Berlin/Brandenburg area. Berliner und Münchener Tierärztliche Wochenschrift,. 2019;132: 140–147.

466. Rossi M, Hänninen ML, Revez J, Hannula M, Zanoni RG. Occurrence and species level diagnostics of *Campylobacter* spp., enteric *Helicobacter* spp. and *Anaerobiospirillum* spp. in healthy and diarrheic dogs and cats. Veterinary Microbiology. 2008;129: 304–314. doi:10.1016/j.vetmic.2007.11.014

467. Roura X, Peters IR, Altet L, Tabar M-D, Barker EN, Planellas M, et al. Prevalence of hemotropic mycoplasmas in healthy and unhealthy cats and dogs in Spain. J Vet Diagn Invest. 2010;22: 270–274. doi:10.1177/104063871002200219

468. Ruaux CG, Stang BV. Prevalence of Blastocystis in Shelter-Resident and Client-Owned Companion Animals in the US Pacific Northwest. PLOS ONE. 2014;9: e107496. doi:10.1371/journal.pone.0107496

469. Ryan GE. Gastro-Intestinal Parasites of Feral Cats in New South Wales. Australian Veterinary Journal. 2008;52: 224–227. doi:10.1111/j.1751-0813.1976.tb00072.x

470. Sacristán I, Sieg M, Acuña F, Aguilar E, García S, López MJ, et al. Molecular and serological survey of carnivore pathogens in free-roaming domestic cats of rural communities in southern Chile. J Vet Med Sci. 2019;81: 1740–1748. doi:10.1292/jvms.19-0208

471. Sadjjadi S, Oryan A, AR J, Mehrabani D. Prevalence and intensity of infestation with Toxocara cati stray cats in Shiraz Iran. VETERINARY ARHIV. 2001;71: 149–157.

472. Sævik BK, Krontveit RI, Eggen KP, Malmberg N, Thoresen SI, Prestrud KW. Toxoplasma gondii seroprevalence in pet cats in Norway and risk factors for seropositivity. Journal of Feline Medicine and Surgery. 2015;17: 1049–1056. doi:10.1177/1098612X15569616

473. Salakij C, Lertwatcharasarakul P, Salakij J, Nunklang K, Rattanakunuprakarn J. Molecular characterization of Anaplasma platys in a domestic cat from Thailand. Comp Clin Pathol. 2012;21: 345–348. doi:10.1007/s00580-011-1378-1

474. Salgado-Cardoso AM, Olave-Leyva JI, Morales I, Setién AA, López-Martínez I, Ceballos NA. Molecular Epidemiology and Spatial Distribution of Cat Rabies Cases in Yucatan, Mexico. Preprints; 2024. doi:10.20944/preprints202405.1140.v1

475. Salman D, Pumidonming W, Oohashi E, Igarashi M. Prevalence of *Toxoplasma gondii* and other intestinal parasites in cats in Tokachi sub-prefecture, Japan. Journal of Veterinary Medical Science. 2018;80: 960–967. doi:10.1292/jvms.17-0713

476. Sandberg M, Bergsjø B, Hofshagen M, Skjerve E, Kruse H. Risk factors for *Campylobacter* infection in Norwegian cats and dogs. Preventive Veterinary Medicine. 2002;55: 241–253. doi:10.1016/S0167-5877(02)00095-8

477. Sander A, Bühler C, Pelz K, von Cramm E, Bredt W. Detection and identification of two Bartonella henselae variants in domestic cats in Germany. J Clin Microbiol. 1997;35: 584–587.

478. Santín M, Trout JM, Vecino JAC, Dubey JP, Fayer R. *Cryptosporidium*, *Giardia* and *Enterocytozoon bieneusi* in cats from Bogota (Colombia) and genotyping of isolates. Veterinary Parasitology. 2006;141: 334–339. doi:10.1016/j.vetpar.2006.06.004

479. Sato S, Kabeya H, Negishi A, Tsujimoto H, Nishigaki K, Endo Y, et al. Molecular survey of Bartonella henselae and Bartonella clarridgeiae in pet cats across Japan by species-specific nested-PCR. Epidemiol Infect. 2017;145: 2694–2700. doi:10.1017/S0950268817001601

480. Sauda F, Malandrucco L, De Liberato C, Perrucci S. Gastrointestinal parasites in shelter cats of central Italy. Vet Parasitol Reg Stud Reports. 2019;18: 100321. doi:10.1016/j.vprsr.2019.100321

481. Savani ESMM, de Oliveira Camargo MCG, de Carvalho MR, Zampieri RA, dos Santos MG, D’Auria SRN, et al. The first record in the Americas of an autochthonous case of Leishmania (Leishmania) infantum chagasi in a domestic cat (Felix catus) from Cotia County, São Paulo State, Brazil. Vet Parasitol. 2004;120: 229–233. doi:10.1016/j.vetpar.2004.01.008

482. Savidge C, Ewing P, Andrews J, Aucoin D, Lappin MR, Moroff S. Anaplasma phagocytophilum infection of domestic cats: 16 cases from the northeastern USA. Journal of Feline Medicine and Surgery. 2016;18: 85–91. doi:10.1177/1098612X15571148

483. Schäfer I, Peukert A, Kerner K, Müller E. Vector-Borne Pathogens in Stray Cats in Eastern Germany (Thuringia). Animals. 2023;13: 2574. doi:10.3390/ani13162574

484. Schreiber N, Basso W, Riond B, Willi B, Torgerson PR, Deplazes P. Antibody kinetics and exposure to *Toxoplasma gondii* in cats: a seroepidemiological study. International Journal for Parasitology. 2021;51: 291–299. doi:10.1016/j.ijpara.2020.09.011

485. Scorza AV, Duncan C, Miles L, Lappin MR. Prevalence of selected zoonotic and vector-borne agents in dogs and cats in Costa Rica. Veterinary Parasitology. 2011;183: 178–183. doi:10.1016/j.vetpar.2011.06.025

486. Sebastian JF, Reagan KL, Peavy T, Zecca IB, Hamer SA, Sykes JE. Evaluation of Leptospira infection and exposure in free-roaming cat populations in northern California and southern Texas. J Feline Med Surg. 2023;25: 1098612X231162471. doi:10.1177/1098612X231162471

487. Sharif M, Nasrolahei M, Ziapour SP, Gholami S, Ziaei H, Daryani A, et al. Toxocara cati infections in stray cats in northern Iran. Journal of Helminthology. 2007;81: 63–66. doi:10.1017/S0022149X07214117

488. Sharif M, Daryani A, Nasrolahei M, Ziapour SP. Prevalence of Toxoplasma gondii antibodies in stray cats in Sari, northern Iran. Trop Anim Health Prod. 2009;41: 183–187. doi:10.1007/s11250-008-9173-y

489. Shaw SE, Binns SH, Birtles RJ, Day MJ, Smithson RC, Kenny MJ. Molecular evidence of tick‐transmitted infections in dogs and cats in the United Kingdom. Veterinary Record. 2005;157: 645–648. doi:10.1136/vr.157.21.645

490. Sherry K, Miró G, Trotta M, Miranda C, Montoya A, Espinosa C, et al. A serological and molecular study of Leishmania infantum infection in cats from the Island of Ibiza (Spain). Vector Borne Zoonotic Dis. 2011;11: 239–245. doi:10.1089/vbz.2009.0251

491. Shin S-S, Oh D-S, Ahn K-S, Cho S-H, Lee W-J, Na B-K, et al. Zoonotic Intestinal Trematodes in Stray Cats (*Felis catus*) from Riverside Areas of the Republic of Korea. Korean J Parasito. 2015;53: 209–213. doi:10.3347/kjp.2015.53.2.209

492. Shoshi Y, Kazato K, Maeda T, Takashima Y, Watari Y, Matsumoto Y, et al. Prevalence of serum antibodies to Toxoplasma gondii in free-ranging cats on Tokunoshima Island, Japan. J Vet Med Sci. 2021;83: 333–337. doi:10.1292/jvms.20-0512

493. Shukla R, Giraldo P, Kraliz A, Finnigan M, Sanchez AL. Cryptosporidium spp. and other zoonotic enteric parasites in a sample of domestic dogs and cats in the Niagara region of Ontario. Can Vet J. 2006;47: 1179–1184.

494. Shuralev EA, Shamaev ND, Mukminov MN, Nagamune K, Taniguchi Y, Saito T, et al. *Toxoplasma gondii* seroprevalence in goats, cats and humans in Russia. Parasitology International. 2018;67: 112–114. doi:10.1016/j.parint.2017.10.014

495. Silaghi C, Knaus M, Rapti D, Kusi I, Shukullari E, Hamel D, et al. Survey of Toxoplasma gondii and Neospora caninum, haemotropic mycoplasmas and other arthropod-borne pathogens in cats from Albania. Parasites & Vectors. 2014;7: 62. doi:10.1186/1756-3305-7-62

496. Sillman SJ, Drozd M, Loy D, Harris SP. Naturally occurring highly pathogenic avian influenza virus H5N1 clade 2.3.4.4b infection in three domestic cats in North America during 2023. Journal of Comparative Pathology. 2023;205: 17–23. doi:10.1016/j.jcpa.2023.07.001

497. Silva ALP, Lima EF, Silva Filho GM, Ferreira LC, Campos B de A, Bison I, et al. Seroepidemiological Survey of Anti-Toxoplasma gondii and Anti-Neospora caninum Antibodies in Domestic Cats (Felis catus) in Rolim de Moura, State of Rondônia, North Brazil. Tropical Medicine and Infectious Disease. 2023;8: 220. doi:10.3390/tropicalmed8040220

498. Silva JCR, Gennari SM, Ragozo AMA, Amajones VR, Magnabosco C, Yai LEO, et al. Prevalence of Toxoplasma gondii Antibodies in Sera of Domestic Cats From Guarulhos and São Paulo, Brazil. para. 2002;88: 419–420. doi:10.1645/0022-3395(2002)088%5B0419:POTGAI%5D2.0.CO;2

499. Silva JCR, Marvulo MFV, Ferreira F, Dias RA, Ferreira Neto JS, Heinemann MB, et al. Seroepidemiological investigation of animal leptospirosis and molecular characterization of the first Leptospira strain isolated from Fernando de Noronha archipelago, Brazil. Transboundary and Emerging Diseases. 2021;68: 2477–2488. doi:10.1111/tbed.13915

500. Silva WI, Lima EF, Silva JO, Alves M de M, Alves CLP, Silva ALP, et al. Endoparasites in domestic cats (Felis catus) in the semi-arid region of Northeast Brazil. Rev Bras Parasitol Vet. 2023;32: e012123. doi:10.1590/S1984-29612023065

501. Silva YH da, Campos DR, Lima GAC, Quintal JP, Guimarães BG, Rêgo GMM do, et al. Prevalence of gastrointestinal parasites in domestic cats (*Felis catus*) diagnosed by different coproparasitological techniques in the municipality of Seropédica, Rio de Janeiro. Rev Bras Parasitol Vet. 2023;32: e006223. doi:https://doi.org/10.1590/S1984-29612023049

502. Smith KE, Zimmerman JJ, Patton S, Beran GW, Hill HT. The epidemiology of toxoplasmosis on Iowa swine farms with an emphasis on the roles of free-living mammals. Vet Parasitol. 1992;42: 199–211. doi:10.1016/0304-4017(92)90062-e

503. Sobrinho LSV, Rossi CN, Vides JP, Braga ET, Gomes AAD, de Lima VMF, et al. Coinfection of *Leishmania chagasi* with *Toxoplasma gondii*, Feline Immunodeficiency Virus (FIV) and Feline Leukemia Virus (FeLV) in cats from an endemic area of zoonotic visceral leishmaniasis. Veterinary Parasitology. 2012;187: 302–306. doi:10.1016/j.vetpar.2012.01.010

504. Soe BK, Hlaing KS, Naing TW, Thaw ZH, Myint and W. The first study on the prevalence of gastrointestinal parasites in owned and sheltered cats in Yangon, Myanmar. Vet World. 2023;16: 414–420. doi:10.14202/vetworld.2023.414-420

505. Solano-Barquero A, Estrada A, Medaglia A, Montenegro VM, Rojas A. Emerging *Lagochilascaris minor* infections in domestic cats from Costa Rica: A zoonotic threat for the region. Veterinary Parasitology: Regional Studies and Reports. 2022;36: 100797. doi:10.1016/j.vprsr.2022.100797

506. Solano-Gallego L, Hegarty B, Espada Y, Llull J, Breitschwerdt E. Serological and molecular evidence of exposure to arthropod-borne organisms in cats from northeastern Spain. Vet Microbiol. 2006;118: 274–277. doi:10.1016/j.vetmic.2006.07.010

507. Solomatina M, Bespalova N. Features of epizootology of toxoplasmosis of cats in the city of Lipetsk. International Student Scientific Bulletin. 2018;4.

508. Sommer MF, Rupp P, Pietsch M, Kaspar A, Beelitz P. *Giardia* in a selected population of dogs and cats in Germany – diagnostics, coinfections and assemblages. Veterinary Parasitology. 2018;249: 49–56. doi:10.1016/j.vetpar.2017.11.006

509. Sousa KCMD, Herrera HM, Domingos IH, Campos JBV, Santos IMCD, Neves HH, et al. Serological detection of Toxoplasma gondii, Leishmania infantum and Neospora caninum in cats from an area endemic for leishmaniasis in Brazil. Rev Bras Parasitol Vet. 2014;23: 449–455. doi:10.1590/s1984-29612014078

510. Souza JBB, Silva ZM de A, Alves-Ribeiro BS, Moraes I de S, Alves-Sobrinho AV, Saturnino KC, et al. Prevalence of Intestinal Parasites, Risk Factors and Zoonotic Aspects in Dog and Cat Populations from Goiás, Brazil. Veterinary Sciences. 2023;10: 492. doi:10.3390/vetsci10080492

511. Souza LL de, Nascente P da S, Nobre MO, Meinerz ARM, Meireles MCA. Isolation of Sporothrix schenkii from the nails of healthy cats. Braz J Microbiol. 2006;37: 372–374. doi:10.1590/S1517-83822006000300031

512. Souza SFD, Medeiros LDS, Belfort ADS, Cordeiro ALL, Federle M, Souza APD, et al. Anticorpos anti-toxoplasma gondii em gatos domiciliados no Município de Rio Branco, Acre, Brasil. Sem Ci Agr. 2015;36: 3757. doi:10.5433/1679-0359.2015v36n6p3757

513. Spada E, Canzi I, Baggiani L, Perego R, Vitale F, Migliazzo A, et al. Prevalence of Leishmania infantum and co-infections in stray cats in northern Italy. Comp Immunol Microbiol Infect Dis. 2016;45: 53–58. doi:10.1016/j.cimid.2016.03.001

514. Spada E, Canzi I, Baggiani L, Perego R, Vitale F, Migliazzo A, et al. Prevalence of *Leishmania infantum* and co-infections in stray cats in northern Italy. Comparative Immunology, Microbiology and Infectious Diseases. 2016;45: 53–58. doi:10.1016/j.cimid.2016.03.001

515. Spada E, Proverbio D, Della Pepa A, Domenichini G, Bagnagatti De Giorgi G, Traldi G, et al. Prevalence of faecal-borne parasites in colony stray cats in northern Italy. Journal of Feline Medicine and Surgery. 2013;15: 672–677. doi:10.1177/1098612X12473467

516. Spada E, Proverbio D, Galluzzo P, Della Pepa A, Bagnagatti De Giorgi G, Perego R, et al. Prevalence of haemoplasma infections in stray cats in northern Italy. ISRN Microbiol. 2014;2014: 298352. doi:10.1155/2014/298352

517. Spada E, Proverbio D, Pepa A della, Perego R, Baggiani L, DeGiorgi GB, et al. Seroprevalence of feline immunodeficiency virus, feline leukaemia virus and Toxoplasma gondii in stray cat colonies in northern Italy and correlation with clinical and laboratory data. Journal of Feline Medicine and Surgery. 2012;14: 369–377. doi:10.1177/1098612X12437352

518. Spain CV, Scarlett JM, Wade SE, McDonough P. Prevalence of enteric zoonotic agents in cats less than 1 year old in central New York State. J Vet Intern Med. 2001;15: 33–38. doi:10.1892/0891-6640(2001)015%3C0033:poezai%3E2.3.co;2

519. Sprißler F, Jongwattanapisan P, Luengyosluechakul S, Pusoonthornthum R, Prapasarakul N, Kurilung A, et al. Leptospira infection and shedding in cats in Thailand. Transboundary and Emerging Diseases. 2019;66: 948–956. doi:10.1111/tbed.13110

520. Srisanyong W, Takhampunya R, Boonmars T, Kerdsin A, Suksawat F. Prevalence of Bartonella henselae, Bartonella clarridgeiae, and Bartonella vinsonii subsp. berkhoffii in pet cats from four provincial communities in Thailand. The Thai Journal of Veterinary Medicine. 2016;46: 663–670. doi:10.56808/2985-1130.2786

521. Sroka J, Karamon J, Dutkiewicz J, Wójcik Fatla A, Zając V, Cencek T. Prevalence of Toxoplasma gondii infection in cats in southwestern Poland. Ann Agric Environ Med. 2018;25: 576–580. doi:10.26444/aaem/94675

522. Sroka J, Szymańska J. Analysis of Prevalence of Toxoplasma Gondii Infection in Selected Rural Households in the Lublin Region. Bulletin of the Veterinary Institute in Pulawy. 2012;56: 529–534. doi:10.2478/v10213-012-0093-2

523. Stepanić M, Duvnjak S, Reil I, Hađina S, Kempf VAJ, Špičić S, et al. Epidemiology of Bartonella henselae infection in pet and stray cats in Croatia with risk factors analysis. Parasit Vectors. 2024;17: 48. doi:10.1186/s13071-024-06117-8

524. Stoichev I, Sherkov Sh, Halacheva M. Pathology of cats from a region of Bulgaria with human endemic nephropathy. Journal of Comparative Pathology. 1982;92: 99–107. doi:10.1016/0021-9975(82)90045-7

525. Stojanovic V, Foley P. Infectious disease prevalence in a feral cat population on Prince Edward Island, Canada. Can Vet J. 2011;52: 979–982.

526. Subrata IM, Oka IBM, Agustina KK. Prevalence of Intestinal Worm in Free Ranging Domestic Cats in Bali (Prevalensi Cacing Usus Pada Kucing Peliharaan Yang Bebas Berkeliaran Di Bali). JVet. 2017;18: 441. doi:10.19087/jveteriner.2017.18.3.441

527. Subrata M, Astawa N, Suryadi N, Purnama S, Agustina K, Harjana N, et al. The Seroprevalence of Toxoplasma gondii in Cats at the House of Maternal Women with Toxoplasmosis in Badung, Indonesia. Kesmas. 2021;16: 271–278. doi:10.21109/kesmas.v16i4.4954

528. Sudasinghe T, Rajapakse RPVJ, Perera N a. ND, Kumarasiri PVR, Eriyagama NB, Arseculeratne SN. The regional sero-epidemiology of rhinosporidiosis in Sri Lankan humans and animals. Acta Trop. 2011;120: 72–81. doi:10.1016/j.actatropica.2011.06.016

529. Sukhumavasi W, Bellosa ML, Lucio-Forster A, Liotta JL, Lee ACY, Pornmingmas P, et al. Serological survey of *Toxoplasma gondii*, *Dirofilaria immitis*, Feline Immunodeficiency Virus (FIV) and Feline Leukemia Virus (FeLV) infections in pet cats in Bangkok and vicinities, Thailand. Veterinary Parasitology. 2012;188: 25–30. doi:10.1016/j.vetpar.2012.02.021

530. Suzán G, Ceballos G. The role of feral mammals on wildlife infectious disease prevalence in two nature reserves within Mexico City limits. zamd. 2005;36: 479–484. doi:10.1638/04-078.1

531. Svobodová V, Svoboda M. [Incidence of Toxoplasma gondii oocysts in cat feces]. Vet Med (Praha). 1986;31: 621–628.

532. Switzer AD, McMillan-Cole AC, Kasten RW, Stuckey MJ, Kass PH, Chomel BB. Bartonella and Toxoplasma Infections in Stray Cats from Iraq. Am J Trop Med Hyg. 2013;89: 1219–1224. doi:10.4269/ajtmh.13-0353

533. Sykes JE, Terry JC, Lindsay LL, Owens SD. Prevalences of various hemoplasma species among cats in the United States with possible hemoplasmosis. J Am Vet Med Assoc. 2008;232: 372–379. doi:10.2460/javma.232.3.372

534. Symeonidou I, Gelasakis AI, Arsenopoulos K, Angelou A, Beugnet F, Papadopoulos E. Feline gastrointestinal parasitism in Greece: emergent zoonotic species and associated risk factors. Parasites Vectors. 2018;11: 227. doi:10.1186/s13071-018-2812-x

535. Szwabe K, Błaszkowska J. Stray dogs and cats as potential sources of soil contamination with zoonotic parasites. Annals of Agricultural and Environmental Medicine. 2017;24. doi:10.5604/12321966.1234003

536. Taetzsch SJ, Gruszynski KR, Bertke AS, Dubey JP, Monti KA, Zajac AM, et al. Prevalence of zoonotic parasites in feral cats of Central Virginia, USA. Zoonoses Public Health. 2018;65: 728–735. doi:10.1111/zph.12488

537. Tagwireyi WM, Etter E, Neves L. Seroprevalence and associated risk factors of Toxoplasma gondii infection in domestic animals in southeastern South Africa. Onderstepoort Journal of Veterinary Research. 2019;86: 6. doi:10.4102/ojvr.v86i1.1688

538. Takeuchi-Storm N, Mejer H, Al-Sabi MNS, Olsen CS, Thamsborg SM, Enemark HL. Gastrointestinal parasites of cats in Denmark assessed by necropsy and concentration McMaster technique. Veterinary Parasitology. 2015;214: 327–332. doi:10.1016/j.vetpar.2015.06.033

539. Talebkhan Garoussi M, Mehravaran M, Abdollahpour G, Khoshnegah J. Seroprevalence of leptospiral infection in feline population in urban and dairy cattle herds in Mashhad, Iran. Vet Res Forum. 2015;6: 301–304.

540. Tangtrongsup S, Scorza AV, Reif JS, Ballweber LR, Lappin MR, Salman MD. Seasonal distributions and other risk factors for *Giardia duodenalis* and *Cryptosporidium* spp. infections in dogs and cats in Chiang Mai, Thailand. Preventive Veterinary Medicine. 2020;174: 104820. doi:10.1016/j.prevetmed.2019.104820

541. Tauni MA, öSterlund A. Outbreak of Salmonella typhimurium in cats and humans associated with infection in wild birds. Journal of Small Animal Practice. 2000;41: 339–341. doi:10.1111/j.1748-5827.2000.tb03214.x

542. Tehrani-sharif M, Jahan S, Alavi SM, Khodami M. Seroprevalence of Toxoplasma gondii antibodies of stray cats in Garmsar, Iran. J Parasit Dis. 2015;39: 306–308. doi:10.1007/s12639-013-0349-7

543. Teixeira JV, Oliveira JLS de, Almeida DMPF de, Gonçalves L de S, Oliveira FLL de. Seroprevalence of feline toxoplasmosis in Teresina, Piauí, Brazil. Revista Brasileira de Higiene e Sanidade Animal. 2016;10: 549–555. doi:10.5935/rbhsa.v10i4.346

544. Tenter AM, Vietmeyer C, Johnson AM, Janitschke K, Rommel M, Lehmacher W. ELISAs based on recombinant antigens for sero-epidemiological studies on Toxoplasma gondii infections in cats. Parasitology. 1994;109: 29–36. doi:10.1017/S0031182000077738

545. Thépault A, Rose V, Queguiner M, Chemaly M, Rivoal K. Dogs and Cats: Reservoirs for Highly Diverse Campylobacter jejuni and a Potential Source of Human Exposure. Animals. 2020;10: 838. doi:10.3390/ani10050838

546. Thompson D. Successful treatment of Yersinia pseudotuberculosis hepatitis in a cat presenting with neurological abnormalities. Journal of Feline Medicine and Surgery Open Reports. 2019;5: 2055116919853644. doi:10.1177/2055116919853644

547. Tiao N, Darrington C, Molla B, Saville WJA, Tilahun G, Kwok OCH, et al. An investigation into the seroprevalence of Toxoplasma gondii, Bartonella spp., feline immunodeficiency virus (FIV), and feline leukaemia virus (FeLV) in cats in Addis Ababa, Ethiopia. Epidemiology & Infection. 2013;141: 1029–1033. doi:10.1017/S0950268812001707

548. Torkan S, Ghandehari-Alavijeh MR, Khamesipour F. Survey of the prevalence of Toxocara cati in stray cats in Isfahan city, Iran by PCR method. Trop Biomed. 2017;34: 550–555.

549. Tørnqvist-Johnsen C, Dickson S-A, Rolph K, Palermo V, Hodgkiss-Geere H, Gilmore P, et al. First report of Lyme borreliosis leading to cardiac bradydysrhythmia in two cats. Journal of Feline Medicine and Surgery Open Reports. 2020;6: 2055116919898292. doi:10.1177/2055116919898292

550. Torrejón E, Sanches GS, Moerbeck L, Santos L, André MR, Domingos A, et al. Molecular Survey of Bartonella Species in Stray Cats and Dogs, Humans, and Questing Ticks from Portugal. Pathogens. 2022;11: 749. doi:10.3390/pathogens11070749

551. Torrico KJ, Santos NJR dos, Abate HL, Martins FDC, Barros LD de, Seixas M, et al. Occurrence of gastrointestinal protozoans in cats from Londrina, Paraná, Brazil. Semina: Ciências Agrárias. 2020;41: 213–222. doi:10.5433/1679-0359.2020v41n1p213

552. Trataris AN, Rossouw J, Arntzen L, Karstaedt A, Frean J. Bartonella spp. in human and animal populations in Gauteng, South Africa, from 2007 to 2009. Onderstepoort J Vet Res. 2012;79: 452. doi:10.4102/ojvr.v79i2.452

553. Traversa D, Cesare AD, Milillo P, Iorio R, Otranto D. Infection by *Eucoleus aerophilus* in dogs and cats: Is another extra-intestinal parasitic nematode of pets emerging in Italy? Research in Veterinary Science. 2009;87: 270–272. doi:10.1016/j.rvsc.2009.02.006

554. Traversa D, Morelli S, Cassini R, Crisi PE, Russi I, Grillotti E, et al. Occurrence of canine and feline extra-intestinal nematodes in key endemic regions of Italy. Acta Tropica. 2019;193: 227–235. doi:10.1016/j.actatropica.2019.03.009

555. Truong QL, Seo TW, Yoon B-I, Kim H-C, Han JH, Hahn T-W. Prevalence of Swine Viral and Bacterial Pathogens in Rodents and Stray Cats Captured around Pig Farms in Korea. J Vet Med Sci. 2013;75: 1647–1650. doi:10.1292/jvms.12-0568

556. Tull A, Moks E, Saarma U. Endoparasite prevalence and infection risk factors among cats in an animal shelter in Estonia. Folia Parasitol (Praha). 2021;68: 2021.010. doi:10.14411/fp.2021.010

557. Turlewicz-Podbielska H, Ruszkowski JJ, Gogulski M, Pomorska-Mól M. Seroprevalence of Toxoplasma gondii in domestic cats, dogs and rabbits from Poland. Vet Res Commun. 2023;47: 1753–1758. doi:10.1007/s11259-022-10055-0

558. Tuska-Szalay B, Boldogh SA, Farkas R, Rompos L, Takács N, Beresnyák V, et al. Screening of Domestic Cats from North-Eastern Hungary for Hepatozoon felis and Cytauxzoon europaeus That Cause Infections in Local Wildcat Populations. Pathogens. 2023;12: 656. doi:10.3390/pathogens12050656

559. Ubirajara Filho CRC, Santos KKF, Lima T a. RF, Alves LC, Carvalho GA, Ramos R a. N. Gastrointestinal parasites in dogs and cats in line with the One Health’ approach. Arq Bras Med Vet Zootec. 2022;74: 43–50. doi:https://doi.org/10.1590/1678-4162-12355

560. Uhart MM, Rago MV, Marull CA, Ferreyra H del V, Pereira JA. Exposure to selected Pathogens in to selected pathogens in Geoffroy’s cats and domestic carnivores from central Argentina. J Wildl Dis. 2012;48: 899–909. doi:10.7589/2011-05-137

561. Umhang G, Forin-Wiart M-A, Hormaz V, Caillot C, Boucher J-M, Poulle M-L, et al. Echinococcus multilocularis detection in the intestines and feces of free-ranging domestic cats (Felis s. catus) and European wildcats (Felis s. silvestris) from northeastern France. Vet Parasitol. 2015;214: 75–79. doi:10.1016/j.vetpar.2015.06.006

562. Ursache AL, Györke A, Mircean V, Dumitrache MO, Codea AR, Cozma V. Toxocara cati and Other Parasitic Enteropathogens: More Commonly Found in Owned Cats with Gastrointestinal Signs Than in Clinically Healthy Ones. Pathogens. 2021;10: 198. doi:10.3390/pathogens10020198

563. Valenzuela-Moreno LF, Rico-Torres CP, Cedillo-Peláez C, Luna-Pastén H, Méndez-Cruz ST, Lara-Martínez G, et al. Mixed *Toxoplasma gondii* infection and new genotypes in feral cats of Quintana Roo, México. Acta Tropica. 2019;193: 199–205. doi:10.1016/j.actatropica.2019.03.006

564. Vanparijs O, Hermans L, van der Flaes L. Helminth and protozoan parasites in dogs and cats in Belgium. Veterinary Parasitology. 1991;38: 67–73. doi:10.1016/0304-4017(91)90010-S

565. VanWormer E, Conrad PA, Miller MA, Melli AC, Carpenter TE, Mazet JAK. Toxoplasma gondii, Source to Sea: Higher Contribution of Domestic Felids to Terrestrial Parasite Loading Despite Lower Infection Prevalence. EcoHealth. 2013;10: 277–289. doi:10.1007/s10393-013-0859-x

566. Verma SK, Minicucci L, Murphy D, Carstensen M, Humpal C, Wolf P, et al. Antibody Detection and Molecular Characterization of Toxoplasma gondii from Bobcats (Lynx rufus), Domestic Cats (Felis catus), and Wildlife from Minnesota, USA. Journal of Eukaryotic Microbiology. 2016;63: 567–571. doi:10.1111/jeu.12301

567. Veronesi F, Santoro A, Milardi GL, Diaferia M, Morganti G, Ranucci D, et al. Detection of Toxoplasma gondii in faeces of privately owned cats using two PCR assays targeting the B1 gene and the 529-bp repetitive element. Parasitol Res. 2017;116: 1063–1069. doi:10.1007/s00436-017-5388-z

568. Veyna-Salazar NP, Cantó-Alarcón GJ, Olvera-Ramírez AM, Ruiz-López FJ, Bernal-Reynaga R, Bárcenas-Reyes I, et al. Occurrence of Giardia duodenalis in Cats from Queretaro and the Risk to Public Health. Animals. 2023;13: 1098. doi:10.3390/ani13061098

569. Vincy P, Tresamol PV. Prevalence of gastro-intestinal and haemoparasitic infections among domestic cats of Kerala. J Parasit Dis. 2023;47: 562–565. doi:10.1007/s12639-023-01599-2

570. Vollaire MR, Radecki SV, Lappin MR. Seroprevalence of Toxoplasma gondii antibodies in clinically ill cats in the United States. American Journal of Veterinary Research. 2005;66: 874–877. doi:10.2460/ajvr.2005.66.874

571. Waap H, Gomes J, Nunes T. Parasite communities in stray cat populations from Lisbon, Portugal. J Helminthol. 2014;88: 389–395. doi:10.1017/S0022149X1300031X

572. Waap H, Cardoso R, Leitão A, Nunes T, Vilares A, Gargaté MJ, et al. *In vitro* isolation and seroprevalence of *Toxoplasma gondii* in stray cats and pigeons in Lisbon, Portugal. Veterinary Parasitology. 2012;187: 542–547. doi:10.1016/j.vetpar.2012.01.022

573. Wang Q, Jiang W, Chen Y-J, Liu C-Y, Shi J, Li X. Prevalence of Toxoplasma gondii antibodies, circulating antigens and DNA in stray cats in Shanghai, China. Parasites & Vectors. 2012;5: 190. doi:10.1186/1756-3305-5-190

574. Wang S, Zhou Y, Niu J, Xie Q, Xiao T, Chen Y, et al. Seroprevalence of Toxoplasma gondii infection in domestic cats in central China. Parasite. 2017;24: 10. doi:10.1051/parasite/2017010

575. Weis S, Rettinger A, Bergmann M, Llewellyn JR, Pantchev N, Straubinger RK, et al. Detection of Leptospira DNA in urine and presence of specific antibodies in outdoor cats in Germany. J Feline Med Surg. 2017;19: 470–476. doi:10.1177/1098612X16634389

576. Wieland B, Regula G, Danuser J, Wittwer M, Burnens AP, Wassenaar TM, et al. Campylobacter spp. in Dogs and Cats in Switzerland: Risk Factor Analysis and Molecular Characterization with AFLP. Journal of Veterinary Medicine, Series B. 2005;52: 183–189. doi:10.1111/j.1439-0450.2005.00843.x

577. Willi B, Boretti FS, Baumgartner C, Tasker S, Wenger B, Cattori V, et al. Prevalence, risk factor analysis, and follow-up of infections caused by three feline hemoplasma species in cats in Switzerland. J Clin Microbiol. 2006;44: 961–969. doi:10.1128/JCM.44.3.961-969.2006

578. Wright I, Stafford K, Coles G. The prevalence of intestinal nematodes in cats and dogs from Lancashire, north-west England. Journal of Small Animal Practice. 2016;57: 393–395. doi:10.1111/jsap.12478

579. Wu S, Meng J, Yu F, Zhou C, Yang B, Chen X, et al. Molecular epidemiological investigation of piroplasms carried by pet cats and dogs in an animal hospital in Guiyang, China. Front Microbiol. 2023;14. doi:10.3389/fmicb.2023.1266583

580. Wu S-M, Zhu X-Q, Zhou D-H, Fu B-Q, Chen J, Yang J-F, et al. Seroprevalence of Toxoplasma gondii infection in household and stray cats in Lanzhou, northwest China. Parasit Vectors. 2011;4: 214. doi:10.1186/1756-3305-4-214

581. Wyrosdick HM, Chapman A, Martinez J, Schaefer JJ. Parasite prevalence survey in shelter cats in Citrus County, Florida. Veterinary Parasitology: Regional Studies and Reports. 2017;10: 20–24. doi:10.1016/j.vprsr.2017.07.002

582. Xia N, Ji N, Li L, Huang Y, Yang C, Guo X, et al. Seroprevalence and risk factors of Toxoplasma gondii in urban cats from China. BMC Vet Res. 2022;18: 331. doi:10.1186/s12917-022-03427-w

583. Yager JA, Hutchison L, Barrett JW. Raccoonpox in a Canadian cat. Veterinary Dermatology. 2006;17: 443–448. doi:10.1111/j.1365-3164.2006.00553.x

584. Yamaguchi N, Macdonald DW, Passanisi WC, Harbour DA, Hopper CD. Parasite prevalence in free-ranging farm cats, Felis silvestris catus. Epidemiology & Infection. 1996;116: 217–223. doi:10.1017/S0950268800052468

585. Yang C, Liu S, Tao C, Yu J, Yang M, Guo L, et al. Serological and molecular survey of Toxoplasma gondii infection and associated risk factors in urban cats in Kunming, Southwest China. Front Vet Sci. 2024;11. doi:10.3389/fvets.2024.1393236

586. Yang R, Ying JLJ, Monis P, Ryan U. Molecular characterisation of *Cryptosporidium* and *Giardia* in cats (*Felis catus*) in Western Australia. Experimental Parasitology. 2015;155: 13–18. doi:10.1016/j.exppara.2015.05.001

587. Ybañez RHD, Busmeon CGR, Viernes ARG, Langbid JZ, Nuevarez JP, Ybañez AP, et al. Endemicity of Toxoplasma infection and its associated risk factors in Cebu, Philippines. PLOS ONE. 2019;14: e0217989. doi:10.1371/journal.pone.0217989

588. Yekkour F, Aubert D, Mercier A, Murat J-B, Khames M, Nguewa P, et al. First genetic characterization of *Toxoplasma gondii* in stray cats from Algeria. Veterinary Parasitology. 2017;239: 31–36. doi:10.1016/j.vetpar.2017.04.013

589. Yoshiuchi R, Matsubayashi M, Kimata I, Furuya M, Tani H, Sasai K. Survey and molecular characterization of *Cryptosporidium* and *Giardia* spp. in owned companion animal, dogs and cats, in Japan. Veterinary Parasitology. 2010;174: 313–316. doi:10.1016/j.vetpar.2010.09.004

590. Yun CS, Moon B-Y, Lee K, Kang SM, Ku B-K, Hwang M-H. The detection and phylogenetic characterization of Cryptosporidium, Cystoisospora, and Giardia duodenalis of cats in South Korea. Front Cell Infect Microbiol. 2023;13. doi:10.3389/fcimb.2023.1296118

591. Zaidi S, Korba AA, Bessas A, Bouzenad A, Hamnoune NK, Hezil D, et al. Serological study of leptospirosis in cats from Algeria. African Journal of Clinical and Experimental Microbiology. 2022;23: 416–425.

592. Zaidi S, Bouam A, Bessas A, Hezil D, Ghaoui H, Ait-Oudhia K, et al. Urinary shedding of pathogenic Leptospira in stray dogs and cats, Algiers: A prospective study. PLOS ONE. 2018;13: e0197068. doi:10.1371/journal.pone.0197068

593. Žákovská A, Schánilec P, Treml F, Dušková M, Agudelo RC. Seroprevalence of Antibodies against *Borrelia burgdorferi* s. l. and *Leptospira interrogans* s. l. in Cats in district of Brno and its environs, the Czech Republic. Ann Agric Environ Med. 2020;27: 356–360. doi:10.26444/aaem/122804

594. Zamora-Vélez A, Triviño J, Cuadrado-Ríos S, Lora-Suarez F, Enrique Gómez-Marín J. Detection and genotypes of Toxoplasma gondii DNA in feces of domestic cats in Colombia. Parasite. 2020;27: 25. doi:10.1051/parasite/2020023

595. Zanzani SA, Gazzonis AL, Scarpa P, Berrilli F, Manfredi MT. Intestinal Parasites of Owned Dogs and Cats from Metropolitan and Micropolitan Areas: Prevalence, Zoonotic Risks, and Pet Owner Awareness in Northern Italy. BioMed Research International. 2014;2014: e696508. doi:10.1155/2014/696508

596. Zarea AAK, Tempesta M, Fouad EA, Ndiana LA, Mahmoud MS, Mrenoshki D, et al. Prevalence of *Bartonella* spp., haemotropic *Mycoplasma* spp. and others vector-borne pathogens in private-owned dogs and cats, Egypt. Acta Tropica. 2023;240: 106857. doi:10.1016/j.actatropica.2023.106857

597. Zarea AAK, Bezerra-Santos MA, Nguyen V-L, Colella V, Dantas-Torres F, Halos L, et al. Occurrence and bacterial loads of Bartonella and haemotropic Mycoplasma species in privately owned cats and dogs and their fleas from East and Southeast Asia. Zoonoses and Public Health. 2022;69: 704–720. doi:10.1111/zph.12959

598. Zecca IB, Hodo CL, Slack S, Auckland L, Rodgers S, Killets KC, et al. Prevalence of *Trypanosoma cruzi* infection and associated histologic findings in domestic cats (*Felis catus*). Veterinary Parasitology. 2020;278: 109014. doi:10.1016/j.vetpar.2019.109014

599. Zhang H, Zhou DH, Zhou P, Lun ZR, Chen XG, Lin RQ, et al. Seroprevalence of Toxoplasma gondii Infection in Stray and Household Cats in Guangzhou, China. Zoonoses and Public Health. 2009;56: 502–505. doi:10.1111/j.1863-2378.2008.01209.x

600. Zhang X-L, Li X-W, Li W-J, Huang H-L, Huang S-J, Shao J-W. Molecular evidence of Babesia in pet cats in mainland China. BMC Veterinary Research. 2019;15: 476. doi:10.1186/s12917-019-2214-0

601. Zheng G, Hu W, Liu Y, Luo Q, Tan L, Li G. Occurrence and Molecular Identification of <italic>Giardia duodenalis</italic> from Stray Cats in Guangzhou, Southern China. Korean J Parasito. 2015;53: 119–124. doi:10.3347/kjp.2015.53.1.119

602. Zhu S, Camp L, Patel A, VanWormer E, Shapiro K. High prevalence and diversity of Toxoplasma gondii DNA in feral cat feces from coastal California. PLOS Neglected Tropical Diseases. 2023;17: e0011829. doi:10.1371/journal.pntd.0011829

603. Zibaei M, Sadjjadi SM, Sarkari B. Prevalence of Toxocara cati and other intestinal helminths in stray cats in Shiraz, Iran. Trop Biomed. 2007;24: 39–43.

604. Zottler E-M, Bieri M, Basso W, Schnyder M. Intestinal parasites and lungworms in stray, shelter and privately owned cats of Switzerland. Parasitology International. 2019;69: 75–81. doi:10.1016/j.parint.2018.12.005
