# Supplementary material for: Outdoor roaming of owned cats elevates risk of zoonotic pathogen exposure: A global synthesis
Source: PLoS Pathog. 2026 Apr 20;22(4):e1014160. doi: 10.1371/journal.ppat.1014160 (PMC13128103; doi:10.1371/journal.ppat.1014160)
Supplement: S4 Table — (DOCX) [file ppat.1014160.s004.docx]

**S4 Table.** Studies identified during the literature search but excluded from data extraction and analysis, with reasons for exclusion.

| **Reference** | **Exclusion** |
| --- | --- |
| Afshar M, Zahabiun F, Heydarian P, Saadati H, Mohtasebi S, Khodamoradi F, et al. A Systematic Review and Meta-analysis of Toxocariasis in Iran: Is it Time to Take it Seriously? ACTA PARASITOLOGICA. 2020;65: 569–584. doi:10.2478/s11686-020-00195-1 | Sampling design |
| Agu N, Okoye I, Nwosu C, Onyema I, Iheagwam C, Anunobi T. Prevalence of Ectoparasites Infestation among Companion Animals in Nsukka Cultural Zone. ANNALS OF MEDICAL AND HEALTH SCIENCES RESEARCH. 2020;10: 1050–1057. | Sampling design |
| Aksulu A, Bilgiç H, Karagenç T, Bakirci S. Seroprevalence and molecular detection of Leishmania spp. in cats of West Aegean Region, Turkey. VETERINARY PARASITOLOGY- REGIONAL STUDIES AND REPORTS. 2021;24. doi:10.1016/j.vprsr.2021.100573 | Shelter only |
| Al-Adhami B, Gajadhar A. A new multi-host species indirect ELISA using protein A/G conjugate for detection of anti-Toxoplasma gondii IgG antibodies with comparison to ELISA-IgG, agglutination assay and Western blot. VETERINARY PARASITOLOGY. 2014;200: 66–73. doi:10.1016/j.vetpar.2013.11.004 | Sampling design |
| Alvarado-Hidalgo I, Campos-Camacho J, Arguedas-Morales Y, Romero-Vega L, Alfaro-Alarcón A, Anchia-Ureña G, et al. Molecular, morphological and histopathological evidence of Spirometra mansoni in wild and domestic animals from Costa Rica. VETERINARY PARASITOLOGY- REGIONAL STUDIES AND REPORTS. 2024;51. doi:10.1016/j.vprsr.2024.101030 | Sampling design |
| Arai H, Fukuda Y, Hara T, Funakoshi Y, Kaneko S, Yoshida T, et al. Prevalence of Cryptosporidium infection among domestic cats in the Tokyo Metropolitan District, Japan. Jpn J Med Sci Biol. 1990;43: 7–14. doi:10.7883/yoken1952.43.7 | Sampling design |
| Arruda IF, Ramos RCF, Barbosa ADS, Villar-Echarte G, Augusto AM, Troccoli F, et al. Occurrence of Toxoplasma gondii and other gastrointestinal parasites in free-roaming cats from the Rio de Janeiro zoo. Braz J Vet Med. 2023;45: e006023. doi:10.29374/2527-2179.bjvm006023 | Wrong setting |
| Aslan Çelík B, Çelik Ö, Ayan A, Akyildiz G, Orunç Kilinç Ö, Oktay Ayan Ö, et al. Preliminary investigation of the prevalence and genotype distribution of Cryptosporidium spp., and Giardia duodenalis in cats in Siirt, Turkey. ACTA VETERINARIA-BEOGRAD. 2023;73: 317–324. doi:10.2478/acve-2023-0024 | Cat lifestyles joined |
| Ayan A, Çelik B, Çelik O, Yilmaz A, Kilinç Ö, Ayan Ö. Molecular detection and subtype distribution of Blastocystis sp. from shelter dogs and cats in Van, Turkey: First report of ST10 in cats and ST1, ST10 and ST30 in dogs. POLISH JOURNAL OF VETERINARY SCIENCES. 2024;27: 389–395. doi:10.24425/pjvs.2024.151732 | Shelter only |
| Ballweber LR, Panuska C, Huston CL, Vasilopulos R, Pharr GT, Mackin A. Prevalence of and risk factors associated with shedding of Cryptosporidium felis in domestic cats of Mississippi and Alabama. Vet Parasitol. 2009;160: 306–10. doi:10.1016/j.vetpar.2008.11.018 | Wrong setting |
| Bastien M, Vaniscotte A, Combes B, Umhang G, Germain E, Gouley V, et al. High density of fox and cat faeces in kitchen gardens and resulting rodent exposure to Echinococcus multilocularis and Toxoplasma gondii. FOLIA PARASITOLOGICA. 2018;65. doi:10.14411/fp.2018.002 | Environmental survey |
| Beelitz P, Göbel E, Gothe R. [Fauna and incidence of endoparasites in kittens and their mothers from different husbandry situations in south Germany]. Tierarztl Prax. 1992;20: 297–300. | Language |
| Bermúdez S, Miranda R, Zaldívar Y, González P, Berguido G, Trejos D, et al. [Detection of Rickettsia in ectoparasites of wild and domestic mammals from the Cerro Chucanti private reserve and from neighboring towns, Panamá, 2007-2010]. Biomedica. 2012;32: 189–95. doi:10.1590/S0120-41572012000300006 | Sampling design |
| Blagburn BL, Schenker R, Gagne F, Drake J. Prevalence of intestinal parasites in companion animals in Ontario and Quebec, Canada, during the winter months. Vet Ther. 2008;9: 169–75. | Unavailable |
| Bonilla-Aldana J, Espinosa-Nuñez A, Bonilla-Aldana D, Rodriguez-Morales A. Toxocara cati Infection in Cats (Felis catus): A Systematic Review and Meta-Analysis. ANIMALS. 2024;14. doi:10.3390/ani14071022 | Sampling design |
| Bouzid M, Halai K, Jeffreys D, Hunter PR. The prevalence of Giardia infection in dogs and cats, a systematic review and meta-analysis of prevalence studies from stool samples. Vet Parasitol. 2015;207: 181–202. doi:10.1016/j.vetpar.2014.12.011 | Sampling design |
| Calvani N, Wright M, White J, Stepkovitch B, Francis E, Rivory P, et al. What the fox? Cryptic Eucoleus [Capillaria] sp. in the respiratory tract of a cat from Australia. CURRENT RESEARCH IN PARASITOLOGY & VECTOR-BORNE DISEASES. 2021;1. doi:10.1016/j.crpvbd.2021.100028 | Sampling design |
| Carlin EP, Bowman DD, Scarlett JM, Garrett J, Lorentzen L. Prevalence of Giardia in symptomatic dogs and cats throughout the United States as determined by the IDEXX SNAP Giardia test. Vet Ther. 2006;7: 199–206. | Sampling design |
| Chai JY, Bahk YY, Sohn WM. Trematodes recovered in the small intestine of stray cats in the Republic of Korea. Korean J Parasitol. 2013;51: 99–106. doi:10.3347/kjp.2013.51.1.99 | Sampling design |
| Chalkowski K, Wilson A, Lepczyk C, Zohdy S. Who let the cats out? A global meta-analysis on risk of parasitic infection in indoor versus outdoor domestic cats (Felis catus). BIOLOGY LETTERS. 2019;15. doi:10.1098/rsbl.2018.0840 | Sampling design |
| Chodun-Wroblewska W, Nieradko-Iwanicka B, Iwanicki J. Testing sand and soil from selected playgrounds in Lublin for eggs of nematodes of the genus: Ascaris, Toxocara, Trichuris. CURRENT ISSUES IN PHARMACY AND MEDICAL SCIENCES. 2022;35: 133–136. doi:10.2478/cipms-2022-0025 | Environmental survey |
| Christensen LS, Jacobsen K, Maersk-Møller E. [Rabies in a cat in Greenland]. Ugeskr Laeger. 2008;170: 2584. | Sampling design |
| Christie E, Dubey JP, Pappas PW. Prevalence of Sarcocystis infection and other intestinal parasitisms in cats from a humane shelter in Ohio. J Am Vet Med Assoc. 1976;168: 421–2. | Before 1980 |
| Chuong LS, Suresh K, Mak JW, Init I, Kathijah O. Prevalence of Blastocystis in animals from domesticated surroundings. Southeast Asian J Trop Med Public Health. 1996;27: 850–2. | Unavailable |
| Coati N, Hellmann K, Mencke N, Epe C. Recent investigation on the prevalence of gastrointestinal nematodes in cats from France and Germany. Parasitol Res. 2003;90 Suppl 3: S146-7. doi:10.1007/s00436-003-0921-7 | Sampling design |
| Cohn LA, Middleton JR. A veterinary perspective on methicillin-resistant staphylococci. J Vet Emerg Crit Care (San Antonio). 2010;20: 31–45. doi:10.1111/j.1476-4431.2009.00497.x | Sampling design |
| Correa V, Briceño J, Zúñiga J, Aranda JC, Valdés J, Contreras MC, et al. [Trypanosoma cruzi infection in domestic animals in rural sections of the IV Region, Chile]. Bol Chil Parasitol. 1982;37: 27–8. | Unavailable |
| Costy F. [Rabies, a public health problem]. Arch Belg. 1989;47: 123–7. | Sampling design |
| Dąbrowska J, Karamon J, Kochanowski M, Jędryczko R, Cencek T. Tritrichomonas foetus infection in cat - first detection in Poland. Acta Parasitol. 2015;60: 605–8. doi:10.1515/ap-2015-0084 | Sampling design |
| de Oliveira A, Sudré A, do Bomfim T, Santos H. Molecular characterization of Cryptosporidium spp. in dogs and cats in the city of Rio de Janeiro, Brazil, reveals potentially zoonotic species and genotype. PLOS ONE. 2021;16. doi:10.1371/journal.pone.0255087 | Cat lifestyles joined |
| de Quadros R, Trevisani N, de Moura A, Ramos C. Parasitic helminthofauna in Wandering Cats of Lages, Santa Catarina, Brazil. BRAZILIAN JOURNAL OF HYGIENE AND ANIMAL SANITY. 2021;15. | Unavailable |
| De Santis-Kerr AC, Raghavan M, Glickman NW, Caldanaro RJ, Moore GE, Lewis HB, et al. Prevalence and risk factors for Giardia and coccidia species of pet cats in 2003-2004. J Feline Med Surg. 2006;8: 292–301. doi:10.1016/j.jfms.2006.02.005 | Sampling design |
| de Souza FB, Nakiri IM, Lourenço NO, da Silva GG, Paschoalini DR, Guimarães-Okamoto PTC, et al. Prevalence of Intestinal Endoparasites With Zoonotic Potential in Domestic Cats From Botucatu, SP, Brazil. Top Companion Anim Med. 2017;32: 114–117. doi:10.1053/j.tcam.2017.10.004 | Cat lifestyles joined |
| Deiró A, do Prado D, Sousa I, Rocha D, Bezerra R, Gaiotto F, et al. Presence of atypical genotypes of Toxoplasma gondii isolated from cats in the state of Bahia, Northeast of Brazil. PLOS ONE. 2021;16. doi:10.1371/journal.pone.0253630 | Sampling design |
| Deplazes P, Alther P, Tanner I, Thompson RC, Eckert J. Echinococcus multilocularis coproantigen detection by enzyme-linked immunosorbent assay in fox, dog, and cat populations. J Parasitol. 1999;85: 115–21. | Sampling design |
| Deplazes P, van Knapen F, Schweiger A, Overgaauw P. Role of pet dogs and cats in the transmission of helminthic zoonoses in Europe, with a focus on echinococcosis and toxocarosis. VETERINARY PARASITOLOGY. 2011;182: 41–53. doi:10.1016/j.vetpar.2011.07.014 | Sampling design |
| do Prado CM, Razzolini E, Santacruz G, Ojeda L, Geraldo MR, Segovia N, et al. First Cases of Feline Sporotrichosis Caused by Sporothrix brasiliensis in Paraguay. Journal of Fungi. 2023;9: 972. doi:10.3390/jof9100972 | Sampling design |
| dos Santos N, de Pinho F, Hlavac N, Nunes T, Almeida N, Solcà M, et al. Feline Leishmaniasis Caused by Leishmania infantum: Parasite Sequencing, Seropositivity, and Clinical Characterization in an Endemic Area From Brazil. FRONTIERS IN VETERINARY SCIENCE. 2021;8. doi:10.3389/fvets.2021.734916 | Cat lifestyles joined |
| Dumonteil E, Desale H, Tu W, Duhon B, Wolfson W, Balsamo G, et al. Shelter cats host infections with multiple Trypanosoma cruzi discrete typing units in southern Louisiana. VETERINARY RESEARCH. 2021;52. doi:10.1186/s13567-021-00923-z | Shelter only |
| Dymon M, Ramisz A, Sokołowska B, Zemburowa K, Umiński J, Krupa K. [Toxoplasma gondii infection among wild and domestic animals]. Wiad Parazytol. 1988;34: 690–704. | Unavailable |
| Egger M, Nguyen XM, Schaad UB, Krech T. Intestinal cryptosporidiosis acquired from a cat. Infection. 1990;18: 177–8. doi:10.1007/BF01642109 | Sampling design |
| Epe C, Coati N, Schnieder T. [Results of parasitological examinations of faecal samples from horses, ruminants, pigs, dogs, cats, hedgehogs and rabbits between 1998 and 2002]. Dtsch Tierarztl Wochenschr. 2004;111: 243–7. | Language |
| Epe C, Ising-Volmer S, Stoye M. [Parasitological fecal studies of equids, dogs, cats and hedgehogs during the years 1984-1991]. Dtsch Tierarztl Wochenschr. 1993;100: 426–8. | Language |
| Epe C, Rehkter G, Schnieder T, Lorentzen L, Kreienbrock L. Giardia in symptomatic dogs and cats in Europe--results of a European study. Vet Parasitol. 2010;173: 32–8. doi:10.1016/j.vetpar.2010.06.015 | Sampling design |
| Fang F, Li J, Huang T, Guillot J, Huang W. Zoonotic helminths parasites in the digestive tract of feral dogs and cats in Guangxi, China. BMC Vet Res. 2015;11: 211. doi:10.1186/s12917-015-0521-7 | Sampling design |
| Fayer R, Santín M, Trout JM, Dubey JP. Detection of Cryptosporidium felis and Giardia duodenalis Assemblage F in a cat colony. Vet Parasitol. 2006;140: 44–53. doi:10.1016/j.vetpar.2006.03.005 | Sampling design |
| Fernandes AB, Baêta Bde A, Filho WF, Massad FV, Rebouças FA, De Carvalho JB, et al. [Relationship between companion animals and intestinal parasites in children at municipality of Seropédica, RJ]. Rev Bras Parasitol Vet. 2008;17 Suppl 1: 296–300. | Language |
| Ferraroni JJ, Marzochi MC. [Prevalence of Toxoplasma gondii infection in domestic and wild animals, and human groups of the Amazonas region]. Mem Inst Oswaldo Cruz. 1980;75: 99–109. doi:10.1590/s0074-02761980000100010 | Cat lifestyles joined |
| Ferreira F, Dias R, Martins T, Constantino C, Pasquali A, Vidotto O, et al. Frequency of gastrointestinal parasites in dogs and cats of Londrina, PR, focusing on public health. SEMINA-CIENCIAS AGRARIAS. 2013;34: 3851–3858. doi:10.5433/1679-0359.2013v34n6Supl2p3851 | Language |
| Ferrés M, Abarca K, Godoy P, García P, Palavecino E, Méndez G, et al. [Presence of Bartonella henselae in cats: natural reservoir quantification and human exposition risk of this zoonoses in Chile]. Rev Med Chil. 2005;133: 1465–71. doi:10.4067/s0034-98872005001200008 | Cat lifestyles joined |
| Franjola R, Matzner N. [Prevalence of intestinal protozoa in domestic cats in the city of Valdivia, Chile]. Zentralbl Veterinarmed B. 1982;29: 397–400. | Language |
| Galván-Díaz A, Magnet A, Fenoy S, Henriques-Gil N, Haro M, Gordo F, et al. Microsporidia Detection and Genotyping Study of Human Pathogenic E. bieneusi in Animals from Spain. PLOS ONE. 2014;9. doi:10.1371/journal.pone.0092289 | Cat lifestyles joined |
| Gawor J, Marczynska M. Threat of zoonotic geohelminths infection in humans in the urban and rural environment in Poland: the risk of toxocariasis. MEDYCYNA WETERYNARYJNA-VETERINARY MEDICINE-SCIENCE AND PRACTICE. 2015;71: 543–547. | Sampling design |
| Gebremedhin E, Tadesse G. A meta-analysis of the prevalence of Toxoplasma gondii in animals and humans in Ethiopia. PARASITES & VECTORS. 2015;8. doi:10.1186/s13071-015-0901-7 | Sampling design |
| Gibbons LM, Jacobs DE, Sani RA. Toxocara malaysiensis n. sp. (Nematoda: Ascaridoidea) from the domestic cat (Felis catus Linnaeus, 1758). J Parasitol. 2001;87: 660–5. doi:10.1645/0022-3395(2001)087%5B0660:TMNSNA%5D2.0.CO;2 | Sampling design |
| Gil H, Cano L, de Lucio A, Bailo B, de Mingo M, Cardona G, et al. Detection and molecular diversity of Giardia duodenalis and Cryptosporidium spp. in sheltered dogs and cats in Northern Spain. INFECTION GENETICS AND EVOLUTION. 2017;50: 62–69. doi:10.1016/j.meegid.2017.02.013 | Shelter only |
| Goodfellow M, Shaw S, Morgan E. Imported disease of dogs and cats exotic to Ireland:: Echinococcus multilocularis. IRISH VETERINARY JOURNAL. 2006;59: 214–216. doi:10.1186/2046-0481-59-4-214 | Sampling design |
| Gookin JL, Breitschwerdt EB, Levy MG, Gager RB, Benrud JG. Diarrhea associated with trichomonosis in cats. J Am Vet Med Assoc. 1999;215: 1450–4. | Sampling design |
| Gookin JL, Stebbins ME, Hunt E, Burlone K, Fulton M, Hochel R, et al. Prevalence of and risk factors for feline Tritrichomonas foetus and giardia infection. J Clin Microbiol. 2004;42: 2707–10. doi:10.1128/JCM.42.6.2707-2710.2004 | Sole pathogen not zoonotic |
| Goz Y, Yuksek N, Altug N, Ceylan E, Deger S. Prevalence of Cryptosporidium infection in Van Cats. INDIAN VETERINARY JOURNAL. 2005;82: 995–996. | Sampling design |
| Grobbelaar AA, Blumberg LH, Dermaux-Msimang V, Le Roux CA, Moolla N, Paweska JT, et al. Human rabies associated with domestic cat exposures in South Africa, 1983-2018. J S Afr Vet Assoc. 2020;91: e1–e4. doi:10.4102/jsava.v91i0.2036 | Sampling design |
| Gürler A, Bölükbas C, Pekmezci G, Umur S, Açici M. Nematode and cestode eggs scattered with cats-dogs feces and significance of public health in Samsun, Turkey. ANKARA UNIVERSITESI VETERINER FAKULTESI DERGISI. 2015;62: 23–26. doi:10.1501/Vetfak_0000002653 | Sampling design |
| Guterbock WM, Levine ND. Coccidia and intestinal nematodes of East Central Illinois cats. J Am Vet Med Assoc. 1977;170: 1411–3. | Before 1980 |
| Györke A, Balea A, Borsan S, Su C, Jiang T, Magdas C, et al. Toxoplasma gondii genotypes and frequency in domestic cats from Romania. BMC VETERINARY RESEARCH. 2024;20. doi:10.1186/s12917-024-04210-9 | Cat lifestyles joined |
| Hajipour N, Tavassoli M. Prevalence and associated risk factors of Linguatula serrata infection in definitive and intermediate hosts in Iran and other countries: A systematic review. VETERINARY PARASITOLOGY- REGIONAL STUDIES AND REPORTS. 2019;16. doi:10.1016/j.vprsr.2019.100288 | Sampling design |
| Hatam G, Adnani S, Asgari Q, Fallah E, Motazedian M, Sadjjadi S, et al. First Report of Natural Infection in Cats with Leishmania infantum in Iran. VECTOR-BORNE AND ZOONOTIC DISEASES. 2010;10: 313–316. doi:10.1089/vbz.2009.0023 | Sampling design |
| Hinney B, Ederer C, Stengl C, Wilding K, Štrkolcová G, Harl J, et al. Enteric protozoa of cats and their zoonotic potential-a field study from Austria. Parasitol Res. 2015;114: 2003–6. doi:10.1007/s00436-015-4408-0 | Cat lifestyles joined |
| Hinney B, Joachim A. Intestinal parasites of dogs and cats. KLEINTIERPRAXIS. 2013;58: 256-+. doi:10.2377/0023-2076-58-256 | Unavailable |
| Hoopes JH, Polley L, Wagner B, Jenkins EJ. A retrospective investigation of feline gastrointestinal parasites in western Canada. Can Vet J. 2013;54: 359–62. | Cat lifestyles joined |
| Horiuchi S, Paller V, Uga S. Soil contamination by parasite eggs in rural village in the Philippines. TROPICAL BIOMEDICINE. 2013;30: 495–503. | Environmental survey |
| Iastreb VB, Gorokhov VV, Shestakov AM. [To the detection of the trematode mesocercariae Alaria alata in the blood of domestic dogs and cats]. Med Parazitol (Mosk). 2005; 48–51. | Sampling design |
| Iatta R, Furlanello T, Colella V, Tarallo V, Latrofa M, Brianti E, et al. A nationwide survey of Leishmania infantum infection in cats and associated risk factors in Italy. PLOS NEGLECTED TROPICAL DISEASES. 2019;13. doi:10.1371/journal.pntd.0007594 | Cat lifestyles joined |
| Ibba F, Lepri E, Veronesi F, Di Cesare A, Paltrinieri S. Gastric cylicospirurosis in a domestic cat from Italy. J Feline Med Surg. 2014;16: 522–6. doi:10.1177/1098612X13505577 | Sampling design |
| Iijima Y, Itoh N, Ito Y, Kimura Y. Multilocus genotyping of Giardia duodenalis isolates from household cats and pet shop kittens. Vet Parasitol. 2018;259: 44–48. doi:10.1016/j.vetpar.2018.06.022 | Sampling design |
| Ito Y, Itoh N, Kimura Y, Kanai K. Prevalence of intestinal parasites in breeding cattery cats in Japan. J Feline Med Surg. 2016;18: 834–7. doi:10.1177/1098612X15597023 | Shelter only |
| Itoh N, Ito Y, Kato A, Kanai K, Chikazawa S, Hori Y, et al. Prevalence of intestinal parasites in pet shop kittens in Japan. J Feline Med Surg. 2013;15: 908–10. doi:10.1177/1098612X13487362 | Shelter only |
| Itoh N. [Survey on Toxocara cati in domestic cats]. Kansenshogaku Zasshi. 2000;74: 824–7. doi:10.11150/kansenshogakuzasshi1970.74.824 | Language |
| Janczak D, Górecki P, Maj A. Spirometra erinaceieuropaei infection in a cat imported into Poland from South Korea. MEDYCYNA WETERYNARYJNA-VETERINARY MEDICINE-SCIENCE AND PRACTICE. 2023;79: 648–651. doi:10.21521/mw.6825 | Sampling design |
| Jenkins E. Toxocara spp. in dogs and cats in Canada. TOXOCARA AND TOXOCARIASIS. 2020. pp. 641–653. doi:10.1016/bs.apar.2020.01.026 | Sampling design |
| Jitsamai W, Kamkong P, Traub R, Taweethavonsawat P. New insight into genetic diversity of zoonotic-potential Ancylostoma ceylanicum in stray cats living in Bangkok, Thailand, based on deep amplicon sequencing. ZOONOSES AND PUBLIC HEALTH. 2024;71: 236–247. doi:10.1111/zph.13102 | Sampling design |
| Jittapalapong S, Rtjngphisutthipongse O, Maruyama S, Schaefer J, Stich R. Detection of Hepatozoon canis in stray dogs and cats in Bangkok, Thailand. IMPACT OF EMERGING ZOONOTIC DISEASES ON ANIMAL HEALTH. 2006. pp. 479–488. doi:10.1196/annals.1373.071 | Sole pathogen not zoonotic |
| Joachim A, Auersperg V, Drüe J, Wiedermann S, Hinney B, Spergser J. Parasites and zoonotic bacteria in the feces of cats and dogs from animal shelters in Carinthia, Austria. Res Vet Sci. 2023;164: 105022. doi:10.1016/j.rvsc.2023.105022 | Shelter only |
| Jost R, Müller N, Marreros N, Moré G, Antoine L, Basso W, et al. What is the role of Swiss domestic cats in environmental contamination with Echinococcus multilocularis eggs? PARASITES & VECTORS. 2023;16. doi:10.1186/s13071-023-05983-y | Cat lifestyles joined |
| Karshima S, Karshima M, Karaye G, Oziegbe S. Toxoplasma gondii infections in birds, companion, food and recreational animals in Nigeria: A systematic review and meta-analysis. VETERINARY PARASITOLOGY- REGIONAL STUDIES AND REPORTS. 2020;21. doi:10.1016/j.vprsr.2020.100418 | Sampling design |
| Kellerová P, Tachezy J. Zoonotic Trichomonas tenax and a new trichomonad species, Trichomonas brixi n. sp., from the oral cavities of dogs and cats. INTERNATIONAL JOURNAL FOR PARASITOLOGY. 2017;47: 247–255. doi:10.1016/j.ijpara.2016.12.006 | Commensal organism |
| Khademvatan S, Rahim F, Tavalla M, Abdizadeh R, Hashemitabar M. PCR-based molecular characterization of Toxocara spp. using feces of stray cats: a study from Southwest Iran. PLoS One. 2013;8: e65293. doi:10.1371/journal.pone.0065293 | Environmental survey |
| Kim JH, Lee K, Sohn WM, Kim HY, Lee YR, Choi EJ, et al. Necrotizing Enteritis Caused by Pharyngostomum cordatum Infection in a Stray Cat. Korean J Parasitol. 2019;57: 17–20. doi:10.3347/kjp.2019.57.1.17 | Sampling design |
| Kirkpatrick CE, Laczak JP. Giardiasis in a cattery. J Am Vet Med Assoc. 1985;187: 161–2. | Shelter only |
| Kirkpatrick CE. Epizootiology of endoparasitic infections in pet dogs and cats presented to a veterinary teaching hospital. Vet Parasitol. 1988;30: 113–24. doi:10.1016/0304-4017(88)90158-6 | Pathogens compiled |
| Knapp J, Giraudoux P, Combes B, Umhang G, Boué F, Said-Ali Z, et al. Rural and urban distribution of wild and domestic carnivore stools in the context of Echinococcus multilocularis environmental exposure. INTERNATIONAL JOURNAL FOR PARASITOLOGY. 2018;48: 937–946. doi:10.1016/j.ijpara.2018.05.007 | Environmental survey |
| König G, Müller HE. Blastocystis hominis in animals: incidence of four serogroups. Zentralbl Bakteriol. 1997;286: 435–40. doi:10.1016/s0934-8840(97)80105-3 | Sampling design |
| Koompapong K, Mori H, Thammasonthijarern N, Prasertbun R, Pintong AR, Popruk S, et al. Molecular identification of Cryptosporidium spp. in seagulls, pigeons, dogs, and cats in Thailand. Parasite. 2014;21: 52. doi:10.1051/parasite/2014053 | Sampling design |
| Korkmaz UF, Gökpınar S, Yıldız K. Prevalence of Intestinal Parasites in Cats and Their Importance in Terms of Public Health. Turkiye Parazitol Derg. 2016;40: 194–198. doi:10.5152/tpd.2016.4841 | Pathogens compiled |
| Krauss H. [The importance of Rickettsiae and Chlamydia in small domestic animals as pathogens of zoonoses]. Berl Munch Tierarztl Wochenschr. 1982;95: 480–3. | Sampling design |
| Kuehner KA, Marks SL, Kass PH, Sauter-Louis C, Grahn RA, Barutzki D, et al. Tritrichomonas foetus infection in purebred cats in Germany: prevalence of clinical signs and the role of co-infection with other enteroparasites. J Feline Med Surg. 2011;13: 251–8. doi:10.1016/j.jfms.2010.12.002 | Sole pathogen not zoonotic |
| Latrofa M, Annoscia G, Colella V, Cavalera M, Maia C, Martin C, et al. A real-time PCR tool for the surveillance of zoonotic Onchocerca lupi in dogs, cats and potential vectors. PLOS NEGLECTED TROPICAL DISEASES. 2018;12. doi:10.1371/journal.pntd.0006402 | Sampling design |
| Le T, Anh N, Nguyen K, Nguyen N, Thuy D, Gasser R. Toxocara malaysiensis infection in domestic cats in Vietnam - An emerging zoonotic issue? INFECTION GENETICS AND EVOLUTION. 2016;37: 94–98. doi:10.1016/j.meegid.2015.11.009 | Sampling design |
| Lecová L, Hammerbauerová I, Tůmová P, Nohýnková E. Companion animals as a potential source of Giardia intestinalis infection in humans in the Czech Republic - A pilot study. Vet Parasitol Reg Stud Reports. 2020;21: 100431. doi:10.1016/j.vprsr.2020.100431 | Sampling design |
| Lester SJ, Kowalewich NJ, Bartlett KH, Krockenberger MB, Fairfax TM, Malik R. Clinicopathologic features of an unusual outbreak of cryptococcosis in dogs, cats, ferrets, and a bird: 38 cases (January to July 2003). J Am Vet Med Assoc. 2004;225: 1716–22. doi:10.2460/javma.2004.225.1716 | Sampling design |
| Leutenegger CM, Lozoya CE, Tereski J, Andrews J, Mitchell KD, Meeks C, et al. Comparative study of a broad qPCR panel and centrifugal flotation for detection of gastrointestinal parasites in fecal samples from dogs and cats in the United States. Parasit Vectors. 2023;16: 288. doi:10.1186/s13071-023-05904-z | Sampling design |
| Lightner L, Christensen BM, Beran GW. Epidemiologic findings on canine and feline intestinal nematode infections from records of the Iowa state University Veterinary Clinic. J Am Vet Med Assoc. 1978;172: 564–7. | Before 1980 |
| Lim S, Park SI, Ahn KS, Oh DS, Ryu JS, Shin SS. First report of feline intestinal trichomoniasis caused by Tritrichomonas foetus in Korea. Korean J Parasitol. 2010;48: 247–51. doi:10.3347/kjp.2010.48.3.247 | Sampling design |
| Lima JAS, Rezende HHA, Rocha TMDD, Castro AM. Analysis of the accuracy of different laboratory methods for the diagnosis of intestinal parasites from stray and domiciled cats (Felis catus domesticus) in Goiânia, Goiás, Brazil. Rev Bras Parasitol Vet. 2018;27: 95–98. doi:10.1590/S1984-29612018004 | Sampling design |
| Lima N, Raimundo D, de Souza V, Aguiar J. Occurrence of gastrointestinal parasites in dogs and cats domiciliated in Santos, SP, Brazil. REVISTA BRASILEIRA DE PARASITOLOGIA VETERINARIA. 2021;30. doi:10.1590/S1984-29612021080 | Cat lifestyles joined |
| Liu Y, Zheng G, Alsarakibi M, Zhang X, Hu W, Lu P, et al. Molecular identification of Ancylostoma caninum isolated from cats in southern China based on complete ITS sequence. Biomed Res Int. 2013;2013: 868050. doi:10.1155/2013/868050 | Sampling design |
| Loftin CM, Donnett UB, Schneider LG, Varela-Stokes AS. Prevalence of endoparasites in northern Mississippi shelter cats. Vet Parasitol Reg Stud Reports. 2019;18: 100322. doi:10.1016/j.vprsr.2019.100322 | Shelter only |
| López-Arias Á, Villar D, López-Osorio S, Calle-Vélez D, Chaparro-Gutiérrez JJ. Giardia is the most prevalent parasitic infection in dogs and cats with diarrhea in the city of Medellín, Colombia. Vet Parasitol Reg Stud Reports. 2019;18: 100335. doi:10.1016/j.vprsr.2019.100335 | Sampling design |
| Lucio-Forster A, Bowman DD. Prevalence of fecal-borne parasites detected by centrifugal flotation in feline samples from two shelters in upstate New York. J Feline Med Surg. 2011;13: 300–3. doi:10.1016/j.jfms.2010.12.013 | Shelter only |
| Lucio-Forster A, Griffiths JK, Cama VA, Xiao L, Bowman DD. Minimal zoonotic risk of cryptosporidiosis from pet dogs and cats. Trends Parasitol. 2010;26: 174–9. doi:10.1016/j.pt.2010.01.004 | Sampling design |
| Mahittikorn A, Udonsom R, Koompapong K, Chiabchalard R, Sutthikornchai C, Sreepian P, et al. Molecular identification of Pentatrichomonas hominis in animals in central and western Thailand. BMC VETERINARY RESEARCH. 2021;17. doi:10.1186/s12917-021-02904-y | Sole pathogen not zoonotic |
| Mallawarachchi C, Chandrasena N, Wickramasinghe S, Premaratna R, Gunawardane N, Mallawarachchi N, et al. A preliminary survey of filarial parasites in dogs and cats in Sri Lanka. PLOS ONE. 2018;13. doi:10.1371/journal.pone.0206633 | Sampling design |
| Marks SL, Hanson TE, Melli AC. Comparison of direct immunofluorescence, modified acid-fast staining, and enzyme immunoassay techniques for detection of Cryptosporidium spp in naturally exposed kittens. J Am Vet Med Assoc. 2004;225: 1549–53. doi:10.2460/javma.2004.225.1549 | Sampling design |
| Martínez Barbabosa I, Ruiz González LA, Gutiérrez Quiroz M, Fernández Presas AM, Vásquez Tsuji O. [Frequency of Toxocara cati eggs in domestic cats in Mexico City and the State of Mexico]. Bol Chil Parasitol. 1997;52: 12–7. | Language |
| Medimond, Jittapalapong S, Krajarng A, Pratchyasakul S, Pinyopanuwat N, Chimnoi W, et al. Molecular epidemiology of Brugia infections of stray cats in Bangkok, Thailand. 2006. pp. 457-+. | Sampling design |
| Meng XZ, Li MY, Lyu C, Qin YF, Zhao ZY, Yang XB, et al. The global prevalence and risk factors of Cryptosporidium infection among cats during 1988-2021: A systematic review and meta-analysis. Microb Pathog. 2021;158: 105096. doi:10.1016/j.micpath.2021.105096 | Sampling design |
| Michalczyk M, Sokół R. [The incidence of internal parasites in dogs and cats as dependent on the level of awareness among owners]. Wiad Parazytol. 2008;54: 245–7. | Unavailable |
| Mitchell D, Reinhard C, Cole S, Stefanovski D, Watson B. Seroprevalence of Toxoplasmosis among Shelter-Housed Felines in a Philadelphia Suburb. ANIMALS. 2022;12. doi:10.3390/ani12162012 | Shelter only |
| Monticello TM, Levy MG, Bunch SE, Fairley RA. Cryptosporidiosis in a feline leukemia virus-positive cat. J Am Vet Med Assoc. 1987;191: 705–6. | Sampling design |
| Moraes L, Neto V, de Oliveira R, Providelo G, Babboni S, Ferreira J, et al. Retrospective and Comparative Study of Giardia sp. Prevalence in Dogs, Cats, and Small Ruminants in Endemic Areas in Different Brazilian States. ACTA SCIENTIAE VETERINARIAE. 2019;47. doi:10.22456/1679-9216.91878 | Cat lifestyles joined |
| Moreira ADS, Baptista CT, Brasil CL, Valente JSS, Bruhn FRP, Pereira DIB. Risk factors and infection due to Cryptosporidium spp. in dogs and cats in southern Rio Grande do Sul. Rev Bras Parasitol Vet. 2018;27: 113–118. doi:10.1590/S1984-296120180012 | Cat lifestyles joined |
| Morgenthal D, Hamel D, Arndt G, Silaghi C, Pfister K, Kempf VA, et al. [Prevalence of haemotropic Mycoplasma spp., Bartonella spp. and Anaplasma phagocytophilum in cats in Berlin/Brandenburg (Northeast Germany)]. Berl Munch Tierarztl Wochenschr. 2012;125: 418–27. | Unavailable |
| Moskvina T, Izrailskaia A, Tsybulsky A. Parasites of stray and client-owned domestic cats in urban areas in Russia during 2000-2015 years. TROPICAL BIOMEDICINE. 2018;35: 267–279. | Sampling design |
| Mtambo MM, Nash AS, Blewett DA, Smith HV, Wright S. Cryptosporidium infection in cats: prevalence of infection in domestic and feral cats in the Glasgow area. Vet Rec. 1991;129: 502–4. | Cat lifestyles joined |
| Naguib D, Gantois N, Desramaut J, Arafat N, Even G, Certad G, et al. Prevalence, Subtype Distribution and Zoonotic Significance of Blastocystis sp. Isolates from Poultry, Cattle and Pets in Northern Egypt. MICROORGANISMS. 2022;10. doi:10.3390/microorganisms10112259 | Cat lifestyles joined |
| Niamnuy N, Kaewthamasorn M, Congpuong K, Phaytanavanh B, Lohsoonthorn V. PREVALENCE AND ASSOCIATED RISK FACTORS OF INTESTINAL PARASITES IN HUMANS AND DOMESTIC ANIMALS ACROSS BORDERS OF THAILAND AND LAO PDR: FOCUS ON HOOKWORM AND THREADWORM. Southeast Asian J Trop Med Public Health. 2016;47: 901–11. | Unavailable |
| Oguz B, Selcin O, Deger M, Bicek K, Ozdal N. A Case Report of Echinococcus granulosus sensu stricto (G1) in a Domestic Cat in Turkey. JOURNAL OF THE HELLENIC VETERINARY MEDICAL SOCIETY. 2021;72: 3529–3534. doi:10.12681/jhvms.29408 | Sampling design |
| Olson ME, Leonard NJ, Strout J. Prevalence and diagnosis of Giardia infection in dogs and cats using a fecal antigen test and fecal smear. Can Vet J. 2010;51: 640–2. | Sampling design |
| Önder Z, Yetişmiş G, Pekmezci D, Delibaşı Kökçü N, Pekmezci GZ, Çiloğlu A, et al. Investigation of Zoonotic Cryptosporidium and Giardia intestinalis Species and Genotypes in Cats (Felis catus). Turkiye Parazitol Derg. 2021;45: 252–256. doi:10.4274/tpd.galenos.2021.46320 | Sampling design |
| Onder Z, Yildirim A, Pekmezci D, Duzlu O, Pekmezci GZ, Ciloglu A, et al. Molecular identification and subtype distribution of Blastocystis sp. in farm and pet animals in Turkey. Acta Trop. 2021;220: 105939. doi:10.1016/j.actatropica.2021.105939 | Cat lifestyles joined |
| Ouchetati I, Ouchene-Khelifi N, Ouchene N, Khelifi M, Dahmani A, Haïf A, et al. Prevalence of Toxoplasma gondii infection among animals in Algeria: A systematic review and meta-analysis. COMPARATIVE IMMUNOLOGY MICROBIOLOGY AND INFECTIOUS DISEASES. 2021;74. doi:10.1016/j.cimid.2020.101603 | Sampling design |
| Pallant L, Barutzki D, Schaper R, Thompson R. The epidemiology of infections with Giardia species and genotypes in well cared for dogs and cats in Germany. PARASITES & VECTORS. 2015;8. doi:10.1186/s13071-014-0615-2 | Sampling design |
| Pallant L, Barutzki D, Schaper R, Thompson RC. The epidemiology of infections with Giardia species and genotypes in well cared for dogs and cats in Germany. Parasit Vectors. 2015;8: 2. doi:10.1186/s13071-014-0615-2 | Sampling design |
| Palmer CS, Traub RJ, Robertson ID, Devlin G, Rees R, Thompson RC. Determining the zoonotic significance of Giardia and Cryptosporidium in Australian dogs and cats. Vet Parasitol. 2008;154: 142–7. doi:10.1016/j.vetpar.2008.02.031 | Sampling design |
| Papadopoulos E, Komnenou A, Thomas A, Ioannidou E, Colella V, Otranto D. Spreading of Thelazia callipaeda in Greece. TRANSBOUNDARY AND EMERGING DISEASES. 2018;65: 248–252. doi:10.1111/tbed.12626 | Sampling design |
| Paulos S, Köster P, de Lucio A, Hernández-de-Mingo M, Cardona G, Fernández-Crespo J, et al. Occurrence and subtype distribution of Blastocystis sp in humans, dogs and cats sharing household in northern Spain and assessment of zoonotic transmission risk. ZOONOSES AND PUBLIC HEALTH. 2018;65: 993–1002. doi:10.1111/zph.12522 | Sampling design |
| Paulos S, Köster PC, de Lucio A, Hernández-de-Mingo M, Cardona GA, Fernández-Crespo JC, et al. Occurrence and subtype distribution of Blastocystis sp. in humans, dogs and cats sharing household in northern Spain and assessment of zoonotic transmission risk. Zoonoses Public Health. 2018;65: 993–1002. doi:10.1111/zph.12522 | Cat lifestyles joined |
| Pennisi M, Persichetti M. Feline leishmaniosis: Is the cat a small dog? VETERINARY PARASITOLOGY. 2018;251: 131–137. doi:10.1016/j.vetpar.2018.01.012 | Sampling design |
| Pereira A, Parreira R, Cristóvao J, Vitale F, Bastien P, Campino L, et al. Leishmania infantum strains from cats are similar in biological properties to canine and human strains. VETERINARY PARASITOLOGY. 2021;298. doi:10.1016/j.vetpar.2021.109531 | Pathogens pooled |
| Pereira PF, Barbosa ADS, Moura APP, Vasconcellos ML, Uchôa CMA, Bastos OMP, et al. Gastrointestinal parasites in stray and shelter cats in the municipality of Rio de Janeiro, Brazil. Rev Bras Parasitol Vet. 2017;26: 383–388. doi:10.1590/S1984-29612017024 | Sampling design |
| Pet’ko B. [Domestic cats as a source of human flea infestations in towns]. Cesk Epidemiol Mikrobiol Imunol. 1993;42: 190–1. | Language |
| Pétavy AF, Prost C, Gevrey J, Gilot B, Deblock S. [Natural infestation of domestic cats (Felis catus L.) by Echinococcus multilocularis Leuckart, 1863 (Cestoda): first case in France detected in peri-urban area]. C R Acad Sci III. 1988;307: 553–6. | Sampling design |
| Pfukenyi DM, Chipunga SL, Dinginya L, Matenga E. A survey of pet ownership, awareness and public knowledge of pet zoonoses with particular reference to roundworms and hookworms in Harare, Zimbabwe. Trop Anim Health Prod. 2010;42: 247–52. doi:10.1007/s11250-009-9413-9 | Sampling design |
| Phoosangwalthong P, Kamyingkird K, Kengradomkij C, Chimnoi W, Odermatt P, Inpankaew T. Molecular Detection and Genetic Characterization of Zoonotic Hookworm in Semi-Domesticated Cats Residing in Monasteries in Bangkok, Thailand. TROPICAL MEDICINE AND INFECTIOUS DISEASE. 2023;8. doi:10.3390/tropicalmed8020122 | Sampling design |
| Phoosangwalthong P, Luong N, Wongwigkan J, Kamyingkird K, Phasuk J, Pattanatanang K, et al. Toxocara canis and Toxocara cati in Stray Dogs and Cats in Bangkok, Thailand: Molecular Prevalence and Risk Factors. PARASITOLOGIA. 2022;2: 88–94. doi:10.3390/parasitologia2020009 | Sampling design |
| Piekara-Stępińska A, Piekarska J, Gorczykowski M. Cryptosporidium spp. in dogs and cats in Poland. Ann Agric Environ Med. 2021;28: 345–347. doi:10.26444/aaem/120467 | Cat lifestyles joined |
| Piekarska J, Bajzert J, Gorczykowski M, Kantyka M, Podkowik M. Molecular identification of Giardia duodenalis isolates from domestic dogs and cats in Wroclaw, Poland. Ann Agric Environ Med. 2016;23: 410–5. doi:10.5604/12321966.1219178 | Sampling design |
| Ponce-Macotela M, Martínez-Gordillo MN, Bermúdez-Cruz RM, Salazar-Schettino PM, Ortega-Pierres G, Ey PL. Unusual prevalence of the Giardia intestinalis A-II subtype amongst isolates from humans and domestic animals in Mexico. Int J Parasitol. 2002;32: 1201–2. doi:10.1016/s0020-7519(02)00086-3 | Sampling design |
| Poulle M, Bastien M, Richard Y, Josse-Dupuis É, Aubert D, Villena I, et al. Detection of Echinococcus multilocularis and other foodborne parasites in fox, cat and dog faeces collected in kitchen gardens in a highly endemic area for alveolar echinococcosis. PARASITE. 2017;24. doi:10.1051/parasite/2017031 | Environmental survey |
| Raeghi S, Sedeghi S. PREVALENCE OF TOXOPLASMA GONDII ANTIBODIES IN CATS IN URMIA, NORTHWEST OF IRAN. JOURNAL OF ANIMAL AND PLANT SCIENCES. 2011;21: 132–134. | Cat lifestyles joined |
| Ramírez-Ocampo S, Cotte-Alzate JD, Escobedo ÁA, Rodríguez-Morales AJ. Prevalence of zoonotic and non-zoonotic genotypes of Giardia intestinalis in cats: a systematic review and meta-analysis. Infez Med. 2017;25: 326–338. | Sampling design |
| Rappeti J, Mascarenhas C, Perera S, Müller G, Grecco F, da Silva L, et al. Dioctophyme renale (Nematoda: Enoplida) in domestic dogs and cats in the extreme south of Brazil. REVISTA BRASILEIRA DE PARASITOLOGIA VETERINARIA. 2017;26: 119–121. doi:10.1590/S1984-29612016072 | Sampling design |
| Ratzlaff F, Osmari V, da Silva D, Vasconcellos J, Pötter L, Fernandes F, et al. Identification of infection by Leishmania spp. in wild and domestic animals in Brazil: a systematic review with meta-analysis (2001-2021). PARASITOLOGY RESEARCH. 2023;122: 1605–1619. doi:10.1007/s00436-023-07862-y | Sampling design |
| Raust P, Legros F. [First record in French Polynesia of an hepatic fluke of the domestic cat, Platynosomum fastosum Kossack, 1910 (author’s transl)]. Ann Parasitol Hum Comp. 1980;55: 615–8. | Sampling design |
| Rendón-Franco E, Romero-Callejas E, Villanueva-García C, Osorio-Sarabia D, Muñoz-García CI. Cross transmission of gastrointestinal nematodes between captive neotropical felids and feral cats. J Zoo Wildl Med. 2013;44: 936–40. doi:10.1638/2013-0015R2.1 | Sampling design |
| Rep BH. Intestinal helminths in dogs and cats on the Antillian Islands Aruba, Curaçao and Bonaire. Trop Geogr Med. 1975;27: 317–23. | Before 1980 |
| Reperant L, Hegglin D, Tanner I, Fischer C, Deplazes P. Rodents as shared indicators for zoonotic parasites of carnivores in urban environments. PARASITOLOGY. 2009;136: 329–337. doi:10.1017/S0031182008005428 | Sampling design |
| Rezende HH, Avelar JB, Storchilo HR, Vinaud MC, de Castro AM. Evaluation of the accuracy of parasitological techniques for the diagnosis of intestinal parasites in cats. Rev Bras Parasitol Vet. 2015;24: 471–4. doi:10.1590/S1984-29612015069 | Sampling design |
| Robben SR, le Nobel WE, Döpfer D, Hendrikx WM, Boersema JH, Fransen F, et al. [Infections with helminths and/or protozoa in cats in animal shelters in the Netherlands]. Tijdschr Diergeneeskd. 2004;129: 2–6. | Unavailable |
| Rödl P. [The health impact of stray and wild cats in the human environment]. Cesk Epidemiol Mikrobiol Imunol. 1992;41: 169–73. | Sampling design |
| Rodriguez-Vivas R, Salazar-Grosskelwing E, Ojeda-Chi M, Flota-Burgos G, Solano-Barquero A, Trinidad-Martínez I, et al. First morphological and molecular report of Lagochilascaris minor (Nematoda, Ascarididae) in a domestic cat from Veracruz, Mexico. VETERINARY PARASITOLOGY- REGIONAL STUDIES AND REPORTS. 2023;37. doi:10.1016/j.vprsr.2022.100823 | Sampling design |
| Romero JR, Led JE. [A new case of Lagochilascaris major (Leiper 1910) in the Argentine Republic parasitizing the cat (Felis catus domesticus)]. Zentralbl Veterinarmed B. 1985;32: 575–82. | Cat lifestyles joined |
| Romero-Alvarez D, Valverde-Muñoz G, Calvopina M, Rojas M, Cevallos W, Kumazawa H, et al. Liver fluke infections by Amphimerus sp. (Digenea: Opisthorchiidae) in definitive and fish intermediate hosts in Manabí province, Ecuador. PLoS Negl Trop Dis. 2020;14: e0008286. doi:10.1371/journal.pntd.0008286 | Sampling design |
| Rostami A, Riahi S, Omrani V, Wang T, Hofmann A, Mirzapour A, et al. Global Prevalence Estimates of Toxascaris leonina Infection in Dogs and Cats. PATHOGENS. 2020;9. doi:10.3390/pathogens9060503 | Sampling design |
| Rostami A, Sepidarkish M, Ma G, Wang T, Ebrahimi M, Fakhri Y, et al. Global prevalence of Toxocara infection in cats. TOXOCARA AND TOXOCARIASIS. 2020. pp. 615–639. doi:10.1016/bs.apar.2020.01.025 | Sampling design |
| Ruan Y, Xu X, He Q, Li L, Guo J, Bao J, et al. The largest meta-analysis on the global prevalence of microsporidia in mammals, avian and water provides insights into the epidemic features of these ubiquitous pathogens. PARASITES & VECTORS. 2021;14. doi:10.1186/s13071-021-04700-x | Sampling design |
| Samorek-Pierog M, Cencek T, Labuc E, Pac-Sosinska M, Pierog M, Korpysa-Dzirba W, et al. Occurrence of Eucoleus aerophilus in wild and domestic animals: a systematic review and meta-analysis. PARASITES & VECTORS. 2023;16. doi:10.1186/s13071-023-05830-0 | Sampling design |
| Sargent KD, Morgan UM, Elliot A, Thompson RC. Morphological and genetic characterisation of Cryptosporidium oocysts from domestic cats. Vet Parasitol. 1998;77: 221–7. doi:10.1016/s0304-4017(98)00122-8 | Sampling design |
| Sarvi S, Daryani A, Sharif M, Rahimi MT, Kohansal MH, Mirshafiee S, et al. Zoonotic intestinal parasites of carnivores: A systematic review in Iran. Vet World. 2018;11: 58–65. doi:10.14202/vetworld.2018.58-65 | Sampling design |
| Schenone H. [Human parasitic diseases which may be caused or transmitted by domestic pets in Chile]. Bol Chil Parasitol. 1987;42: 16–23. | Sampling design |
| Schuster R, Heidecke D, Schierhorn K. [Contributions to the parasite fauna of local hosts. 10. On the endoparasitic fauna of Felis silvestris]. Appl Parasitol. 1993;34: 113–20. | Language |
| Schuster R, Kaufmann A, Hering S. [Investigations on the endoparasitic fauna of domestic cats in eastern Brandenburg]. Berl Munch Tierarztl Wochenschr. 1997;110: 48–50. | Language |
| Scorza AV, Tyrrell P, Wennogle S, Chandrashekar R, Lappin MR. Experimental infection of cats with Cryptosporidium felis. J Feline Med Surg. 2022;24: 1060–1064. doi:10.1177/1098612X211053477 | Sampling design |
| Scorza V, Willmott A, Gunn-Moore D, Lappin MR. Cryptosporidium felis in faeces from cats in the UK. Vet Rec. 2014;174: 609. doi:10.1136/vr.102205 | Sampling design |
| Seiler M, Eckert J, Wolff K. [Giardia and other intestinal parasites of dogs and cats in Switzerland]. Schweiz Arch Tierheilkd. 1983;125: 137–48. | Unavailable |
| Sergiev VP, Uspenskiĭ AV, Gorokhov VV, Romanenko NA, Novosel’tsev GI, Peshkov RA, et al. [The current canine and feline parasitic diseases situation in the megapolis of Moscow]. Med Parazitol (Mosk). 2007; 17–20. | Sampling design |
| Serra CM, Uchôa CM, Coimbra RA. [Parasitological study with faecal samples of stray and domiciliated cats (Felis catus domesticus) from the Metropolitan Area of Rio de Janeiro, Brazil]. Rev Soc Bras Med Trop. 2003;36: 331–4. doi:10.1590/s0037-86822003000300003 | Language |
| Sevá ADP, Pena HFJ, Nava A, Sousa AO, Holsback L, Soares RM. Endoparasites in domestic animals surrounding an Atlantic Forest remnant, in São Paulo State, Brazil. Rev Bras Parasitol Vet. 2018;27: 13–19. doi:10.1590/S1984-29612017078 | Sampling design |
| Shams M, Shamsi L, Yousefi A, Sadrebazzaz A, Asghari A, Mohammadi-Ghalehbin B, et al. Current global status, subtype distribution and zoonotic significance of Blastocystis in dogs and cats: a systematic review and meta-analysis. Parasit Vectors. 2022;15: 225. doi:10.1186/s13071-022-05351-2 | Sampling design |
| Shaw J, Dunsmore J, Jakob-Hoff R. Prevalence of some gastrointestinal parasites in cats in the Perth area. Aust Vet J. 1983;60: 151–2. doi:10.1111/j.1751-0813.1983.tb05933.x | Sampling design |
| Shukla R, Giraldo P, Kraliz A, Finnigan M, Sanchez AL. Cryptosporidium spp. and other zoonotic enteric parasites in a sample of domestic dogs and cats in the Niagara region of Ontario. Can Vet J. 2006;47: 1179–84. | Sampling design |
| Shukullari E, Hamel D, Rapti D, Pfister K, Visser M, Winter R, et al. Parasites and vector-borne diseases in client-owned dogs in Albania. Intestinal and pulmonary endoparasite infections. Parasitol Res. 2015;114: 4579–90. doi:10.1007/s00436-015-4704-8 | Sampling design |
| Silva ACDS, Paschoal ATP, Bernardes JC, Matos AMRN, Balbino LS, Santomauro RA, et al. Parasites in road-killed wild felines from North of Paraná state, Brazil. Rev Bras Parasitol Vet. 2021;30: e016320. doi:10.1590/S1984-296120201090 | Sampling design |
| Simonato G, Danesi P, di Regalbono A, Dotto G, Tessarin C, Pietrobelli M, et al. Surveillance of Zoonotic Parasites in Animals Involved in Animal-Assisted Interventions (AAIs). INTERNATIONAL JOURNAL OF ENVIRONMENTAL RESEARCH AND PUBLIC HEALTH. 2020;17. doi:10.3390/ijerph17217914 | Sampling design |
| Smailova AN. [The current status of the Turgai focus of opisthorchiasis]. Med Parazitol (Mosk). 1990; 42–3. | Sampling design |
| Šmigová J, Papajová I, Šoltys J, Pipiková J, Šmiga Ľ, Šnábel V, et al. The occurence of endoparasites in Slovakian household dogs and cats. Vet Res Commun. 2021;45: 243–249. doi:10.1007/s11259-021-09804-4 | Pathogens pooled |
| Sohn WM, Han ET, Chai JY. Acanthotrema felis n. sp. (Digenea: Heterophyidae) from the small intestine of stray cats in the Republic of Korea. J Parasitol. 2003;89: 154–8. doi:10.1645/0022-3395(2003)089%5B0154:AFNSDH%5D2.0.CO;2 | Cat lifestyles joined |
| Soleimani A, Mohebali M, Gholizadeh S, Bozorgomid A, Shafiei R, Raeghi S. Molecular and serological evaluation of visceral leishmaniasis in domestic dogs and cats in Maragheh County, north-west of Iran, 2018-2021. VETERINARY MEDICINE AND SCIENCE. 2022;8: 1898–1903. doi:10.1002/vms3.846 | Cat lifestyles joined |
| Soroushianfar M, Sadr S, Sazmand A, Dianaty S, Khedri J, Schuster RK, et al. Gastrointestinal parasites of cats in the Middle East (2000-2023): A literature review. Parasitol Int. 2024;102: 102919. doi:10.1016/j.parint.2024.102919 | Sampling design |
| Souza SL, Gennari SM, Richtzenhain LJ, Pena HF, Funada MR, Cortez A, et al. Molecular identification of Giardia duodenalis isolates from humans, dogs, cats and cattle from the state of São Paulo, Brazil, by sequence analysis of fragments of glutamate dehydrogenase (gdh) coding gene. Vet Parasitol. 2007;149: 258–64. doi:10.1016/j.vetpar.2007.08.019 | Sampling design |
| Stalliviere F, Bellato V, de Souza A, Sartor A, de Moura A, Dalla Rosa L. Ectoparasites and intestinal helminths in Felis catus domesticus from Lages city, SC, Brazil and social-economical and cultural aspects of owners of family pets. REVISTA BRASILEIRA DE PARASITOLOGIA VETERINARIA. 2009;18: 26–31. doi:10.4322/rbpv.01804005 | Language |
| Stalliviere FM, Bellato V, Souza AP, Sartor AA, Moura AB, Rosa LD. [Ectoparasites and intestinal helminths in Felis catus domesticus from Lages city, SC, Brazil and social-economical and cultural aspects of owners of family pets]. Rev Bras Parasitol Vet. 2009;18: 26–31. doi:10.4322/rbpv.01804005 | Language |
| Styles TJ, Evans DS. Intestinal parasites of dogs and cats in Schenectady County. N Y State J Med. 1971;71: 2755–7. | Unavailable |
| Suergec E, Güvendi M, Karakavuk M, Alak S, Döskaya A, Ün C, et al. Genotyping of Enterocytozoon bieneusi isolates detected in stray cats of Izmir, Turkiye. PARASITOLOGY RESEARCH. 2023;122: 2729–2735. doi:10.1007/s00436-023-07974-5 | Sampling design |
| Supperer R, Hinaidy HK. [Parasitic infestation of dogs and cats in Austria]. Dtsch Tierarztl Wochenschr. 1986;93: 383–6. | Language |
| Sursal N, Simsek E, Yildiz K. Occurrence and First Molecular Characterization of Cryptosporidium felis in a Cat in Turkey. KAFKAS UNIVERSITESI VETERINER FAKULTESI DERGISI. 2020;26: 833–837. doi:10.9775/kvfd.2020.24453 | Sampling design |
| Svoboda M. [Incidence of antibodies to Toxoplasma gondii in cats from Brno and the surrounding area]. Vet Med (Praha). 1988;33: 45–54. | Language |
| Swan JM, Thompson RC. The prevalence of Giardia in dogs and cats in Perth, Western Australia. Aust Vet J. 1986;63: 110–2. doi:10.1111/j.1751-0813.1986.tb07676.x | Sampling design |
| Sweet S, Szlosek D, McCrann D, Coyne M, Kincaid D, Hegarty E. Retrospective analysis of feline intestinal parasites: trends in testing positivity by age, USA geographical region and reason for veterinary visit. Parasit Vectors. 2020;13: 473. doi:10.1186/s13071-020-04319-4 | Sampling design |
| Tabakaeva T, Galkina I, Tabakaev A, Shchelkanov M. Anthropozoonotic parasitoses of dogs and cats in the urban ecosystem of Vladivostok, Russia. SOUTH OF RUSSIA-ECOLOGY DEVELOPMENT. 2024;19. doi:10.18470/1992-1098-2024-2-2 | Language |
| Taghipour A, Ghodsian S, Shajarizadeh M, Sharbatkhori M, Khazaei S, Mirjalali H. Global prevalence of microsporidia infection in cats: A systematic review and meta-analysis of an emerging zoonotic pathogen. PREVENTIVE VETERINARY MEDICINE. 2021;188. doi:10.1016/j.prevetmed.2021.105278 | Sampling design |
| Taghipour A, Khazaei S, Ghodsian S, Shajarizadeh M, Olfatifar M, Foroutan M, et al. Global prevalence of Cryptosporidium spp. in cats: A systematic review and meta-analysis. Res Vet Sci. 2021;137: 77–85. doi:10.1016/j.rvsc.2021.04.015 | Sampling design |
| Tamponi C, Varcasia A, Pinna S, Melis E, Melosu V, Zidda A, et al. Endoparasites detected in faecal samples from dogs and cats referred for routine clinical visit in Sardinia, Italy. Vet Parasitol Reg Stud Reports. 2017;10: 13–17. doi:10.1016/j.vprsr.2017.07.001 | Pathogens compiled |
| Tang F, Pan Z, Li D, Ma L, Xiong Y, Lu C. [Isolation and characterization of a Streptococcus suis serotype 9 from a wild cat]. Wei Sheng Wu Xue Bao. 2016;56: 275–82. | Sampling design |
| Tangkawattana S, Tangkawattana P. Reservoir Animals and Their Roles in Transmission of Opisthorchis viverrini. ASIATIC LIVER FLUKE - FROM BASIC SCIENCE TO PUBLIC HEALTH, PT A. 2018. pp. 69-+. doi:10.1016/bs.apar.2018.05.003 | Sampling design |
| Thompson RC, Meloni BP, Hopkins RM, Deplazes P, Reynoldson JA. Observations on the endo- and ectoparasites affecting dogs and cats in aboriginal communities in the north-west of Western Australia. Aust Vet J. 1993;70: 268–70. doi:10.1111/j.1751-0813.1993.tb08050.x | Sampling design |
| Tiyo R, Guedes T, Falavigna D, Falavigna-Guilherme A. Seasonal contamination of public squares and lawns by parasites with zoonotic potential in southern Brazil. JOURNAL OF HELMINTHOLOGY. 2008;82: 1–6. doi:10.1017/S0022149X07870829 | Environmental survey |
| Torres P, Navarrete N, Martin R, Contreras A. [Sarcocystis sp. in the diaphragm of a domestic cat (Felis catus) from Valdivia, Chile]. Bol Chil Parasitol. 1996;51: 30–2. | Sampling design |
| Torrico KJ, Santos KR, Martins T, Paz E Silva FM, Takahira RK, Lopes RS. [Occurrence of gastrointestinal parasites in dogs and cats in the laboratory of routine of parasitic diseases FMVZ/Unesp-Botucatu, SP]. Rev Bras Parasitol Vet. 2008;17 Suppl 1: 182–3. | Cat lifestyles joined |
| Tsianakas P, Polack B, Pinquier L, Levy Klotz B, Prost-Squarcioni C. [Cheyletiella dermatitis: an uncommon cause of vesiculobullous eruption]. Ann Dermatol Venereol. 2000;127: 826–9. | Sampling design |
| Tuna G, Aksoy T, Ay C. Occurrence of Dirofilaria immitis in cats from the Aegean region in Turkey. MEDYCYNA WETERYNARYJNA-VETERINARY MEDICINE-SCIENCE AND PRACTICE. 2022;78: 631–634. doi:10.21521/mw.6706 | Sole pathogen not zoonotic |
| Tzannes S, Batchelor DJ, Graham PA, Pinchbeck GL, Wastling J, German AJ. Prevalence of Cryptosporidium, Giardia and Isospora species infections in pet cats with clinical signs of gastrointestinal disease. J Feline Med Surg. 2008;10: 1–8. doi:10.1016/j.jfms.2007.05.006 | Sampling design |
| Udonsom R, Prasertbun R, Mahittikorn A, Mori H, Changbunjong T, Komalamisra C, et al. Blastocystis infection and subtype distribution in humans, cattle, goats, and pigs in central and western Thailand. Infect Genet Evol. 2018;65: 107–111. doi:10.1016/j.meegid.2018.07.007 | Sampling design |
| Ugarte C, Thomas D, Gasser R, Hu M, Scott I, Collett M. Spirometra erinacei/S. erinaceieuropaei in a feral cat in Manawatu with chronic intermittent diarrhoea. NEW ZEALAND VETERINARY JOURNAL. 2005;53: 347–351. doi:10.1080/00480169.2005.36573 | Sampling design |
| Vahedi SM, Jamshidi S, Shayan P, Bokaie S, Ashrafi Tamai I, Javanmard E, et al. Intestinal microsporidia infection among cat owners and non-pet owners in Iran: a case-control study. Parasitol Res. 2020;119: 1903–1913. doi:10.1007/s00436-020-06690-8 | Sampling design |
| Vasilopulos R, Rickard L, Mackin A, Pharr G, Huston C. Genotypic analysis of Giardia duodenalis in domestic cats. JOURNAL OF VETERINARY INTERNAL MEDICINE. 2007;21: 352–355. | Sampling design |
| Vasilopulos RJ, Mackin AJ, Rickard LG, Pharr GT, Huston CL. Prevalence and factors associated with fecal shedding of Giardia spp. in domestic cats. J Am Anim Hosp Assoc. 2006;42: 424–9. doi:10.5326/0420424 | Unavailable |
| Visco RJ, Corwin RM, Selby LA. Effect of age and sex on the prevalence of intestinal parasitism in cats. J Am Vet Med Assoc. 1978;172: 797–800. | Before 1980 |
| Vrhovec MG, Alnassan AA, Pantchev N, Bauer C. Is there any change in the prevalence of intestinal or cardiopulmonary parasite infections in companion animals (dogs and cats) in Germany between 2004-2006 and 2015-2017? An assessment of the impact of the first ESCCAP guidelines. Vet Parasitol. 2022;312: 109836. doi:10.1016/j.vetpar.2022.109836 | Sampling design |
| West L, Wise W, Golway P, Johnson R, Moore D, Toups G, et al. Internal parasites in cats. Mod Vet Pract. 1975;56: 861–3. | Before 1980 |
| Wilson-Hanson SL, Prescott CW. A survey for parasites in cats. Aust Vet J. 1982;59: 194. doi:10.1111/j.1751-0813.1982.tb16009.x | Sampling design |
| Wójcik-Fatla A, Sroka J, Zając V, Sawczyn-Domańska A, Kloc A, Zwoliński J, et al. Potential sources of infection with selected zoonotic agents in the veterinary work environment - pilot studies. Ann Agric Environ Med. 2020;27: 146–150. doi:10.26444/aaem/115363 | Sampling design |
| Wolff K, Eckert J. [Giardia infection of dogs and cats and its possible significance for man]. Berl Munch Tierarztl Wochenschr. 1979;92: 479–84. | Before 1980 |
| Yamamoto N, Kon M, Saito T, Maeno N, Koyama M, Sunaoshi K, et al. [Prevalence of intestinal canine and feline parasites in Saitama Prefecture, Japan]. Kansenshogaku Zasshi. 2009;83: 223–8. doi:10.11150/kansenshogakuzasshi.83.223 | Language |
| Yaman M, Ayaz E, Gül A, Muz MN. [Investigation of helminth infections of cats and dogs in the Hatay province]. Turkiye Parazitol Derg. 2006;30: 200–4. | Unavailable |
| Yan QR, Yan T, Zhou XM, Li YS, Zhu CC, Shi LB, et al. [Epidemiological survey on the infection of Paragonimus westermani in Jiangxi Province]. Zhongguo Ji Sheng Chong Xue Yu Ji Sheng Chong Bing Za Zhi. 2004;22: 250–2. | Sampling design |
| Yang Y, Liang H. Prevalence and Risk Factors of Intestinal Parasites in Cats from China. Biomed Res Int. 2015;2015: 967238. doi:10.1155/2015/967238 | Cat lifestyles joined |
| Yoshiuchi R, Matsubayashi M, Kimata I, Furuya M, Tani H, Sasai K. Survey and molecular characterization of Cryptosporidium and Giardia spp. in owned companion animal, dogs and cats, in Japan. VETERINARY PARASITOLOGY. 2010;174: 313–316. doi:10.1016/j.vetpar.2010.09.004 | Pathogens pooled |
| Zhang J, Qin Y, Shen Y, Wang Y, Cao J, Su Y, et al. [Prevalence and genotyping of Cryptosporidium spp. and Giardia lamblia in dogs and cats from a pet hospital in Shanghai Municipality]. Zhongguo Xue Xi Chong Bing Fang Zhi Za Zhi. 2023;35: 258–262. doi:10.16250/j.32.1374.2023098 | Language |
| Zhong Y, Zhou Z, Deng L, Liu H, Zhong Z, Ma X, et al. Prevalence and new genotypes of Enterocytozoon bieneusi in sheltered dogs and cats in Sichuan province, southwestern China. PARASITE. 2021;28. doi:10.1051/parasite/2021029 | Shelter only |
| Zhu X, Jacobs D, Chilton N, Sani R, Cheng N, Gasser R. Molecular characterization of a Toxocara variant from cats in Kuala Lumpur, Malaysia. PARASITOLOGY. 1998;117: 155–164. doi:10.1017/S0031182098002856 | Sampling design |
| Zimmer K, Bogantes JC, Herbst W, Räther W. [Poxvirus infections in a cat and its owner]. Tierarztl Prax. 1991;19: 423–7. | Sampling design |
