# Supplementary material for: Outdoor roaming of owned cats elevates risk of zoonotic pathogen exposure: A global synthesis
Source: PLoS Pathog. 2026 Apr 20;22(4):e1014160. doi: 10.1371/journal.ppat.1014160 (PMC13128103; doi:10.1371/journal.ppat.1014160)
Supplement: S5 Table — (DOCX) [file ppat.1014160.s005.docx]

**S5 Table** Posterior means and odds ratios (with 95% credible intervals) for cat lifestyle contrasts in all-pathogen models restricted to studies conducted after 2000 and after 2010.

| **Year** | **Contrast** | **β** | **OR** |
| --- | --- | --- | --- |
| 2000+ | Outdoor vs Indoor | 1.07 (0.766, 1.38) | 2.92 (2.15, 3.99) |
| 2000+ | Indoor vs Outdoor | -1.07 (-1.38, -0.766) | 0.343 (0.251, 0.465) |
| 2000+ | Feral vs Indoor | 1.21 (0.886, 1.52) | 3.35 (2.43, 4.59) |
| 2000+ | Indoor vs Feral | -1.21 (-1.52, -0.886) | 0.299 (0.218, 0.412) |
| 2000+ | Feral vs Outdoor | 0.136 (-0.143, 0.404) | 1.15 (0.867, 1.5) |
| 2000+ | Outdoor vs Feral | -0.136 (-0.404, 0.143) | 0.872 (0.667, 1.15) |
| 2010+ | Outdoor vs Indoor | 1.12 (0.778, 1.46) | 3.05 (2.18, 4.29) |
| 2010+ | Indoor vs Outdoor | -1.12 (-1.46, -0.778) | 0.327 (0.233, 0.459) |
| 2010+ | Feral vs Indoor | 1.19 (0.833, 1.56) | 3.28 (2.3, 4.74) |
| 2010+ | Indoor vs Feral | -1.19 (-1.56, -0.833) | 0.305 (0.211, 0.435) |
| 2010+ | Feral vs Outdoor | 0.0721 (-0.227, 0.382) | 1.07 (0.797, 1.46) |
| 2010+ | Outdoor vs Feral | -0.0721 (-0.382, 0.227) | 0.93 (0.683, 1.25) |
