# Supplementary figures and images for: Outdoor roaming of owned cats elevates risk of zoonotic pathogen exposure: A global synthesis
Source: PLoS Pathog. 2026 Apr 20;22(4):e1014160. doi: 10.1371/journal.ppat.1014160 (PMC13128103; doi:10.1371/journal.ppat.1014160)

**Figure S1.** PRISMA flow diagram of study selection for zoonotic pathogens in domestic cats.

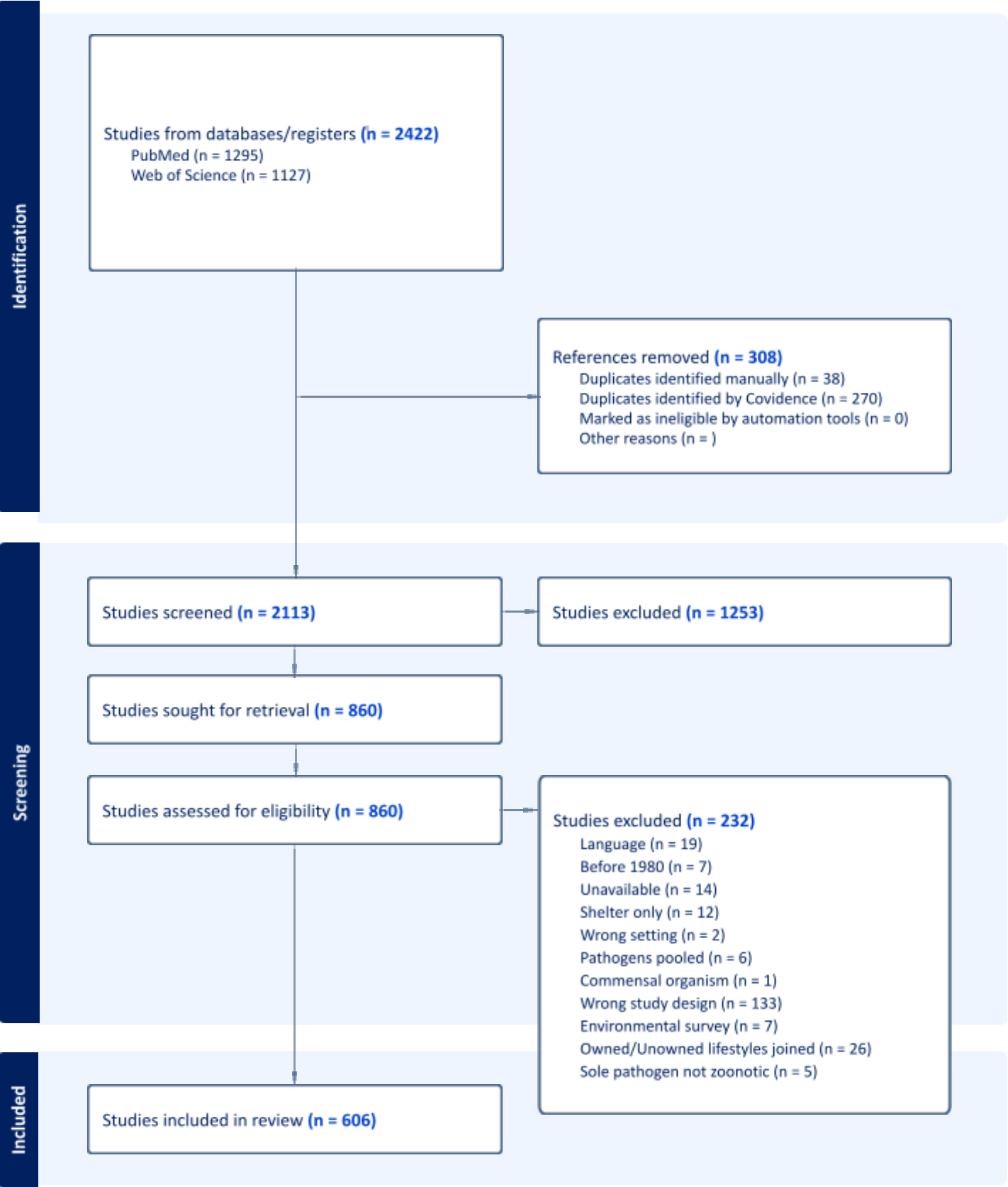

Supplement: S1 Fig — (PDF) [file ppat.1014160.s006.pdf]
