# Supplementary material for: Outdoor roaming of owned cats elevates risk of zoonotic pathogen exposure: A global synthesis
Source: PLoS Pathog. 2026 Apr 20;22(4):e1014160. doi: 10.1371/journal.ppat.1014160 (PMC13128103; doi:10.1371/journal.ppat.1014160)
Supplement: S2 Fig — Plots show posterior samples for the intercept and cat lifestyle effects (outdoor-owned and feral cats), demonstrating adequate mixing and stationarity after burn-in. (PDF) [file ppat.1014160.s007.pdf]

Figure S2. Trace plots for key fixed effects from Bayesian mixed-effects models.
Panels (a–h) show posterior samples for the intercept and cat lifestyle effects (outdoor-owned and feral cats) from models evaluating infection risk across all pathogens combined (a) and individual pathogens: Ancyclostoma (b), Bartonella (c), Cryptosporidium (d), Giardia (e), Leptospira (f), *Toxocara cati* (g), and *Toxoplasma gondii* (h).

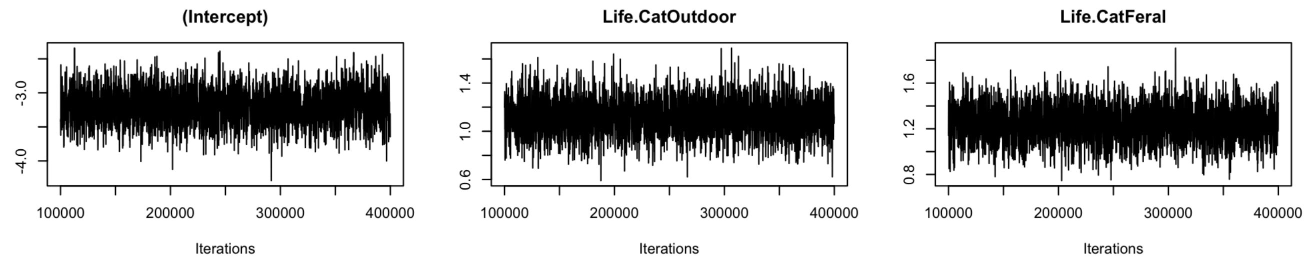

Figure S2a. Trace plots for key fixed effects from the Bayesian mixed-effects model evaluating infection risk across all zoonotic pathogens combined. Plots show posterior samples for the intercept and cat lifestyle effects (outdoor-owned and feral cats), demonstrating adequate mixing and stationarity after burn-in.

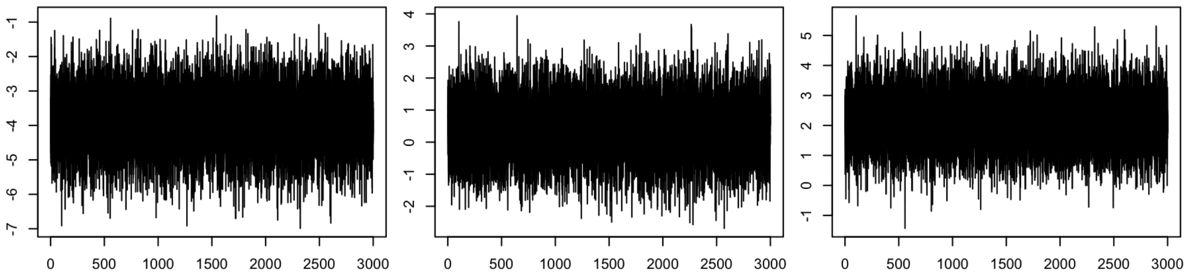

Figure S2b. Trace plots for key fixed effects from the Bayesian mixed-effects model evaluating infection risk for Ancyclostoma. Plots show posterior samples for the intercept and cat lifestyle effects (outdoor-owned and feral cats), demonstrating adequate mixing and stationarity after burn-in.

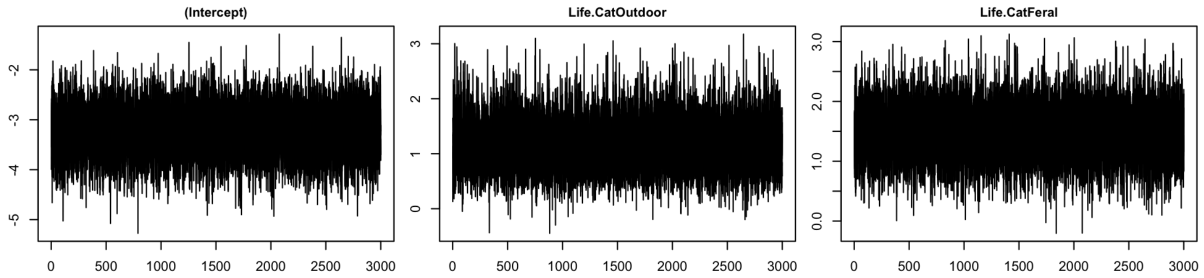

Figure S2c. Trace plots for key fixed effects from the Bayesian mixed-effects model evaluating *Bartonella* infection risk. Plots show posterior samples for the intercept and cat lifestyle effects (outdoor-owned and feral cats), demonstrating adequate mixing and stationarity after burn-in.

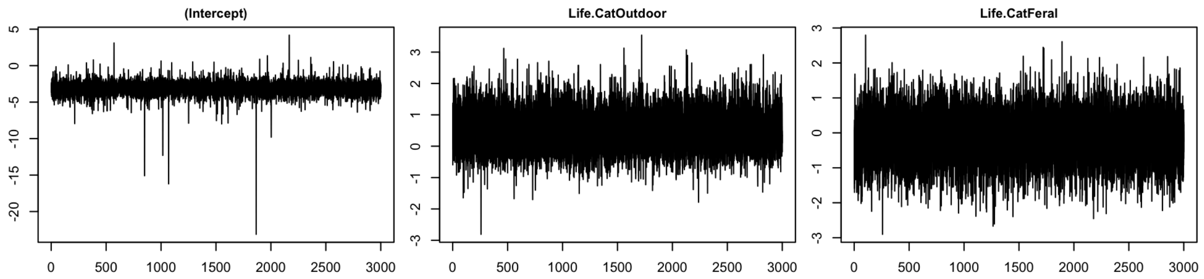

Figure S2d. Trace plots for key fixed effects from the Bayesian mixed-effects model evaluating *Cryptosporidium* infection risk. Plots show posterior samples for the intercept and cat lifestyle effects (outdoor-owned and feral cats), demonstrating adequate mixing and stationarity after burn-in.

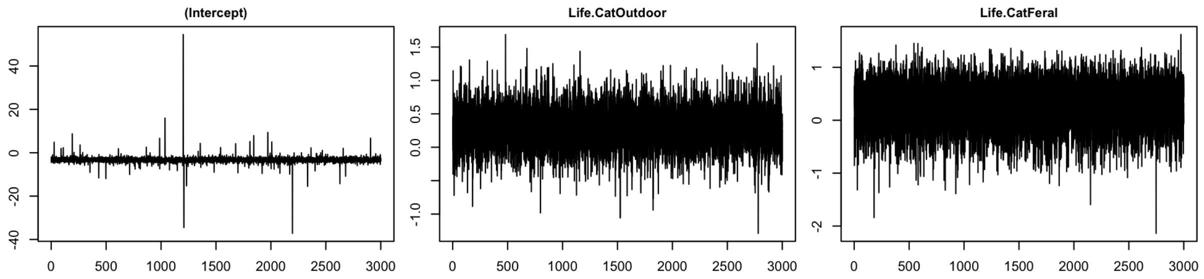

Figure S2e. Trace plots for key fixed effects from the Bayesian mixed-effects model evaluating *Giardia* infection risk. Plots show posterior samples for the intercept and cat lifestyle effects (outdoor-owned and feral cats), demonstrating adequate mixing and stationarity after burn-in.

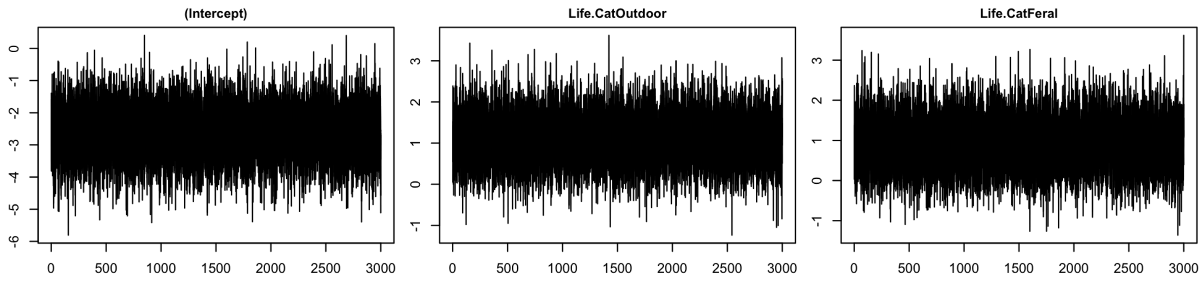

Figure S2f. Trace plots for key fixed effects from the Bayesian mixed-effects model evaluating *Leptospira* infection risk. Plots show posterior samples for the intercept and cat lifestyle effects (outdoor-owned and feral cats), demonstrating adequate mixing and stationarity after burn-in.

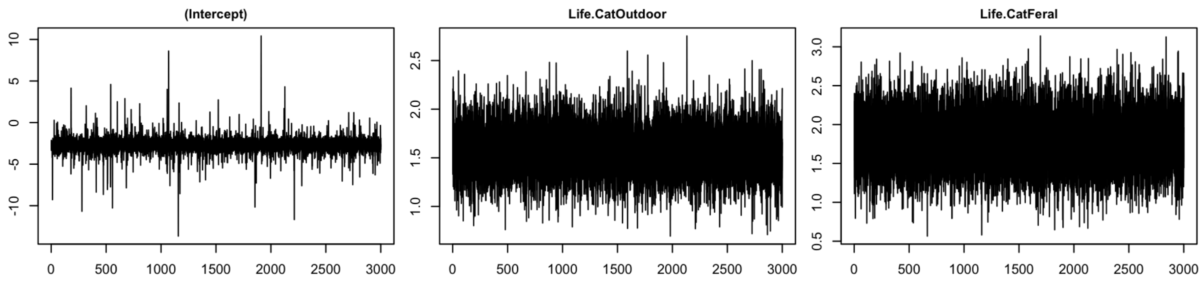

Figure S2g. Trace plots for key fixed effects from the Bayesian mixed-effects model evaluating *Toxocara cati* infection risk. Plots show posterior samples for the intercept and cat lifestyle effects (outdoor-owned and feral cats), demonstrating adequate mixing and stationarity after burn-in.

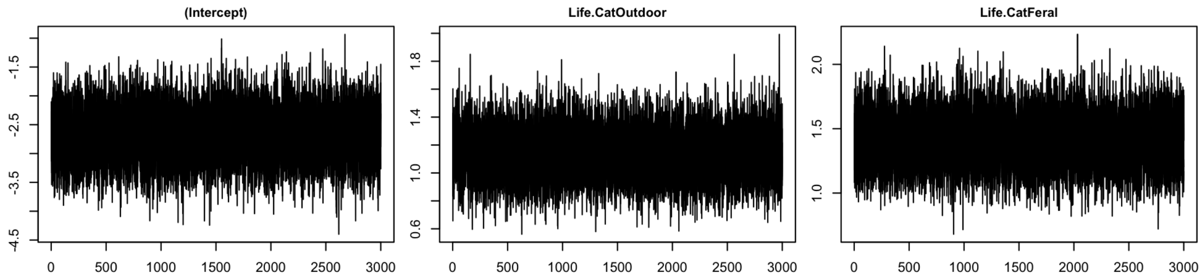

Figure S2h. Trace plots for key fixed effects from the Bayesian mixed-effects model evaluating *Toxoplasma gondii* infection risk. Plots show posterior samples for the intercept and cat lifestyle effects (outdoor-owned and feral cats), demonstrating adequate mixing and stationarity after burn-in.
