# Supplementary material for: Outdoor roaming of owned cats elevates risk of zoonotic pathogen exposure: A global synthesis
Source: PLoS Pathog. 2026 Apr 20;22(4):e1014160. doi: 10.1371/journal.ppat.1014160 (PMC13128103; doi:10.1371/journal.ppat.1014160)
Supplement: Attachment — Submitted filename: 25-02235R1_ Reviewer_Response_PPATHOGENS-D-25-02235.pdf [file ppat.1014160.s011.pdf]

Reviewers' Comments:

Reviewer's Responses to Questions

Part I - Summary

Please use this section to discuss strengths/weaknesses of study, novelty/significance, general execution and scholarship.

Reviewer #1: This manuscript addresses a highly relevant One Health issue through an impressive synthesis of global-scale data. The authors compiled a remarkably large dataset (174,178 individuals across 88 countries), analyzed pathogen prevalence under different management regimes, and reached conclusions with direct implications for public health, wildlife ecology, and responsible pet ownership policy. The writing is clear, the importance of the issue is well motivated, and the statistical approach is generally appropriate. The study has strong potential for publication after some revisions, particularly regarding methodological transparency and the way certain assumptions and limitations are framed.

Reviewer #2: This manuscript summarizes results of a systematic meta-analysis of pathogen prevalence in domestic cats and compares disease risk in owned, roaming cats to indoor and feral cats. The research paper is well-designed, organized and covers an important topic with significance to cat, human and wildlife health. It elucidates information from the literature that should be used to educate owners and inform management of free-roaming cats.

Reviewer #3: Overall, this is an important area to research. The strengths include a well written paper with few grammar errors, and using moderately recent references.

3.1 The main concern is that this paper provides too vague of a conclusion while attempting to analyze too many infectious species and countries.

3.2 The dataset does not clearly state about how old the included studies are, as this could affect the identifiable pathogens (e.g., novel or more recently identified pathogens, diagnostic method developments which would impact the ability to identify pathogens). To expand, there would be a major difference of diagnostic ability and reliability with identifying helminths between PCR and manual microscopic evaluation, even between human readers (i.e., a trained clinical pathologist versus a recent graduate who may not be as experienced).

3.3 The dataset of the identified pathogens is also lacking, particular the indoor pathogens, which would be a bias against indoor cats. Out of the all the pathogens in table S1, only 12 were analyzed of the indoor population. There is a difference between unreported and negative, so it would be unfair to use the lack of indoor pathogens as a comparison.

3.4 There are also major assumptions that weaken the study. The first is the assumption is that indoor cats are being provided with appropriate veterinary care.

3.5 Secondarily, the risk for each pathogen is equal globally, when there is a significant variation of pathogen risk between countries and even between states/provinces.

3.6 While understanding the importance of outdoor cats and zoonotic spillover is needed in the current literature, I unfortunately believe that this study is over-generalizing the population while not providing sufficient data to support the claims.

Comment 3.1 : *"The main concern is that this paper provides too vague of a conclusion while attempting to analyze too many infectious species and countries..."*

3.1 Response: We appreciate the reviewer's perspective. The objective of this study was to conduct a macroecological synthesis to test a broad, global driver of zoonotic pathogen risk rather than to provide a clinical assessment of the absolute risk of specific pathogens in specific locales. By including a wide range of species and countries, we demonstrated that the relative risk associated with outdoor roaming is a robust association across multiple zoonotic pathogens and geographic boundaries. We believe this breadth of conclusion spanning pathogens and countries is a primary strength, as it provides a generalized framework for public health and veterinary policy.

Comment 3.2: "The dataset does not clearly state about how old the included studies are... there would be a major difference of diagnostic ability... between PCR and manual microscopic evaluation..."

3.2 Response: This is an important point. To address potential bias introduced by varying diagnostic sensitivities (e.g., microscopy vs. PCR) and temporal trends, we utilized a Bayesian mixed-effects modeling approach. By including 'Study ID' as a random effect, our model statistically accounts for study-level heterogeneity, including differences in diagnostic precision and the era in which the study was conducted. Effectively, the model treats these methodological differences as part of the "noise" inherent in global data, ensuring that the estimated effect of cat management (indoor vs. outdoor) is not driven by the diagnostic limitations of any single study or time period. We also repeated all analyses to include method as a fixed or random variable, which is what is now in the manuscript and none of our results had a material change. In addition, we also conducted analyses in two temporal clusters (≤2000 and ≤ 2010) with results provided in Table S5. As shown in Table S5, the odds of infection for outdoor-owned cats (OR = 2.92 for 2000+) remain remarkably consistent with our main findings. This stability across two decades, a period characterized by the rapid adoption of high-sensitivity molecular diagnostics confirms that our conclusions are not artifacts of shifting diagnostic methodologies or temporal trends.

Comment 3.3: "The dataset of the identified pathogens is also lacking, particular the indoor pathogens... there is a difference between unreported and negative..."

3.3 Response: We agree that "unreported" is not synonymous with "negative." Our analysis did not assume pathogens were absent if they were not reported; rather, our meta-analysis only utilized data where pathogens were actively sought, and denominators (total cats tested) were provided.

To address the smaller sample sizes in indoor cat studies and to prevent bias, we employed Rarefaction and Extrapolation curves (Figure 3 based on Chao1 estimators. This method is the gold standard for comparing groups with unequal sampling effort. It allowed us to statistically estimate pathogen richness for indoor cats, even with fewer studies, ensuring that our comparison of "indoor" vs "outdoor" diversity was standardized for sampling depth. We have clarified this in the Methods section to ensure readers understand that the results are not a byproduct of sampling bias.

Regarding the reviewer's concern about the smaller dataset for indoor cats: we accounted for this discrepancy using two robust statistical methods. First, our Bayesian hierarchical models generate 95% credible intervals that explicitly reflect the higher uncertainty associated with smaller sample sizes; the width of these intervals provides a transparent measure of our statistical power. Second, we used rarefaction and Extrapolation curves (Figure 3) based on the Chao1 estimator. This method standardizes 'pathogen richness' by sampling effort, allowing for a fair comparison between the groups regardless of the total number of individuals tested in each category. This ensures our conclusions about lower diversity in indoor cats are not a sampling artifact.

Comment 3.4 "...There are also major assumptions that weaken the study. The first is the assumption is that indoor cats are being provided with appropriate veterinary care."

3.4 Response: While indoor-only status generally implies greater human management (including presumed veterinary care), we acknowledge that this assumption may not hold uniformly across studies. We now explicitly state this limitation in the revised Discussion (Lines 261–263). Additionally, our categorization along a management gradient (indoor-only → outdoor-owned → unowned) reflects a spectrum of human intervention and allows us to assess relative differences without assuming perfect care or isolation for any group.

Lines 123-126 now read: *Because ownership-associated correlates, such as antihelminthic treatment, are rarely reported as continuous variables across studies and do not necessarily covary with free-roaming behaviour, we treated cat lifestyles as discrete categories that represent broad positions along a free-roaming continuum.*

Comment 3.5 "...**Secondarily, that the risk for each pathogen is equal globally…"**

3.5 Response: To rigorously address variation in pathogen risk across regions and pathogen types, we have now included both country *and* pathogen as random variables in the multiple pathogen Bayesian generalized linear mixed model (GLMMs) (see *Methods*, Lines 386–417). Country accounts for regional differences in surveillance intensity, public health infrastructure, pathogen ecology, and environmental conditions. Including the term

"Pathogen" accounts for differing transmission modes, host specificity, and detection probability among pathogens. This structure ensures that observed differences in infection risk by cat lifestyle are not confounded by the background variability in pathogen prevalence across locations or taxa. We believe these model refinements strengthen the robustness of our conclusions and directly address the concerns highlighted by the reviewer.

Comment 3.6: While understanding the importance of outdoor cats and zoonotic spillover is needed in the current literature, I unfortunately believe that this study is over-generalizing the population while not providing sufficient data to support the claims.

3.6 Response: We appreciate this concern and emphasize that our conclusions are not intended to imply a uniform or deterministic effect across all pathogens or contexts. Rather, our study evaluates whether unsupervised outdoor access represents a biologically plausible and consequential exposure pathway for zoonotic pathogens, which our results support while also demonstrating substantial pathogen-specific heterogeneity. Importantly, not all pathogens showed strong associations with outdoor access. Pathogens with predominantly environmental transmission routes, such as Giardia and Cryptosporidium, exhibited weak or inconsistent effects, underscoring that free-roaming is a plausible but not universal driver of infection risk. This heterogeneity is explicitly addressed through pathogen-specific analyses, rarefaction of helminth diversity, and mixed-effects Bayesian models that account for study-, country-, and pathogen-level variation and that include credible intervals that reflect variation. We would also like to highlight that we have compiled arguably the largest dataset of cat-associated pathogens to date, with the only other comparable study using 21 studies (Chalkowski et al. 2019). Together, these results argue against inappropriate overgeneralization and instead support an interpretation in which outdoor roaming increases relative zoonotic exposure risk through direct, feasible mechanisms, while recognizing that its relative importance varies among pathogens and settings which we have emphasised in lines 303-306 which read: *Pathogen abundance and diversity also vary strongly with climatic, latitudinal, and anthropogenic factors, producing distinct geographic pathogen landscapes [58] and altering the absolute risk associated with free-roaming across countries and regions.*

Part II – Major Issues: Key Experiments Required for Acceptance

Reviewer #1: NONE

Reviewer #2: N/A

Reviewer 3:

Reviewer #3:

3.7 Comment: 1) Compile a more complete list of indoor and owned outdoor cat pathogens.

Response 3.7: We have compiled and provided a comprehensive pathogen dataset in Supplementary Table S1, which includes all identified pathogens across studies reporting on indoor-only, outdoor-owned, unowned cats, shelter and owned cats without lifestyle information. This table represents data extracted from over 600 studies encompassing 153 pathogen species (118 zoonotic), and includes lifestyle categorization even where not reported. We hope this clarification improves the utility of the supplementary table and addresses the reviewer's concern.

3.8 Comment: 2) Use a different and smaller subset of cases where the method of identification is uniformed/known.

Response 3.8: We have rerun all analyses and included method as a fixed effect in the all pathogens model and for Bartonella, Leptospira and *T. gondii*, where different methodologies occurs and sample sizes were sufficient to permit a robust estimation and as a random effect where robust estimation could not be obtained, such as Cryptosporidium, Giardia and *T. cati*.

3.9 Comment: 3) Either include more countries or limit the review to a smaller subset of the globe for more accuracy.

Response 3.9: We appreciate this suggestion and agree that uneven geographic coverage is an important limitation of global reviews. Rather than limiting the analysis to a smaller subset of countries, which would have excluded a substantial proportion of available data, we addressed heterogeneity in sampling effort by including country as a random effect in all models. This approach allows baseline infection risk to vary among countries while retaining information from less well-represented regions. We have also added text to the Discussion (**lines 303-306, highlighted in 3.6 Response)** acknowledging remaining geographic gaps and highlighting the importance of improving representation in future work.

Part III – Minor Issues: Editorial and Data Presentation Modifications
Please use this section for editorial suggestions as well as relatively minor modifications of existing data that would enhance clarity.

Reviewer 1:

Reviewer Comment 1.1: Lines 56–71: The One Health framework is introduced effectively, with appropriate references. However, the focus shifts rapidly from wildlife to domestic animals without fully articulating the conceptual bridge. To include 1–2 sentences explicitly linking domestic free-roaming species to pathogen flow networks, perhaps referencing Wells et al. 2020 or Gibb et al. 2020.

1.1 Response: Thank you for this recommendation. We have addressed this with a comment with two bridging sentences, which now read:
(Lines 72-75): *Within altered landscapes, free-roaming domestic animals occupy a unique ecological position, functioning as high-contact bridge hosts that connect wildlife, human, and environmental pathogen pools, thereby facilitating pathogen circulation and amplification across otherwise discrete host communities(Wells et al. 2020; Gibb et al. 2020). By moving repeatedly between human-dominated spaces and wildlife habitats, domestic species can integrate multi-host transmission networks and reshape zoonotic risk at the human–wildlife boundary.*

Comment 1.2: Lines 69: I think that you should add this important reference as example to support your sentence: ”ectoparasites”. I would like to suggest:

Ancillotto, L., Studer, V., Howard, T., Smith, V. S., McAlister, E., Beccaloni, J., ... & Mori, E. (2018). Environmental drivers of parasite load and species richness in introduced parakeets in an urban landscape. Parasitology Research, 117(11), 3591-3599.

Response 1.2: Thank you for this important inclusion we had overlooked.
Lines 81-83 now read:” *...as pathogen-sharing between domestic animals and humans exceeds that between humans and wildlife for a broad range of pathogens, including viruses (6), helminths (7), and ectoparasites (Woolhouse et al. 2001, Ancillotto et al. 2018).*”

Comment 1.3: Lines 72–96: The authors frame free-ranging cats/dogs as “conduits” well. However, the Introduction currently emphasizes wildlife-to-human spillover more than human-to-wildlife or wildlife-to-domestic dynamics. To clarify early that this study examines risk amplification pathways, not only wildlife-origin spillover.

Response 1.3: We have addressed this with two comments in two paragraphs, which now read: (Lines 72-75): “… thereby facilitating pathogen circulation and amplification across otherwise discrete host communities (Wells et al. 2020; Gibb et al. 2020).”

Lines 90-92: *Consequently, free-roaming dogs and cats function as high-contact bridge hosts, linking wildlife reservoirs with human populations and amplifying opportunities for cross-species pathogen transmission.*

Comment 1.4 Lines 97–110: The hypothesis is clearly stated. However, the authors phrase the management gradient as categorical. In reality, management (indoors → supervised outdoors → unrestricted outdoors) may be continuous. Acknowledge that lifestyle categories may vary in enforcement and duration.

Response 1.4: We have addressed this with a comment twice in two paragraphs, which now read:
Lines (108-109) now read: *The intermediate group consists of outdoor-owned cats that are allowed to roam at rates that vary spatially and temporally.*

Lines (123-126) now read: *Because ownership-associated correlates, such as antihelminthic treatment, are rarely reported as continuous variables across studies and do not necessarily covary with free-roaming behaviour, we treated cat lifestyles as discrete categories that represent broad positions along a free-roaming continuum.*

Comment 1.5 Lines 113–131: The result that outdoor-owned cats have infection odds comparable to feral cats is compelling and well communicated. However: The authors should present absolute prevalence differences, not only odds ratios; The large variability among countries (OR 0.8–16.5) likely arises from lifestyle misclassification and vector presence but is not discussed here. To add a short text linking Figure 3 variation to: differences in rodent exposure; climate/latitudinal parasite gradients; cultural norms of cat care.

Response 1.5: We thank the reviewer for this constructive suggestion. To complement the odds ratio findings, we now report absolute prevalence estimates in the Results, which demonstrate that outdoor-owned and unowned cats have similarly elevated prevalence relative to indoor cats. *Lines 148-151 now read: Pooled prevalence estimates showed a similar pattern, with outdoor (18.24%, 95% CI:17.90–18.59) and feral cats (18.04%, 95% CI:17.82–18.26) exhibiting substantially higher overall positivity than indoor cats (8.26%, 95% CI: 7.95–8.59).*

We initially explored country-level variation in relative infection odds; however, these estimates were highly sensitive to sparse indoor sampling and lifestyle misclassification in several countries, leading to unstable or undefined values. To avoid over-interpretation of descriptive extremes, we removed the country-level relative risk analyses and the associated figure from the revised manuscript.

However, we have ensured that the discussion includes these points, lines 300-306 now read:

"Further complicating the issue of lifestyle classification is that there may be country-level differences in what the public perceives as an indoor versus an outdoor cat, which could be a complex result of pet-ownership norms and no-roaming initiatives. Pathogen abundance and diversity also vary strongly with climatic, latitudinal, and anthropogenic factors, producing distinct geographic pathogen landscapes [58] and altering the absolute risk associated with free-roaming across regions."

Comment 1.6 Lines 136–145: The rarefaction analysis is appropriate and well presented.

However, diversity is influenced not only by exposure but by diagnostic method heterogeneity. Microscopy underdetects cryptic helminths. To add a cautionary note acknowledging that observed diversity differences may be partly methodological.

Response 1.6: We now discuss this explicitly in paragraph 313-329 (text not repasted as it is the entire paragraph)

Comment 1.7 Lines 146–158: The authors state that ownership does not reduce infection risk for outdoor cats.
This is justified by results, but the tone could imply causality rather than correlation. To rephrase to:
"Ownership did not correlate with reduced infection risk when cats were allowed to roam freely."

Response 1.7: We have adjusted this paragraph in several locations to avoid implying causality.
Lines 183-194 now read: *This study demonstrates a strong association between unsupervised outdoor roaming and zoonotic pathogen infection risk in domestic cats.*

Lines 188-190: *These findings are consistent with previous smaller-scale studies of owned cats [20] and further underscore the association between free roaming and elevated zoonotic infection risk.*

Comment 1.8 Lines 170–206: The discussion of predator-prey pathogen transmission pathways is strong. However, behavioral variation among cats should be acknowledged: some cats hunt often; others rarely hunt; provisioning and age strongly influence hunting behavior.
Reference: Loyd et al. 2013, Kays et al. 2020 on variability in hunting intensity.

Response 1.8:
Lines 212-218 now read: *Therefore, even though hunting intensity and prey specialization vary among individual outdoor-owned cats [27,28], owner-perceived rates of ~1–15 wildlife kills per month would be sufficient for outdoor-owned cats to become infected through hunting, particularly given that owner reports underestimate true predation by approximately 80% [29]. These exposure levels are sufficient to rapidly exceed infection thresholds, helping to explain the comparable odds of infection observed between owned outdoor and unowned cats.*

Comment 1.9 Lines 294–310: The PRISMA approach is appropriate. Search terms ("cat," "parasite," "zoonosis") are too narrow and likely to underrepresent viral surveillance studies; vector-borne pathogen surveys; wildlife-predation-associated transmission research. To include additional search strings such as "Felis catus" + "pathogen", "domestic cat" + "infection prevalence", "helminth", "protozoa", "vector-borne".

Response 1.9:

The search strategy was defined *a priori* to align with the hypothesis-driven aim of evaluating zoonotic pathogen risk associated with domestic cats and the associated relationship to cat lifestyle. We therefore prioritised the terms "cat," "parasite," and "zoonosis" and grammatical variants, which explicitly reflect zoonotic framing and produced a large, manageable candidate set for title and abstract screening.*

Based on the reviewer’s recommendations, we assessed alternative host and pathogen-class terms (e.g., “domestic cat”, “Felis catus, “helminth,” “protozoa,” and “pathogen”) to evaluate their impact on retrieval (Table R1). These tests showed that some alternatives yielded comparable or smaller numbers of records, whereas broader terms such as “pathogen” resulted in very large increases in retrieval dominated by veterinary, experimental, or non-zoonotic literature that would be excluded under the predefined eligibility criteria. None of these alternatives provided a clear mechanism for preferentially identifying additional eligible zoonotic studies.

Importantly, since changing the selected search terms does not bias against any particular cat lifestyle (e.g., owned, free-roaming, or feral), and there is no *a priori* reason to expect systematic exclusion of studies based on lifestyle. Since the final search yielded over 2,100 screened records, we respectfully suggest there was substantial sensitivity and saturation. While we definitely agree that some eligible studies may not have been captured, further expansion of search terms into the more general terms would be expected to yield diminishing returns and would not materially alter inference regarding zoonotic risk or lifestyle associations.

Table R1. Web of Science search terms evaluated in response to reviewer comments and corresponding pre-screening record counts (2000–2025; search conducted December 26, 2025).

Search term	Returns
cat AND pathogen	5230
cat AND parasite	3535
cat AND protozoa	1143
cat AND helminth	664
cat AND zoonos*	1498
domestic cat AND zoonos*	492
Felis catus AND helminth	60
Felis catus AND protozoa	74
Felis catus AND parasite	268
domestic cat AND parasite	835

We have also addressed this point in the manuscript in lines 390-395, which now read:

We searched Web of Science and PubMed using the terms and grammatical variants “cat,” “parasite,” and “zoonosis,” which were selected a priori to align with the zoonosis-focused objective of the study while avoiding systematic bias related to cat lifestyle.

Comment 1.10: Lines 305–310: This classification (indoor-owned / outdoor-owned / unowned) is central, yet relies entirely on author-reported lifestyle, which may be ambiguous or culturally variable. Comparison with other literature, such as Chalkowski et al. 2019 highlighted strong misclassification biases in global cat lifestyle reporting; Hall et al. 2020 demonstrated that even “indoor cats” may spend up to 2–6 hours outdoors depending on region. State explicitly: whether ambiguous lifestyle categories were excluded or reclassified; how cases of “partially outdoor access” were handled.

Response 1.10: We thank the reviewer for highlighting the potential for misclassification bias in cat lifestyle reporting, particularly regarding the 'grey area' of partial outdoor access noted by Hall et al. (2020). We have updated the Methods section (Lines 305–312) to explicitly define our classification criteria. Specifically, any cat with unsupervised outdoor access (including those described as 'indoor-outdoor') was classified as 'outdoor-owned' to ensure a conservative estimate of the risks associated with roaming. Ambiguous cases, or those where authors did not provide a definitive lifestyle description, were excluded from our lifestyle-specific and rarefaction analyses to maintain the integrity of the categories. This approach aligns with the recommendations of Chalkowski et al. (2019) to prioritize clear-history cohorts when assessing global pathogen prevalence.

We have added additional details to our methods lines 405-414 now read: *To minimize misclassification bias [20], cats were only classified as 'indoor-owned' if authors explicitly classified them as indoor. Any owned cat described as having unsupervised outdoor access, including 'indoor-outdoor' cats or those with 'partial' access were categorized as 'outdoor-owned.' To ensure the robustness of the comparative analysis, cases where lifestyle was ambiguous or described as 'restricted' without further detail were excluded from lifestyle-specific analyses and retained only for general prevalence descriptions. Similarly, shelter cats with unknown histories were excluded from the lifestyle and rarefaction analyses to prevent the conflation of indoor and outdoor risk profiles. Only data from indoor-owned, outdoor-owned, and feral cats were included in analyses.*

Comment 1.11 Lines 312–326: The use of Bayesian GLMMs (MCMCglmm) is appropriate for hierarchical data. However: the prior specification (V=1, ν=0.02) may be insufficiently justified;

Comment 1.12: “...model convergence diagnostics are not described;

Comment 1.13: “the decision to treat all pathogens as a pooled group assumes biological equivalence, which is unlikely.”

Comment 1.14: To add: trace plots, ESS, and Gelman-Rubin diagnostics to supplement S.I.;

Response 1.11: We used the weakly informative default priors in MCMCglmm (V = 1, ν = 0.02) for the random effects structure, which are commonly applied in binomial GLMMs with hierarchical structure. These priors were selected to support convergence without

over-constraining the posterior, and we have now added a justification for this choice in the Methods section (see below). We appreciate the reviewer's suggestion to consider a stronger justification for our prior specification. To address this, we re-fit our full pooled model using a range of inverse-Gamma priors of varying informativeness. Posterior distributions for the key lifestyle effect remained consistent across priors, with pairwise posterior overlaps ranging from 96.3% to 97.7%. We have expanded upon this sensitivity analysis in our methods section.

Lines 433-440: *To evaluate the influence of prior specification on our results, we conducted a prior sensitivity analysis by fitting the same model under weak (v = 0.02), moderately informative (v = 1), and diffuse (v = 0.5) inverse-Gamma priors for the random effects. We calculated posterior overlap scores for the key parameter cat management, which exceeded 96% across all model pairs (weak vs. moderate: 96.3%; weak vs. diffuse: 97.7%; moderate vs. diffuse: 96.6%). This high degree of consistency demonstrates that the model inference and empirical data are robust to varying prior assumptions.*

Comment 1.12: "...model convergence diagnostics are not described;

Response 1.12: We have now explicitly described our convergence diagnostics in the Methods. Lines 440-443 now read: *Model convergence was assessed using trace plots, effective sample sizes, Gelman–Rubin diagnostics ($\hat{R}$), and Geweke diagnostics. For all models, fixed-effect parameters showed $\hat{R}$ values close to 1.0, high effective sample sizes (>8,000), and no evidence of non-stationarity, indicating adequate convergence (Table S4, Fig S2a-h).*

Comment 1.13: "the decision to treat all pathogens as a pooled group assumes biological equivalence, which is unlikely."

Response 1.13: We acknowledge the important point that pooling across pathogens assumes some level of epidemiological equivalence. To address this concern, we have revised our analyses to include pathogen identity as a random effect in all pooled GLMMs to account for pathogen-specific baseline prevalence, transmission ecology, and diagnostic variability. We also reiterate this point in the discussion.

Lines 303-306: *Pathogen abundance and diversity also vary strongly with climatic, latitudinal, and anthropogenic factors, producing distinct geographic pathogen landscapes [58] and altering the absolute risk associated with free-roaming across regions.*

Lines 331-333: *The pathogens identified here span a wide range of public health importance, from rare but highly lethal infections, such as rabies virus, to highly prevalent pathogens, such as T. gondii, and many others for which human health impacts remain incompletely characterized[60].*

Comment 1.14 To add: trace plots, ESS, and Gelman-Rubin diagnostics to supplement S.I.;
Response 1.14: These are all included and provided as Table S2 and Figure S2.

Comment 1.15 "... pathogen-level modeling for additional taxa where possible (e.g., Anaplasma, FeLV, FIV, Aelurostrongylus abstrusus), which exist in literature with sufficient prevalence reporting"**.**

Response 1.15: We have reviewed the dataset for pathogens with sufficient prevalence and lifestyle data for modelling. In addition to *Toxoplasma gondii*, *Toxocara cati*, Bartonella, and Leptospira, we have now added pathogen-specific models for: Cryptosporidium spp, Ancylostoma spp. and Giardia. These new models and results are included in the results, methods and Figure 2. For Anaplasma and *Aelurostrongylus abstrusus*, data were too sparse across lifestyle categories to support pathogen-specific inference, though we agree these are important candidates for future study.

Comment 1.16 Lines 315: All the R codes used in this study must be added in the supplementary materials (…well commented)..
Response 1.16: A well-annotated Markdown file is included in the submission

Reviewer 2:

Reviewer #2: Introduction

Comment 2.1: Line 57 It may not be necessary for the audience of this journal but does One Health need a brief explanation?
Response 2.1: We have provided a definition within the text. Lines 64-66 now read:

In the One Health framework, which emphasizes the interconnectedness of human, animal, and environmental health, wildlife is frequently identified as a primary source of emerging zoonotic diseases.

Comment 2.2: Line 73 This first sentence in P2 should have a citation.
Response 2.2: We have now added citations to support this statement.
Line 87-90 now reads: *Among domestic animals, dogs and cats have high societal value and emotional familiarity, resulting in frequent close contact with humans. Emotional familiarity with companion animals can suppress risk perception, allowing zoonotic exposure pathways to persist largely unmitigated* (Stull et al. 2012, Overgaauw et al. 2020).

Stull, J. W., Peregrine, A. S., Sargeant, J. M., & Weese, J. S. (2012). Household knowledge, attitudes and practices related to pet contact and associated zoonoses in Ontario, Canada. *BMC Public Health*, *12*, 553. https://doi.org/10.1186/1471-2458-12-553

Overgaauw, P. A. M., Vinke, C. M., van Hagen, M. A. E., & Lipman, L. J. A. (2020). A One Health Perspective on the Human–Companion Animal Relationship with Emphasis on Zoonotic Aspects. *International Journal of Environmental Research and Public Health*, *17*(11), 3789. https://doi.org/10.3390/ijerph17113789

Comment 2.3 Was there any indication of how often owned cats receive veterinary care in these countries?

Response 2.3: We thank the reviewer for this comment as our use of the term management would include veterinary care, so we have modified our text in the introduction to ensure this is clear. Only a subset of studies reported information on deworming, and reporting was not sufficiently consistent or detailed to allow inclusion as a covariate. We have revised text in the Introduction and Discussion to clarify this limitation.

Lines 123-126: *Because ownership-associated correlates, such as antihelminthic treatment, are rarely reported as continuous variables across studies and do not necessarily covary with free-roaming behaviour, we treated cat lifestyles as discrete categories that represent broad positions along a free-roaming continuum.*

Lines 259-263: *At the pathogen-specific level, owned outdoor cats exhibited slightly lower average odds of shedding T. cati than unowned cats, although this difference was not statistically significant. This pattern could plausibly reflect differences in hunting behaviour or antihelmintic use; however, deworming protocols and treatment frequency were rarely reported and could not be evaluated in the analysis.*

Results

Comment 2.4: Line 117 So, only a fraction of papers reported outdoor access % for their study country?

Response 2.4: Yes. A substantial proportion of studies did not report cat lifestyle in sufficient detail. As noted in the Methods (lines 408 – 411), studies in which lifestyle was ambiguous or described only as "restricted" were excluded from lifestyle-specific analyses and retained only for general prevalence summaries. Shelter cats with unknown histories were likewise excluded from lifestyle and rarefaction analyses to avoid conflating indoor and outdoor exposure profiles. Despite this underreporting, a large number of studies (435 of 606 eligible papers) provided sufficient lifestyle information, allowing for robust comparative analyses. This point is now given in Methods and is also explicitly acknowledged in the Discussion, lines 294-296) now read:

Lines 408-411: *To ensure the robustness of the comparative analysis, cases where lifestyle was ambiguous or described as 'restricted' without further detail were excluded from lifestyle-specific analyses and retained only for general prevalence descriptions.*

Lines 293-295: *Of the 606 papers that met the eligibility criteria, only 435 provided sufficient information on the lifestyle of owned cats to be included in the analyses.*

Comment 2.5: Line 138: What is the most common type of helminth pathogen by group? Does it pose risk of zoonotic transmission?

Response 2.5: This was a very good reminder to report on this, and we now discuss this in lines 173-175, which now state: *The most common helminth pathogens were the zoonotic T. cati and Ancylostoma, which together accounted for 35.6% and 14.6% of all infections, respectively.*

Discussion

Comment 2.6:Line 168 This sentence should have a citation.
Response 2.6: Corrected, lines 206-208 now read: *T. cati* and *T. gondii* are transmitted through both environmental and trophic routes [21,22], while *Leptospira* infections are typically acquired through contact with the urine or blood of infected prey, with rodents serving as major reservoirs [23].

Comment 2.7: Line 187 Should this paragraph (p 4) be moved up before p 2?
Response 2.7: Thank you, this was an awkward placement as we did present this summary in the results. We have restructured the paragraph, removing that statement and revising the following statement. Lines 230-235 now read: *Although isolation of a pathogen from a cat alone does not demonstrate reservoir competence, the detection of more than 100 pathogens with zoonotic potential spanning viral, helminthic, protozoal, and bacterial taxa warrants concern. Frequent interactions with wildlife and domestic animal reservoir hosts create repeated opportunities for spillover and spillback, potentially enabling cats to assume epidemiologically meaningful roles, particularly for generalist pathogens with established or emerging zoonotic potential.*

Comment 2.8: Should the discussion address the most prevalent pathogens that are problematic (easily transmissible, symptomatic etc.) for humans?

Response 2.8: Yes thank you for highlighting this omission.
Lines 201-208 now read: *Consistent with their substantial zoonotic burden, a large proportion of studies focused on pathogens with well-established relevance to human health, including Toxocara cati, T. gondii, Ancylostoma spp., Cryptosporidium spp., Giardia spp., Leptospira spp., and Bartonella spp., enabling estimation of pathogen-specific odds ratios. For the majority of these pathogens, transmission is likely to be accelerated by outdoor access. T. cati and T. gondii are transmitted through both environmental and trophic routes [21,22], while Leptospira infections are typically acquired through contact with the urine or blood of infected prey, with rodents serving as major reservoirs [23].*

Comment 2.9: This should report years searched, range for publications.
Response 2.9: Lines 395-396 now read: *Searches were restricted to studies published from 1980 to the present.*

Comment 2.10: Figure 3 The risk of infection in just 2 of the 4 high countries is addressed in the discussion. Why might it also be high in Chile and Transylvania?

Response 2.10: We have removed the relative risk analysis and associated discussion. Relative risk was presented as a descriptive statistic and was not derived from the fitted models. This comment highlighted the limitations of this metric. In particular, relative risk estimates are sensitive to low prevalence values, uneven sampling effort, and aggregation across pathogens with distinct ecologies and transmission pathways. Additionally, classification of cat lifestyle for owned animals relies largely on owner-reported information, for which no objective or standardized measure of reporting accuracy exists across studies or

regions. Any attempt to explain these differences would therefore be speculative and would not meaningfully advance the study's primary objectives. We therefore elected to remove this analysis to improve clarity and avoid overinterpretation, consistent with the modelling framework used elsewhere in the manuscript.

R2 Response:

Comment 2.11 Table 1 supplement. There are no shelter or unknown columns yet that is mentioned in table description. This may also need more description or explanation as it appears the prevalence for some pathogens is 100% of the tested sample? Maybe sample size needs to be included?

Response 2.11: We have now corrected the legend and include sample size to ensure that readers understand some of the high values, such as 100%.
Lines 697-700: ***Table S1.*** *Summary of pathogen prevalence (%) with 95% confidence intervals and total number of domestic cats sampled (n), stratified by lifestyle category (feral, outdoor-owned, indoor, shelter, and unknown). Cells are left blank where no prevalence data were identified for a given pathogen–lifestyle combination.*

Reviewer 3:

Comment 3.10: 1) Expand on line #74, on how owned animals leads to regular human contact and potentially reduced vigilance toward zoonotic disease risk. I thought that the hypothesis was that owned animals receive a higher degree of vigilance and veterinary care?

Response 3.10: Thank you for the suggestion of clarity, as this was a very important point. Lines 87-90 now read: *Among domestic animals, dogs and cats have high societal value and emotional familiarity, resulting in frequent close contact with humans. Emotional familiarity with companion animals can suppress risk perception, allowing zoonotic exposure pathways to persist largely unmitigated* (Stull et al. 2012; Overgaauw et al. 2019).

Lines 110-113: *Although these outdoor-owned cats receive consistent feeding, varying levels of veterinary care, and partial confinement, they still interact with wildlife, making it unclear whether this partial management meaningfully reduces the risk that owned outdoor cats act as conduits for zoonoses of wildlife origin.*

Lines 123-126: *Because ownership-associated correlates, such as antihelminthic treatment, are rarely reported as continuous variables across studies and do not necessarily covary with free-roaming behaviour, we treated cat lifestyles as discrete categories that represent broad positions along a free-roaming continuum.*

Comment 3.11: Expand on line #221, what is the importance of echinococcus multilocularis eggs that was necessary to specifically mention?
Response 3.11: We appreciate the reviewer's suggestion to expand on the importance of this pathogen. Although not among the most frequently detected parasites, *Echinococcus multilocularis* eggs are environmentally persistent and associated with alveolar

echinococcosis, a severe and potentially fatal zoonosis. Their detection in cat feces, therefore, warrants specific mention in the context of surveillance and zoonotic risk, particularly given the traditional association of this parasite with wildlife reservoirs.

Lines 263-268 now expand upon this: *Fecal samples from cats recovered over 90 distinct pathogens, including Echinococcus multilocularis eggs, which is of particular note due to the severe zoonotic consequences and immediate infectivity of this cestode, highlighting the need for enhanced surveillance to understand how domestic cats may contribute to pathogens traditionally associated with wildlife* (47).

Comment 3.12 Expand on limitations of language barrier to the papers included in the study.
Response 3.12: Lines 396-398 now read: *Non-English studies were screened using Google Translate, and studies for which automated translation did not permit reliable data extraction were excluded (Fig. S1, Table S2, S3).*

Comment 3.13 Additional references for line #234, stating that "cats acting as primary animal source of human exposure in some locations" but only adding in a reference for a very specific region of the world. It would be helpful that if you make this claim, to add literature to support it more globally.

Response 3.13: We have now strengthened the evidence of this claim with specific examples, lines 278-281 now read: *Cat-associated rabies transmission is a growing public health concern globally [50]. In the US, cats are the most common rabies-positive domestic animal [51], constituting 2.5 times the exposure risk relative to wildlife in Pennsylvania [52], and in Colombia, cats are the primary source of human rabies transmissions [53].*

Comment 3.14 Define burn-in and thinning in the statistical analyses.

Response 3.14: Thank you for highlighting this point of confusion, we have defined this now. Line 428-431 now read: *All models assumed a multinomial distribution and were run for 400,000 iterations, with the first 100,000 iterations discarded as burn-in to allow chain convergence, and a thinning interval of 100 iterations applied to reduce autocorrelation among retained samples.*

Comment 3.15 Methods to go before results? The flow appears off with the methods being after the discussion.

Response 3.15: We were following the manuscript organization as per the submission instructions: Introduction > Results > Discussion > Materials and Methods. We can alter as the editorial office prefers.

Comment 3.16 Clarify on line #121 onwards, is the odds of infection for ALL pathogens or just bartonella, leptospirosis, toxoplasma, and toxocara?

Response 3.16: Thank you for highlighting this, as it was unclear as written. We have clarified this in multiple locations.
Lines 143-144 now read: The posterior mean log-odds of infection pooled across all pathogens were lowest for owned indoor cats (β_{indoor} = -3.21, 95% Credible Interval (CrI): -3.73 – -2.68). While outdoor-owned cats exhibited significantly higher pooled infection risk …"
Line 151: "When expressed as odds ratios (OR), the odds of infection with any pathogen were..".

Comment 3.17 In figure 3, a location is labelled as "Europe", but there are individual countries that are referred to in Europe e.g., Finland, Greece, Germany, Ireland, Netherlands.

Response 3.17: Figure 3 was removed as part of a broader revision in which relative risk estimates were no longer presented due to instability and interpretability concerns at the country level. As a result, the regional versus country labelling issue no longer applies.

Figure resubmission:
While revising your submission, we strongly recommend that you use PLOS's NAAS tool (https://ngplosjournals.pagemajik.ai/artanalysis) to test your figure files. NAAS can convert your figure files to the TIFF file type and meet basic requirements (such as print size, resolution), or provide you with a report on issues that do not meet our requirements and that NAAS cannot fix.

Response: Figures 1-3 have been run through the PLOS NAAS tool and saved. When retested passed the NAAS requirements.
